# Heterogeneity and state dependence in firms’ access to bank credit

**DOI:** 10.1007/s11187-021-00545-x

**Published:** 2021-10-21

**Authors:** David Aristei, Gabriele Angori

**Affiliations:** 1 University of Perugia, Perugia, Italy; 2 University of Ferrara, Ferrara, Italy

**Keywords:** Access to credit, Financing constraints, Bank lending, State dependence, SMEs, G32, G21, D25, O16, C33

## Abstract

This paper investigates firms’ access to bank credit in eleven euro area countries over the periods 2014–2019. Exploiting firm-level longitudinal data, we analyse loan demand and credit rationing probabilities, accounting for sample selection, unobserved heterogeneity and state dependence. Empirical results show that small and informationally opaque businesses, with deteriorated public support and credit history, face greater difficulties in obtaining bank loans. Furthermore, we provide evidence of a significant degree of state dependence in access to credit. In particular, firms that have already experienced credit restrictions are more likely to face further constraints, while enterprises that applied for bank financing in the past seem to have easier access to credit. Focusing on the subset of firms actually needing additional bank financing, we also find that past credit restrictions significantly reduce their current demand, providing evidence of a significant discouragement effect.

**Supplementary Information:**

The online version contains supplementary material available at 10.1007/s11187-021-00545-x.

## Abstract (plain language summary)

Access to bank credit is a key driver of firms’ survival and growth, especially in bank-based financial systems. Building on detailed longitudinal survey data, this study investigates the determinants of access to bank financing by firms in the euro area, both in a static and dynamic setting. Most importantly, we account for the intertemporal nature of firms’ loan demand behaviour and banks’ credit granting choices and show that they are both characterized by significant persistence over time. We also find that repeated lending interactions enhance access to credit and that firms needing additional financing tend to be discouraged from applying after having experienced an actual credit restriction. Our findings shed light on the existence of substantial and persistent financial frictions in banking markets, which play a key role in amplifying adverse economic shocks, and call for policy and regulatory interventions to improve firms’ access to credit and limit systemic vulnerabilities.

**Supplementary Information:**

The online version contains supplementary material available at 10.1007/s11187-021-00545-x.

## Introduction

Access to bank credit is one of the key drivers of firms’ survival and growth especially in countries with a bank-based financial system, where the recourse to alternative external financing sources by small and medium enterprises (SMEs) is limited. The global financial crisis has provided a clear reminder that financial frictions exacerbate firms’ credit access difficulties and hamper their investment and growth opportunities. In crisis periods, heightened financing imperfections and a weakened financial sector amplify adverse shocks and increase their persistence over time, with long-lasting adverse effects on credit allocation and real economic activity (Brunnermeier et al., 2013). Furthermore, as pointed out by Cowling et al. (2012), the way firms and banks react to a recessionary environment is different and not synchronized, leading to prolonged disequilibrium in the bank lending market.

Previous studies have provided large empirical evidence on firms’ access to credit, by assessing the determinants of loan demand and financing constraints mainly in a static context (Beck et al., 2006; Brown et al., 2011; Cole & Sokolyk, 2016). However, financial constraints, either in the form of loan rejection or of restrictions in the quantity and price dimensions, lead firms to cut back or resize planned investments and have negative impacts on production and sales. This may in turn trap firms in a long-lasting credit restriction state due to a self-reinforcing deterioration of their collaterisable net worth (Gertler and Gilchrist, 1994). At the same time, past credit restrictions may also have a negative signalling effect about firm riskiness that, under imperfect information and screening technologies, makes credit access difficulties persistent over time. Nevertheless, as discussed in Pigini et al. (2016), very limited attention has been paid in the literature to assess the actual persistence of financial frictions and to formally test whether past credit restrictions have a causal effect on firms’ current access to credit.

In this paper we assess the main drivers of firms’ access to bank financing in the euro area, with a specific focus on investigating the dynamics of loan demand and credit rationing. The empirical analysis is carried out on longitudinal firm-level data taken from “Survey on Access to Finance of Enterprises” (SAFE), which provides detailed information on firms’ characteristics and financial structure and on their use of external financing. This information allows us to define direct indicators of access to finance and to jointly analyse the determinants of firms’ loan demand behaviour and banks’ credit granting decisions in eleven euro area countries over the periods 2014–2019. To the aims of our analysis, the longitudinal structure of the data further allows us to control for observed and unobserved firm-level heterogeneity and to assess state dependence in loan demand and rationing probabilities.

This study contributes to the empirical literature on firms’ access to credit in a number of ways. First, we analyse firms’ financing conditions in a period characterized by a slow, but constant recovery after the global financial crisis, in which access to bank lending may represent a financial accelerator of firms’ investments. To this aim, we exploit the information on loan application outcomes provided by the SAFE survey and consider alternative direct indicators of credit rationing, taking into account different degrees of financing constraints in terms of both credit rejection and quantity restrictions. Second, we highlight the necessity of tackling non-random sample selection bias and accounting for unobserved heterogeneity in order to properly model firms’ access to bank financing. Third, and most important, our study is the first to fully address the intertemporal nature of firms’ loan demand behaviour and banks’ credit granting choices. One of the main aims of our analysis is to identify and consistently estimate true state dependence in firms’ access to credit demand, net of the other sources of persistence. In this regard, controlling for unobserved time-invariant firm heterogeneity, addressing sample selection bias and handling the endogeneity of initial conditions, we provide strong empirical evidence that past credit access conditions exert a significant causal effect on future credit demand behaviour and financing constraints. Specifically, we find that the experience of a restriction in access to credit significantly increases the likelihood of facing credit restrictions again in the next period. Furthermore, past loan demand not only increases the probability of applying for additional financing in the future, but it also reduces the probability of rationing. This evidence supports the view that the information accumulated over time through repeated interactions with the firm allows banks to address the informational opacity problems typical of small business lending and mitigates credit rationing. Finally, we point out a significant credit discouragement effect when we focus on the subsample of firms that actually need additional bank financing: in this subsample, past credit restrictions appear to significantly curb firms’ current loan demand behaviour.

The remainder of the paper is organized as follows. Section 2 reviews the literature on firms’ access to credit. Section 3 describes data and variables, while Sect. 4 presents the econometric methods. Estimation results are reported and discussed in Sect. 5. Section 6 provides a number of robustness checks on the main empirical findings and Sect. 7 offers some concluding remarks.

## Literature overview

### Financial frictions in credit markets

A large body of theoretical and empirical literature has identified financial frictions as main determinants of credit market failures and financial constraints. When *ex ante* information is asymmetric, borrowers have private information about their creditworthiness that lenders cannot observe, leading to a market equilibrium characterized by adverse selection and credit rationing (Stiglitz & Weiss, 1981). At the same time, *ex post* frictions, such as moral hazard, costly monitoring, and limited contract enforceability, significantly hinder firms’ access to credit (Boot & Thakor, 1994; Boyd & Smith, 1994; Cooley et al., 2004). Hyytinen and Väänänen (2006) and Kirschenmann (2016) show that asymmetric information is the main source of frictions in the market for small business finance, while Nikolov et al. (2021) point out that firms’ financing constraints are mainly driven by agency conflicts and moral hazard.

The presence of *ex ante* information and/or *ex post* incentive problems motivates collateral as a widely used debt contracting feature (Berger et al., 2011a). On the one hand, lenders may require collateral to sort observationally equivalent loan applicants, as borrowers with higher quality projects have an incentive to signal their safety by choosing secured debt with lower risk premiums. On the other hand, lenders may require observably riskier borrowers to pledge collateral to reduce loss given default. Despite the debate upon which theory empirically dominates is still open, previous studies suggest that collateral, through its impact on informational opaqueness, represents an effective solution for credit rationing in SME lending (Steijvers & Voordeckers, 2009). However, as discussed in Berger et al. (2011b), collateral is costly for both banks and borrowers, and its pervasive use may have adverse macroeconomics consequences, amplifying business cycle fluctuations through procyclical changes in access to credit. Furthermore, firms characterized by higher informational opacity, like small firms, start-ups and high-tech firms, are often unable to provide the required collateral and this exacerbates their difficulties in accessing credit, especially in crisis periods (Cowling et al., 2018).

Banks may use alternative instruments to reduce asymmetric information issues and avoid credit rationing in presence of binding collateral constraints. In particular, a relational approach to lending, based on the production and accumulation of soft information through multiple firm-bank interactions over time, allows reducing informational asymmetries and improves banks’ *ex ante* screening and *ex post* monitoring (Berger & Udell, 2002).^1^ Relationship lending technologies have been found to significantly mitigate financial constraints in periods of financial distress, especially for small and informationally opaque firms (Angori et al., 2019; Beck et al., 2018). Degryse et al. (2019) also show that banks facing higher capital requirements provide easier access to unsecured funding to firms with stronger lending relationships during crisis times.

A prominent role in addressing market failures in firm financing is also played by public financial support measures. A growing literature has provided support to the effectiveness of public credit guarantee schemes, introduced worldwide to allow lenders to share with the government loan default risks through the public provision of (partial or total) guarantees (see Beck et al., 2010). Publicly founded loan guarantee programs targeted at smaller businesses are found to be a viable option to promote access to debt finance for constrained businesses lacking collateral assets and/or financial track records (Cowling, 2010; Cowling & Siepel, 2013; Riding et al., 2007), even though the degree of credit additionality is significantly affected by the different guarantee mechanisms and coverage ratios of the schemes (Boschi et al., 2014).

### Persistence in firms’ access to credit

Previous literature has analysed firms’ access to credit mainly in a static framework and very limited attention has been paid to the possibility that firms can be locked in a credit restriction state over time. In this latter respect, Jiménez et al. (2012) show that loan supply restrictions are binding and firms that experienced a rejection cannot offset the resultant reduction in credit availability by applying to other banks, especially in periods of tighter monetary and economic conditions. Similarly, Pigini et al. (2016) provide evidence that firms' financing constraints are characterized by a significant degree of state dependence, which is particularly relevant for larger firms and is heightened by global liquidity shocks.

Persistence of credit demand and financing constraints over time may depend on observable firm characteristics, as well as on unobserved heterogeneity and true state dependence. Firms’ access to credit may be affected by unmeasured firm-specific variables that are not influenced by past credit experiences. If these unobserved factors are correlated over time and are not properly controlled for, they give rise to spurious state dependence (Heckman, 1981): past demand and financing constraints may appear to affect current access to external finance only because they reflect the effect of temporally persistent unobserved heterogeneity. True state dependence refers instead to the genuine causal effect of past credit access conditions on current demand behaviour and financing constraints. A firm that applied for credit or that experienced a credit restriction in the past will have a higher demand probability or will be more likely to face credit access difficulties in the future than an otherwise identical firm that did not apply or was not constrained, respectively. As discussed in Pigini et al. (2016), true state dependence in credit restriction can be explained by the negative impact of financing constraints on firm net worth and by the adverse signalling about firm creditworthiness. First, a restricted access to credit lead firms to cancel or postpone planned investments and reduce their ability to pursue attractive investment opportunities (Levenson and Willard, 2000; Campello et al., 2010). Constrained firms may also have to cut production, determining a decline in net that further reduces their probability of obtaining credit in the future. A second mechanism pertains to frictions in information transmission and processing, which make banks’ screening technologies and scoring models to be characterized by a significant degree of memory. This implies that previously credit constrained firms are more likely to be restricted again in the future as banks tend to keep their negative creditworthiness assessments from one period to the other, due to the lack of updated information and to the stickiness of their screening tests. At the same time, financial frictions may also determine a significant degree of state dependence in the demand for bank credit. As discussed in Kon and Storey (2003), imperfect screening by banks, heterogeneous application costs, limited availability of collateral and the lack of alternative financing sources significantly affect firms’ loan application behaviour and its persistence over time.

### Credit discouragement and self-selection into credit demand

A recent strand of literature has explored the demand-side constraints on access to credit and focus on credit discouragement that is on the decision by a firm needing finance not to apply for a loan as it feels its application will be rejected (Cole & Sokolyk, 2016).

Credit discouragement theory has been formalized by Kon and Storey (2003), who point out that application costs for borrowers and imperfect screening by banks play a crucial role in determining credit discouragement. Under imperfect information, banks do not know the quality of borrowers and some creditworthy firms may choose not to apply for credit because the cost of application is too high and the return is no longer sufficient to cover borrowing costs.

Han et al. (2009) show that creditworthy borrowers are less likely to be discouraged, especially in concentrated banking markets and when lending relationships are relatively longer, arguing that credit discouragement can be viewed as an efficient self-rationing mechanism. Similarly, Freel et al. (2012) point out that smaller firms, those pursuing cost-focused strategies and those lacking established banking relationships are more likely to be discouraged. Cowling et al. (2016) find that discouraged borrowers are smaller, younger and with poorer growth performance than loan applicants, but they are also more informed about their actual riskiness, suggesting that they are rationally self-rationed from the credit market. Furthermore, they point out that, during economic downturns, relationship lending significantly mitigates credit discouragement by reducing noise and uncertainty in the bank lending market. Rostamkalaei et al. (2020) address the role informal turndowns as an additional demand-side constraint and show that more established businesses tend to suspend loan applications on the basis of informal talks with the bank rather than to self-ration themselves.

The interpretation of credit discouragement as a self-rationing mechanism is strictly intertwined with the importance of addressing self-selection into credit demand to properly analyse banks’ actual credit granting decisions. Brown et al. (2011) and Aristei and Gallo (2016, 2021) provide evidence of a significant self-selection effect and point out that those firms which are more likely to have an application rejected are also more likely to refrain from applying.

Sticky screening technologies and costly application procedures motivate the existence of credit discouragement effects also in an intertemporal setting, as firms’ current loan demand behaviour may be significantly influenced by past credit restrictions. In this sense, as pointed out by Pigini et al. (2016), firms that have already experienced restrictions in their access to credit may be discouraged from applying for additional financing in the future, as they anticipate their higher rationing probability.

## Data and measurement

### Data sources

Our primary data source is the “*Survey on Access to Finance of Enterprises*” (SAFE), run jointly by the European Commission (EC) and the European Central Bank (ECB). The survey is carried out every 6 months since 2009 and provides detailed microdata on firms’ financing conditions in the euro area, as well as (mostly qualitative) information on a wide variety of firm characteristics. The sample is composed of non-financial firms, randomly selected from the Dun & Bradstreet business register, and is stratified by country, firm size class and economic activity. The survey mainly focuses on SMEs, and sample allocation by size classes is defined to offer comparable precision for micro-, small- and medium-sized firms; a sample of large enterprises is also included to compare developments for SMEs with those of large firms. To obtain more accurate estimators of semester-to-semester changes, the sample includes a rotating panel component, which includes those enterprises that are interviewed in at least two not necessarily consecutive waves. The panel component consists of around 60% of the surveyed firms in each wave and its relevance has significantly increased over time.^2^

In this study, we consider firms from eleven euro area countries (Austria, Belgium, Germany, Spain, Finland, France, Greece, Ireland, Italy, the Netherlands and Portugal) and focus on survey waves from 11 (April–September 2014) to 21 (April–September 2019), due to the availability of some key variables. Moreover, we exploit the longitudinal dimension of the SAFE and assemble unbalanced panels of firms (with a six-month time frame) to maximize the number of observations and limit survivorship bias and attrition.

We integrate the SAFE survey data with additional yearly information from the *ECB Statistical Data Warehouse*, the *Key Statistics* of the European Association of Co-operative Banks (EACB) and the World Bank’s *World Development Indicators* and *Doing Business* datasets, in order to account for differences in credit market characteristics and institutional factors at the country-level.

### Measuring access to bank credit

We exploit the detailed information provided by the SAFE to define direct measures of access to bank credit based on the results of firms’ actual loan applications.^3^ Specifically, we build a binary indicator of loan demand (*Loan demand*) equal to one if the firm applied for a bank loan in the past 6 months.^4^ Conditional on loan demand, we define alternative direct measures of financing constraints. We define a first dummy variable (*Rationing*) identifying those firms having their loan application rejected by the bank. We then create a second binary variable (*Rationing* 2) equal to one if the firm was either denied credit or received a limited part (i.e. below the 75%) of the requested amount. Finally, we build a broader indicator (*Rationing* 3) equal to one if the firm was either denied credit or did not receive the requested amount in full. As discussed in García-Posada Gómez (2019), these two-latter indicators allow extending the definition of credit constraints to account for different degrees of restrictions in the quantity of credit granted by the bank (i.e. strong and weak quantity rationing). We do not consider as rationed those firms that did not apply for a bank loan as they feared their application would be rejected (i.e. discouraged borrowers).^5^ Firms choosing not to apply for a bank loan, either because they are discouraged from applying or because have sufficient internal funds, are thus classified as non-demanding firms. At the same time, we exclude from the estimation sample those firms whose loan application is still pending and those that refused the loan because the cost was too high (i.e. price rationing). This allows us to properly assess both the firm’s loan demand behaviour and the bank’s actual decision to grant or (fully or partially) reject the application.

Table 1 confirms that bank loans are one of the most important sources of financing for the non-financial corporate sector: about 58% of the enterprises in the sample consider bank loans as relevant for their businesses and this proportion exceeds 60% in Belgium, Spain, Finland, France and Portugal. France, Italy and Spain are also characterized by the highest proportions of firms applying for a bank loan, whereas Ireland, the Netherlands and Greece show the lowest application rates. Firms in Greece display the highest difficulties in access to bank financing, with credit rationing rates ranging from 19%, when only loan rejection is considered, to more than 52% when strong and weak quantity restrictions are considered. Moreover, Spain, Ireland, the Netherlands, Portugal and Italy are characterized by remarkable proportions of quantity constrained applications, whereas Belgium, Austria and Germany display the lowest denial rates. This evidence confirms that the incidence of credit constraints among firms is highest in those countries severely affected by the 2008–2009 global financial crisis and by the 2010–2012 euro area sovereign debt crisis.

**Table 1:** Bank loans relevance, demand and rationing by country

	*Bank loans relevance*	*Loan demand*	*Rationing*	*Rationing 2*	*Rationing 3*
Austria	53.92	28.68	2.65	7.73	12.65
Belgium	61.43	37.24	3.89	7.83	12.71
Germany	53.08	28.51	2.56	5.13	9.32
Spain	60.26	38.50	4.66	14.70	26.33
Finland	67.59	28.65	6.18	9.70	12.44
France	64.96	42.79	5.05	8.24	12.15
Greece	55.00	26.44	19.24	37.58	52.16
Ireland	57.66	21.07	8.11	14.38	25.07
Italy	58.45	40.92	5.31	12.92	21.22
Netherlands	52.10	20.33	11.77	16.85	24.95
Portugal	64.25	30.14	4.72	13.21	22.48
Total	57.96	34.59	4.79	10.36	16.96

Proportions are expressed in percentage terms and are computed on the longitudinal sample of firms interviewed in at least two not necessarily consecutive waves using sample weights. The proportion of firms having applied for a bank loan is computed on the subsample of firms for which bank loans are relevant. The proportion of credit rationed firms is computed on the subsample of firms that have applied for a loan

**Fig. 1:**
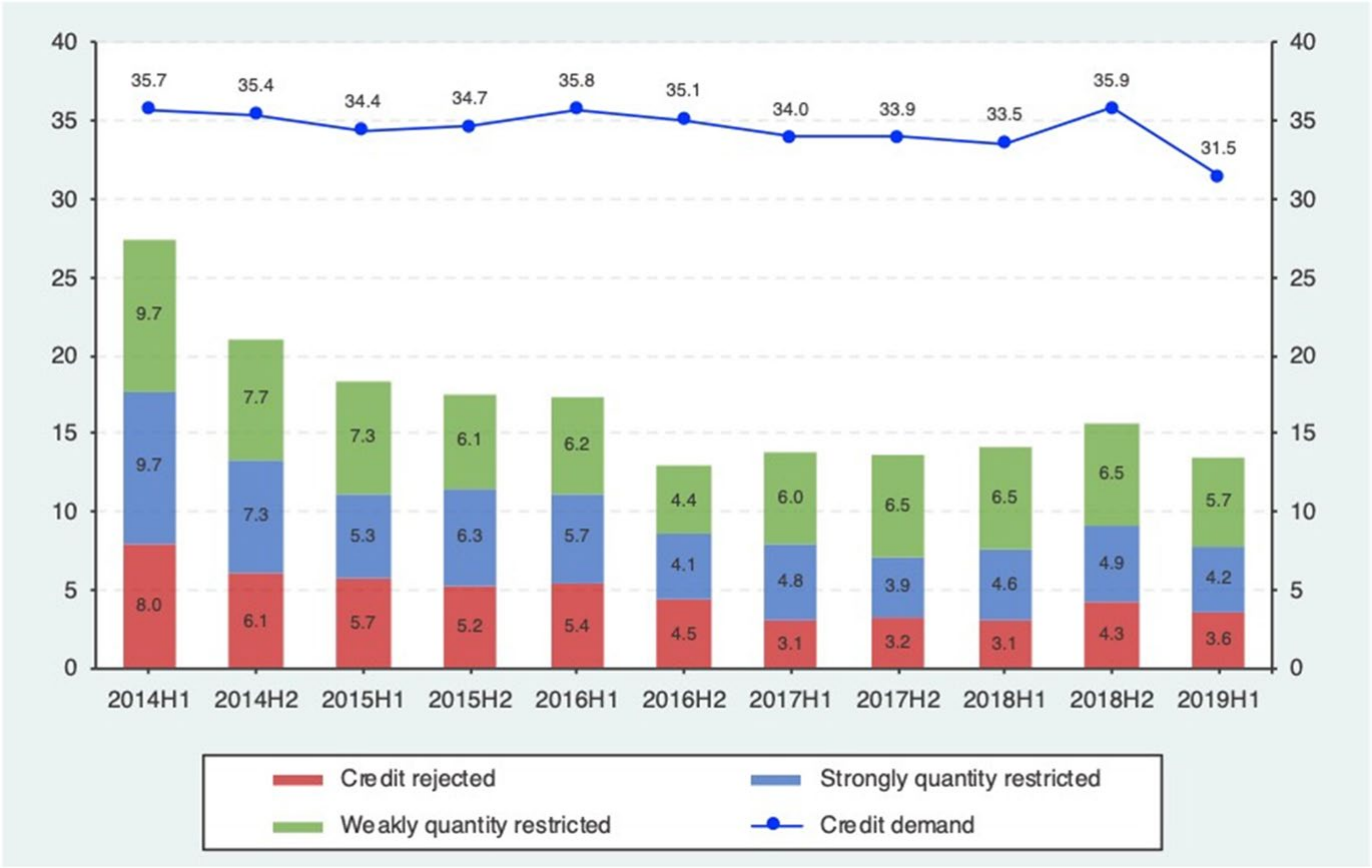
Proportions of firms applying for a bank loan and of credit rationed firms by survey wave. Proportions are expressed in percentage terms and are computed on the longitudinal estimation sample (which includes only those panel firms for which bank loans are a relevant source of financing) using sample weights. Credit rationing rates are computed on the subsample of firms that have applied for a loan (for which Loan demand = 1). “*Credit rejected*” refers to those firms whose loan application was rejected; “*Strongly credit restricted*” indicate those firms that received below 75% of the amount requested and “*Weakly credit restricted*” those that received 75% and above. 2014H1 (Wave 11) refers to the period from April to September 2014, 2014H2 (Wave 12) refers to the period from October 2014 to March 2015 and so forth until 2019H1 (Wave 21), which refers to the period from April to September 2019

Figure 1 presents the patterns of the proportions of firms applying for a loan and of credit rationed firms over the sample period. We notice that the proportion of bank loan applications remained constant around 34% over the entire period, with a slight decrease only in the last survey wave. Conversely, we noticed that firms have benefitted from improved access to bank financing: the proportion of completely rejected applications has notably reduced since the beginning of 2014, and a similar decreasing pattern also characterizes the other two credit rationing indicators.

Table 2 reports transition probabilities of loan demand and rationing between *t*–1 and *t* (i.e. over a 6-month period). The cross tabulations show high percentages on the diagonal suggesting that loan demand and credit rationing tend to persist over time. Specifically, 76.86% of the firms that did not apply for a bank loan at *t*–1 did not enter the credit market at time *t*, whereas 58.32% of businesses that recurred to bank credit at time *t*–1 did the same at time *t*. Credit rationing is characterized by high persistence over time too: 96.08% of the firms whose applications were fully granted in *t*–1 remained in the initial state, while 52.17% of the firms that experienced a restriction in credit in *t*–1 were restricted again in *t*. Similar transition probabilities characterize the *Rationing 2* and *Rationing 3* indicators.

**Table 2:** Transition probabilities: persistence in loan demand and credit rationing

				*2) Credit rationing (if Loan demand* = 1*)*											
*1) Loan demand*				*2a) Rationing*				*2b) Rationing 2*				*2c) Rationing 3*			
	*D*_*t*_				*R*_*t*_				*R*_*t*_				*R*_*t*_		
		0	1			0	1			0	1			0	1
*D*_*t*-1_	0	76.86	23.14	*R*_*t*-1_	0	96.08	3.92	*R*_*t*-1_	0	91.99	8.01	*R*_*t*-1_	0	87.42	12.58
	1	41.68	58.32		1	47.83	52.17		1	49.60	50.40		1	46.13	53.87

Transition probabilities are expressed in percentage terms. *D*_*t*_ = 1 indicates that the firm applied for credit at time *t*, *R*_*t*_ = 1 indicates that the firm was credit rationed (according to the specific definition considered, namely, *Rationing*, *Rationing 2* or *Rationing 3*) at time *t*. Transition probabilities for credit rationing are computed for those firms that have applied for a loan

Loan demand and financing constraints are thus characterized by significant persistence over time, which can be due to observed firm characteristics, unobserved heterogeneity and true state dependence. One of the main aims of our analysis is to identify and consistently estimate true state dependence in access to credit, net of the other sources of persistence. In this respect, as pointed out by Pigini et al. (2016), the availability of longitudinal data from surveys carried out on a sub-annual frequency provides a better setting to disentangle the role of true state dependence in access to credit than surveys run on a longer basis. Despite costly application procedures and imperfect screening technologies may increase persistence in firms’ debt choices and banks’ credit granting decisions over shorter periods of time, a 6-month time frame may allow cleaner identification of state dependence by minimizing any contamination by events that took place over longer periods.^6^

### Control variables

To assess the main drivers of firms’ access to credit in euro area countries, we control for a large set of firm-level characteristics. First, we account for firm size and age as standard controls for information asymmetries. We include a set of binary variables for firm size, using the staff headcount criterion to distinguish *micro* (less than 10 employees), *small* (from 10 to 49 employees) *medium* (from 50 to 249 employees) and *large* (250 or more employees) firms. With respect to firm age, we consider dummies identifying firms younger than 5 years old, between 5 and less than 10 years old, and aged 10 years or older. Based on previous research (see Hyytinen & Pajarinen, 2008), we expect young and small businesses to be more likely to face credit restrictions due to their higher informational opacity. We also include a binary variable indicating whether the firm is an autonomous profit-oriented enterprise that makes independent financial decisions and a dummy equal to one if the firm’s owner is an individual, a family or a group of entrepreneurs.

As in Moro et al. (2017), we account for firm performances by means of two dummies identifying businesses reporting a decrease in turnover and profit over the last 6 months. Enterprises with a decreased ability to generate income are expected to have a higher propensity to demand bank credit, due to their reduced availability of internal funds, and a higher probability to face financing obstacles. Following Ferrando et al., (2017, 2019) and Cowling et al. (2020a), we also control for a firm’s own assessment of its collateral quality and past credit history. To this aim, we consider two dummies indicating whether the firm’s own capital and credit history deteriorated over the past 6 months. As banks use hard information in their credit granting decisions, we expect firms with an impaired quality of their fixed assets and with a reduced ability in serving their existing debts to be more likely to face financial constraints. As pointed out by Mascia and Rossi (2017), despite these dummies do not capture the actual dynamics of firm turnover, profits and own capital, they allow to control for the perceived changes in these balance sheet and income statement items from the perspective of the firm.

We also include a dummy indicating whether the firm experienced an improvement in the access to public financial support (including guarantees) during the last semester. As discussed in Mascia and Rossi (2017), the inclusion of this variable allows to control for the impact of government interventions designed to correct any failure or bias occurring in credit markets. According to previous research (Cowling, 2010; Cowling & Siepel, 2013), improved access to public financing, in the form of grants, loan guarantees and subsidized loans, is expected to ease financing constraints, reducing the barriers to additional finance and strengthening firm stability and future growth opportunities.

We account for the firm’s innovative activity by means of a binary variable indicating whether financing has been used for developing and launching new products and services. Due to the high return uncertainty, informational opacity, specificity and limited collateral value characterizing innovation projects, banks may be less likely to lend to innovative firms (Lee et al., 2015; Mateut, 2018). Small and young high-technology businesses are perceived as riskier by lenders and find greater difficulties in accessing credit, especially in the absence of public guarantee schemes (Cowling et al., 2018). We also include binary variables indicating whether the firm uses financing for investments in property, plants or equipment and for inventory and other working capital. These dummies allow to control, at least partially, for a firm’s strategic choice between short- and long-term financing and for investment tangibility. As banks may be more willing to grant credit to finance tangible and more collateralisable investments (Hall & Lerner, 2010), we expect a negative impact of the fixed investment dummy on the probability of facing credit restrictions.

In addition to firm-level factors, we also control for loan contract characteristics. Unfortunately, the SAFE survey provides information only on the size (in classes) of the last bank loan that the firm obtained or attempted to obtain in the last six months and does not allow to properly control for other price and non-price terms (i.e. interest rate, fees and commissions, maturity, collateral requirements, duration) of the loan contract.^7^ We thus assess whether the amount requested affects banks’ credit granting decisions by including binary controls for loan size.^8^ Despite applications for large loans are typical of larger and more creditworthy businesses, previous literature (see Kjenstad et al., 2015) suggests that, as loan size increases, lenders are less willing to grant the full amount at the market interest rate to reduce default risk.

We further extend the empirical specification to account for the role of country-level financial and institutional factors. As in Cowling et al. (2018), we use bank branch density (defined as the number of commercial bank branches per 100,000 adults) and the asset-based Herfindahl–Hirschman concentration index (HHI) to control for the level of financial development and for the degree of competition of the banking market. As discussed in Carbó-Valverde et al. (2009), the *market power* hypothesis suggests that less-developed and more concentrated credit markets are associated with lower credit availability. We also consider the share of credit cooperative institutions and the share of non-performing loans (NPLs) on total gross loans, as additional controls for the structure and health of the banking system. As discussed in Angelini et al. (1998), firms benefit form easier access to credit in systems with a larger share of cooperative banks, characterized by a local orientation and by a relationship-banking business model. Conversely, increased difficulties in accessing bank credit are expected in those countries characterized by a high level of NPLs (Mascia & Rossi, 2017). We also include the World Bank enforcing contracts score to assess whether the efficiency and quality of enforcement institutions affect bank lending relationships. Previous studies (Mora-Sanguinetti et al., 2017; Moro et al., 2018) have shown that better contract enforcement improves access to credit and alleviates financing constraints.

Finally, we control for sector-level heterogeneity by means of macro-sector dummies, while the effects of aggregate common shocks and residual heterogeneity at the country level are taken into account by means of survey wave and country-group dummies, respectively.^9^

Table 12 in the Appendix provides complete variable definitions, while Tables 13 and 14 report descriptive statistics and pairwise correlations for all explanatory variables, respectively.^10^

## Econometric methods

### *A static random-effects probit model with sample selection *

According to our definition of credit rationing, full or partial credit restrictions by banks can be observed only if the firm has actually applied for a loan. This may cause an endogenous selectivity bias arising from the non-random decision to apply for additional bank financing (Brown et al., 2011). To cope with this selectivity issue and account for time-invariant unobserved heterogeneity, we consider a random-effects probit model with sample selection; formally:

1 $$\begin{array}{c}{D}_{it}=1\left({{\boldsymbol{Z}}}_{it}^{^{\prime}}{\boldsymbol{\gamma}}+{\eta}_{i}+{\mu}_{it}>0\right)\\ {R}_{it}=1\left({{\boldsymbol{X}}}_{it}^{^{\prime}}{\boldsymbol{\beta}}+{\alpha }_{i}+{\varepsilon}_{it}>0\right)\end{array}$$

where $i=1,\dots ,N$, $t={0}_{i},\dots ,{T}_{i}$ and $1\left(\cdot \right)$ is an indicator function. The first equation models loan demand (*selection equation*) and rationing (${R}_{it}$) is observed only if ${D}_{it}=1$. ${{\boldsymbol{Z}}}_{it}$ and ${{\boldsymbol{X}}}_{{\boldsymbol{i}}t}$ are vectors of time-varying and time-invariant exogenous regressors and ${\boldsymbol{\gamma}}$ and ${\boldsymbol{\beta}}$ are the corresponding parameter vectors. To improve identifiability, we include only in the selection equation two dummies indicating whether (direct and indirect) production costs increased in the past 6 months and whether the firm expects a deterioration in the availability of external financing sources (other than bank loans) over the next 6 months. Coherently with Pigini et al. (2016) and Brown et al. (2011), perceptions on future access to other sources of finance and changes in production costs can be assumed to affect firm loan demand behaviour and financing strategies, but not to directly impact credit granting decisions by banks.

The individual specific error terms ${\eta}_{i}$ and ${\alpha }_{i}$ and the idiosyncratic errors ${\mu}_{it}$ and ${\varepsilon}_{it}$ are both assumed to follow a bivariate normal distribution with zero means and covariance matrices:

2 $${\Sigma }_{\eta \alpha }=\left(\begin{array}{cc}{\sigma }_{\eta }^{2}& {\rho }_{\eta \alpha }{\sigma }_{\eta }{\sigma }_{\alpha }\\ {\rho }_{\eta \alpha }{\sigma }_{\eta }{\sigma }_{\alpha }& {\sigma }_{\alpha }^{2}\end{array}\right), {\Sigma }_{\mu \varepsilon }=\left(\begin{array}{cc}1& {\rho }_{\mu \varepsilon }\\ {\rho }_{\mu \varepsilon }& 1\end{array}\right)$$

Equations in (1) are correlated through both the idiosyncratic errors (${\rho }_{\mu \varepsilon }$) and the individual effects (${\rho }_{\eta \alpha }$) and the “total” correlation can be computed as:

3 $${\rho }_{tot}=\frac{{\rho }_{\eta \alpha }{\sigma }_{\eta }{\sigma }_{\alpha }+{\rho }_{\mu \varepsilon }}{\sqrt{({\sigma }_{\eta }^{2}+1)({\sigma }_{\alpha }^{2}+1)}}$$

When ${\rho }_{tot}\ne 0$ (i.e. when ${\rho }_{\eta \alpha }\ne 0$ and/or ${\rho }_{\mu \varepsilon}\ne 0$) a sample selection issue arises and firms applying for credit cannot be considered as a random sample from the underlying population.

The individual likelihood function of the random-effects sample selection probit is given by:

4 $${L}_{i}=\underset{-\infty }{\overset{\infty }{\int }}\underset{-\infty }{\overset{\infty }{\int }}\left\{\prod_{t={0}_{i}}^{T}{L}_{it}\left({D}_{it},{R}_{it}|{{\boldsymbol{Z}}}_{i},{{\boldsymbol{X}}}_{i}, {\eta }_{i},{\alpha }_{i}\right)\right\}g\left({\eta }_{i},{\alpha }_{i}\right)d{\eta }_{i}d{\alpha }_{i}$$

where $\prod_{t={0}_{i}}^{T}{L}_{it}\left({D}_{it}{,R}_{it}|{{\boldsymbol{Z}}}_{i},{{\boldsymbol{X}}}_{i},{\eta}_{i},{\alpha }_{i}\right)$ is the likelihood function of individual *i* conditional on the individual effects and $g({\eta}_{i},{\alpha }_{i})$ is the bivariate normal density function of the individual effects. As discussed in Raymond et al. (2010), the double integral involved in the likelihood function (4) can be approximated by “two-step” Gauss-Hermite quadrature.

### Modelling the dynamics of loan demand and rationing

To control for the effects of past loan demand and financing constraints on current loan demand and rationing probabilities, we extend model (1) to a dynamic specification. Formally:

5 $$\begin{array}{c}{D}_{it}=1({\gamma}_{1}{D}_{it-1}+{\gamma}_{2}{R}_{it-1}^{*}+{{\boldsymbol{Z}}}_{it}^{^{\prime}}{{\boldsymbol{\gamma}}}_{3}+{\eta}_{i}+{\mu}_{it}>0) \\ {R}_{it}=1({\beta }_{1}{R}_{it-1}^{*}+{\beta }_{2}{D}_{it-1}+{{\boldsymbol{X}}}_{it}^{^{\prime}}{{\boldsymbol{\beta}}}_{3}+{\alpha }_{i}+{\varepsilon}_{it}>0)\end{array}$$

where ${D}_{it-1}$ and ${R}_{it-1}^{*}$ represent the lagged indicators of loan demand and rationing. Since the lagged credit rationing variable cannot be observed for those firms that did not apply in $t-1$, we follow Pigini et al. (2016) and recode missing values to zero: ${R}_{it-1}^{*}$ thus takes value 1 for firms that applied for credit and were rationed in $t-1$ and zero for firms which were not rejected or did not apply for credit in $t-1$.

Parameters ${\gamma}_{1}$ and ${\beta }_{1}$ in model (5) capture, respectively, the effect of past loan demand on current application probability and the impact of past credit restrictions on the conditional probability of being restricted in $t$. Positive and statistically significant estimates of ${\gamma}_{1}$ and ${\beta }_{1}$ identify persistence in loan demand and rationing probabilities, which may be due to true or spurious state dependence (Heckman, 1981). True state dependence arises when past loan demand and financing constraints have a genuine causal effect on future credit access conditions and significantly increase the probability of applying for a loan and of being rationed at time $t$, respectively. Spurious state dependence occurs instead when the intertemporal relationships are determined by time-invariant unobserved heterogeneity. To identify and consistently estimate true state dependence,^11^ it is necessary to account for unobserved effects that are correlated over time, as well as for the endogeneity of the initial conditions. Following Raymond et al. (2015), we extend Wooldridge’s (2005) approach to the initial conditions problem and model the distribution of the unobserved effects ${\eta}_{i}$ and ${\alpha }_{i}$ conditional on the initial value of the corresponding dependent variable and on exogenous regressors. Formally:

6 $${\eta}_i={b}_{10}+{b}_{11}{D}_{i{0}_i}+{\boldsymbol{Z}}_i^{+\prime }{\boldsymbol{b}}_{12}+{a}_{1i}$$

7 $${\alpha}_i={b}_{20}+{b}_{21}{R}_{i{0}_i}^{\ast }+{\boldsymbol{X}}_i^{+\prime }{\boldsymbol{b}}_{22}+{a}_{2i}$$

where ${D}_{i{0}_{i}}$ and ${R}_{{i0}_{i}}^{*}$ are the initial values of the dependent variables, ${{\boldsymbol{Z}}}_{{\boldsymbol{i}}}^{+}={({{\boldsymbol{Z}}}_{i{0}_{i+1}}^{{\prime}},\dots ,{{\boldsymbol{Z}}}_{i{T}_{i}}^{{\prime}})}^{\boldsymbol{^{\prime}}}$ and ${{\boldsymbol{X}}}_{{\boldsymbol{i}}}^{+}={({{\boldsymbol{X}}}_{i{0}_{i+1}}^{{\prime}},\dots ,{{\boldsymbol{X}}}_{i{T}_{i}}^{{\prime}})}^{{\prime}}$ represent the observed history of the exogenous regressors and ${a}_{1i}$ and ${a}_{2i}$ denote the projection errors assumed to be orthogonal to ${D}_{i{0}_{i}}$, ${R}_{i{0}_{i}}$, ${{\boldsymbol{Z}}}_{i}$, ${{\boldsymbol{X}}}_{i}$, ${\mu}_{it}$ and ${\varepsilon}_{it}$. The ancillary parameters ${b}_{10}$, ${b}_{11}$, ${{\boldsymbol{b}}}_{12}$, ${b}_{20}$, ${b}_{21}$ and ${{\boldsymbol{b}}}_{22}$ have to be estimated alongside the parameters in (5). Parameters ${\boldsymbol{\gamma}}_{3}$ and ${{\boldsymbol{b}}}_{12}$ and ${{\boldsymbol{\beta}}}_{3}$ and ${{\boldsymbol{b}}}_{22}$ cannot be separately identified if the explanatory variables are time-invariant or do not show sufficient within variation; for this reason, only sufficiently time-varying regressors are included in Eqs. (6) and (7). To reduce the number of parameters to be estimated, we substitute ${{\boldsymbol{Z}}}_{{\boldsymbol{i}}}^{+}$ and ${{\boldsymbol{X}}}_{{\boldsymbol{i}}}^{+}$ with the within-means ${\bar{{\boldsymbol{Z}}} }_{{\boldsymbol{i}}}^{+}=\frac{1}{{{\boldsymbol{T}}}_{{\boldsymbol{i}}}}{\sum }_{{\boldsymbol{t}}={1}_{{\boldsymbol{i}}}}^{{{\boldsymbol{T}}}_{{\boldsymbol{i}}}}{{\boldsymbol{Z}}}_{{\boldsymbol{i}}}^{+}$ and ${\bar{{\boldsymbol{X}}} }_{{\boldsymbol{i}}}^{+}=\frac{1}{{{\boldsymbol{T}}}_{{\boldsymbol{i}}}}{\sum }_{{\boldsymbol{t}}={1}_{{\boldsymbol{i}}}}^{{{\boldsymbol{T}}}_{{\boldsymbol{i}}}}{{\boldsymbol{X}}}_{{\boldsymbol{i}}}^{+}$, computed excluding the initial-period regressors ${{\boldsymbol{Z}}}_{{{\boldsymbol{i}}0}_{{\boldsymbol{i}}}}$ and ${{\boldsymbol{X}}}_{{{\boldsymbol{i}}0}_{{\boldsymbol{i}}}}$.^12^ The projection errors ${a}_{1i}$ and ${a}_{2i}$ and the idiosyncratic errors ${\mu}_{it}$ and ${\varepsilon }_{it}$ are both assumed to be bivariate normally distributed with zero means and covariance matrices:

8 $${\Sigma }_{{a}_{1}{a}_{2}}=\left(\begin{array}{cc}{\sigma }_{{a}_{1}}^{2}& {\rho }_{{a}_{1}{a}_{2}}{\sigma }_{{a}_{1}}{\sigma }_{{a}_{2}}\\ {\rho }_{{a}_{1}{a}_{2}}{\sigma }_{{a}_{1}}{\sigma }_{{a}_{2}}& {\sigma }_{{a}_{2}}^{2}\end{array}\right), {\Sigma }_{\mu \varepsilon }=\left(\begin{array}{cc}1& {\rho }_{\mu \varepsilon }\\ {\rho }_{\mu \varepsilon }& 1\end{array}\right)$$

The individual likelihood function of the dynamic random-effects sample selection probit takes the form:

9 $${L}_{i}=\underset{-\infty }{\overset{\infty }{\int }}\underset{-\infty }{\overset{\infty }{\int }}\left\{\prod_{t={0}_{i}+1}^{T}{L}_{it}\left({D}_{it},{R}_{it}|{D}_{i{0}_{i}},{D}_{it-1},{{\boldsymbol{Z}}}_{i}{,{R}_{{i0}_{i}}^{*},R}_{it-1}^{*},{{\boldsymbol{X}}}_{i},{a}_{1i},{a}_{2i}\right)\right\}g\left({a}_{1i},{a}_{2i}\right)d{a}_{1i}d{a}_{2i}$$

where the double integral can be evaluated using Gauss-Hermite quadrature sequentially, as in the static case. Using unbalanced panel data, we need at least three time observations for some of the firms, two of which must be consecutive, to identify the parameters of the lagged dependent variables in model (5) and those of the individual effects in Eqs. (6) and (7). As discussed in Raymond et al. (2010), the inclusion in the estimation sample of firms with only two consecutive observations, for which initial and lagged values of the dependent variables coincide, allows increasing the number of observations and does not affect identification.

### State dependence in access to credit, discouragement effect and past demand effect

Model (5) allows identifying state dependence in firms’ access to credit, as well as the effect of previous credit access difficulties on current loan demand behaviour and the effect of past demand behaviour on current rationing probability. As in Pigini et al. (2016), we evaluate the magnitude of state dependence and of cross-effects of lagged dependent variables in terms of average partial effects on loan demand and rationing probabilities at time $t$. Specifically, state dependence in loan demand is computed as the average difference between the probability of applying for a loan in $t$ conditional on having applied in $t-1$ and the probability of applying for a loan in $t$ conditional on not having applied in $t-1$:

10 $${\bar{D} }_{{D}_{t-1}}=\frac{1}{N}\sum_{i=1}^{N}\left[P\left({D}_{it}=1 \right| {D}_{it-1}=1)-P\left({D}_{it}=1 \right| {D}_{it-1}=0)\right]$$

State dependence in credit rationing is given by the average difference between the probability that ${R}_{it}$=1 conditional on having been rationed in $t-1$ and the probability that ${R}_{it}$=1 conditional on not having been rationed in $t-1$:

11 $${\bar{R} }_{{R}_{it-1}^{*}}=\frac{1}{N}\sum_{i=1}^{N}\left[{\mathrm{P}}\left({R}_{it}=1\right|{D}_{it}=1,{R}_{{it}-1}^{*}=1,{D}_{{it}-1}=1)\left.-{\mathrm{P}}\left({R}_{it}=1\right|{D}_{it}=1, \, {R}_{{it}-1}^{*}=0, \, {D}_{{it}-1}=1)\right]\right.$$

The effect of past restrictions on current loan demand (*discouragement effect*) is equal to the average difference between the probability of applying for a loan in $t$ conditional on having been rationed in $t-1$ and the probability of applying for a loan in $t$ conditional on not having been rationed in $t-1$:

12 $${\bar{D} }_{{R}_{it-1}^{*}}=\frac{1}{N}\sum_{i=1}^{N}\left[P\left({D}_{it}=1 \right| {R}_{it-1}^{*}=1, {D}_{it-1}=1)-P\left({D}_{it}=1\right| {R}_{it-1}^{*}=0, {D}_{it-1}=1)\right]$$

The effect of past demand on current rationing probability (*past demand effect*) is given by the average difference between the probability that ${R}_{it}$=1 conditional on having applied for a loan in $t-1$ and the probability that ${R}_{it}$=1 conditional on not having applied in $t-1$:

13 $${\bar{R} }_{{D}_{t-1}}=\frac{1}{N}\sum_{i=1}^{N}\left[P\left({R}_{it}=1\right|{D}_{it}=1, {D}_{it-1}=1)-P\left({R}_{it}=1\right|{D}_{it}=1, {D}_{it-1}=0)\right]$$

By explicitly assessing the role of past loan demand behaviour on current credit restrictions, we are able to test whether a repeated recourse to bank financing over time alleviates financial constraints, reducing information asymmetries through strong firm-bank relationships.

## Estimation results and discussion

### Modelling access to credit in a static setting

Table 3 presents estimation results for three alternative specifications of the static model (1): a baseline specification (Model *a)*), which focuses on the effects of firm-level characteristics, and two extended specifications (Models *b)* and *c)*) including loan size and credit market factors as additional controls. These models are estimated on an unbalanced sample of 21,385 firms observed for at least two (not necessarily consecutive) periods, for a total of 58,367 firm-wave observations.

**Table 3:** The determinants of firms’ access to credit—Static models

	*Model a)*				*Model b)*				*Model c)*			
	Rationing		Loan demand		Rationing		Loan demand		Rationing		Loan demand	
Micro	1.3959***	(0.1583)	− 0.6683***	(0.0488)	1.3677***	(0.1925)	-0.6684***	(0.0484)	1.3855***	(0.1845)	− 0.6925***	(0.0477)
Small	0.9522***	(0.1639)	− 0.3697***	(0.0478)	0.9324***	(0.1858)	-0.3696***	(0.0475)	0.9574***	(0.1778)	− 0.3852***	(0.0467)
Medium	0.5778***	(0.1689)	− 0.1888***	(0.0473)	0.5692***	(0.1807)	-0.1889***	(0.0471)	0.5684***	(0.1734)	− 0.1930***	(0.0463)
Autonomous firm	− 0.2125	(0.1301)	0.2908***	(0.0386)	− 0.2176*	(0.1139)	0.2908***	(0.0382)	− 0.2398**	(0.1101)	0.3007***	(0.0377)
Individual/family-owned	0.0871	(0.0931)	0.0150	(0.0328)	0.1235	(0.0952)	0.0150	(0.0325)	0.0961	(0.0919)	0.0330	(0.0320)
< 5 years	0.5027***	(0.1624)	− 0.0523	(0.0537)	0.5096***	(0.1397)	-0.0525	(0.0528)	0.4779***	(0.1344)	− 0.0446	(0.0519)
≥ 5 and < 10 years	0.0452	(0.1081)	0.0087	(0.0382)	0.0261	(0.1119)	0.0087	(0.0376)	0.0223	(0.1076)	0.0105	(0.0369)
Turnover decreased	0.1090	(0.0876)	− 0.0611*	(0.0327)	0.1189	(0.0903)	-0.0612*	(0.0324)	0.1222	(0.0869)	− 0.0664**	(0.0318)
Profit decreased	0.0959	(0.0877)	0.0863***	(0.0293)	0.1082	(0.0820)	0.0863***	(0.0289)	0.0845	(0.0790)	0.0894***	(0.0285)
Own capital deteriorated	0.4933***	(0.1384)	− 0.0291	(0.0405)	0.5023***	(0.0982)	-0.0292	(0.0393)	0.4247***	(0.0956)	− 0.0048	(0.0390)
Public support improved	− 0.6130***	(0.1894)	0.1071**	(0.0424)	− 0.6276***	(0.1624)	0.1072**	(0.0419)	− 0.5765***	(0.1558)	0.0908**	(0.0414)
Credit history deteriorated	0.5103**	(0.2356)	0.2067***	(0.0434)	0.5393***	(0.0943)	0.2064***	(0.0412)	0.5650***	(0.0905)	0.1913***	(0.0405)
Fixed investments	− 0.6664***	(0.1365)	0.6118***	(0.0239)	− 0.6537***	(0.0739)	0.6121***	(0.0237)	− 0.6651***	(0.0717)	0.6168***	(0.0234)
New products investment	0.3618***	(0.0828)	− 0.1318***	(0.0302)	0.3979***	(0.0789)	-0.1317***	(0.0296)	0.3642***	(0.0767)	− 0.1109***	(0.0293)
Working capital investment	0.0241	(0.0999)	0.0190	(0.0248)	0.0397	(0.0759)	0.0192	(0.0246)	0.0262	(0.0736)	0.0282	(0.0243)
Small loan					− 0.0814	(0.1047)			− 0.0973	(0.1006)		
Medium loan					0.0696	(0.1165)			0.0360	(0.1122)		
Medium-large loan					− 0.0052	(0.1226)			− 0.0516	(0.1183)		
Large loan					− 0.1494	(0.1487)			− 0.2013	(0.1452)		
Construction	0.1477	(0.1173)	− 0.1011**	(0.0435)	0.1957*	(0.1183)	-0.1010**	(0.0430)	0.1795	(0.1135)	− 0.0981**	(0.0423)
Trade	− 0.0538	(0.0956)	− 0.0310	(0.0344)	− 0.0302	(0.0991)	-0.0309	(0.0340)	− 0.0811	(0.0963)	− 0.0160	(0.0336)
Services	0.1516	(0.0947)	− 0.1229***	(0.0316)	0.1828*	(0.0940)	-0.1229***	(0.0313)	0.1799**	(0.0901)	− 0.1168***	(0.0309)
Branch density									− 0.0167***	(0.0043)	0.0146***	(0.0014)
HHI									0.9157	(0.6023)	1.0001***	(0.2178)
Cooperative banks									− 0.0007	(0.0030)	0.0047***	(0.0010)
NPL ratio									0.0121*	(0.0065)	0.0010	(0.0023)
Enforcing Contracts									− 0.0119**	(0.0057)	− 0.0045**	(0.0019)
Increased production costs			0.0143	(0.0230)			0.0132	(0.0230)			0.0065	(0.0226)
Pessimistic expectations			0.5443***	(0.0305)			0.5442***	(0.0296)			0.5390***	(0.0292)
Intercept	− 1.9018	(1.2257)	− 1.1910***	(0.0729)	− 1.9320***	(0.2852)	-1.1906***	(0.0724)	− 2.2238***	(0.5154)	− 1.5225***	(0.1637)
Time fixed-effects	Yes		Yes		Yes		Yes		Yes		Yes	
Country group fixed-effects	Yes		Yes		Yes		Yes		Yes		Yes	
*Random effects*												
${\sigma }_{\alpha }$	1.2317*** (0.1803)				1.2677*** (0.0516)				1.2295*** (0.0478)			
${\sigma }_{\eta }$	0.7672*** (0.0116)				0.7676*** (0.0115)				0.7503*** (0.0115)			
${\rho }_{\eta \alpha}$	− 0.2987* (0.1698)				− 0.2677*** (0.0505)				− 0.2796*** (0.0496)			
*Idiosyncratic errors*												
${\rho }_{\mu \varepsilon}$	− 0.5698*** (0.1317)				− 0.5467*** (0.0387)				− 0.5861*** (0.0365)			
*Total correlation (*${\rho }_{tot}$*)*	− 0.4261*** (0.1463)				− 0.3966*** (0.0166)				− 0.4260*** (0.0155)			
Number of observations	58,367				58,367				58,367			
Log-likelihood	− 35,355.91				− 35,173.31				− 35,025.73			

The Table reports the estimated coefficients of the static baseline random-effects model (Model *a)*) and of the extended static specifications including loan size in the credit rationing equation and controlling for credit market factors (Models *b)* and *c)*). Standard errors are reported in parentheses. *Large*, ≥ *10 years*, *Micro loan* and *Industry* are used as base levels for the categorical variables of firm size, firm age, loan size and sector, respectively

***, **, * denote significance at 1, 5 and 10% levels, respectively

From the lower part of Table 3, we notice that, in all the specifications, the standard deviations of the individual effects are highly significant and cross-equation correlation between ${\eta }_{i}$ and ${\alpha}_{i}$ is significant and negative, hinting at the presence of time-invariant unobservable factors that jointly affect credit demand and rationing probabilities.^13^ Accordingly, correlation between the idiosyncratic error terms (and consequently total error correlation) is negative and statistically significant. This evidence supports the necessity of accounting for endogenous selectivity and, as discussed in Brown et al. (2011) and Aristei and Gallo (2016), suggests that firms that are more likely to have a loan application rejected are also more likely to refrain from applying.

Baseline estimates (Model *a)* in Table 3) show that large companies are more likely to apply for bank financing and their loan applications are less likely to be turned down. Consistently with the extensive literature on firms’ financing constraints (Beck et al., 2006; Ferri et al, 2019), this evidence confirms that smaller businesses, due to their higher informational opacity and lower availability of collateral, face significantly higher difficulties in accessing bank credit. Conversely, autonomous profit-oriented firms, making independent financial decisions, seem to be perceived as more creditworthy by banks and face lower credit access issues. In line with the findings of Ferrando et al., (2017, 2019) and Cowling et al (2020a), we find that firms whose own capital and credit history have deteriorated during the last 6 months are characterized by a higher probability of being denied credit. These findings point out that a firm’s access to bank financing significantly depends on the quality of its collateral and on its ability in serving existing debts. As in Moro et al. (2017), perceived changes in turnover and profit affect firms’ loan demand behaviour, but they do not impact credit rationing probability. Specifically, firms reporting a reduction in turnover have a lower loan demand probability, whereas those reporting a decreased profitability are significantly more likely to need additional bank credit, due to their lower availability of internal financing sources. Furthermore, firms with improved access to public financial support are less likely to face difficulties in access to bank credit. This evidence confirms that public financial support measures significantly contribute to overcome information asymmetries and collateral constraints and to alleviate credit rationing, especially for small and opaque businesses. Coherently with Grundy and Verwijmeren (2020), the purpose for which financing is used significantly affects firms’ demand and access to bank credit, depending on the risk and collateral value of the investment. In particular, firms using financing for fixed investments are more likely to apply for bank loans, and, to the extent that tangible investments are more collateralisable, they are also less likely to be denied credit. An opposite evidence is obtained when financing is used for developing and launching new products or services: the higher riskiness and uncertainty of innovative investment projects reduce the probability of applying for a loan and increase the likelihood of credit rejection. Finally, firms in the services and construction sectors have a lower credit demand, whereas we find no significant sectoral heterogeneity in rationing probability.

When we extend the baseline specification to control for the effect of loan amount on credit availability (Model *b)* in Table 3), we find that none of the size dummies are statistically significant, pointing out that the size of financing does exert a significant effect on credit rejection probability. We also notice that the sign and statistical significance of the other regression parameters remain unchanged, supporting the robustness of our baseline results.

Results from the extended specification including credit market characteristics and institutional factors at the country-level (Model *c)* in Table 3) confirm the findings obtained in the previous two models. Moreover, we provide evidence that firms located in more financially developed countries benefit from improved access to bank credit: both the probabilities of applying for a bank loan and of having the application approved significantly increase with the number of bank branches per inhabitant. The degree of competition of the banking market and the presence of cooperative banks contribute to explain firms’ demand for bank financing but do not significantly affect the probability of obtaining credit. We also find that firms in countries with a higher NPL ratio are more likely to face financing constraints, suggesting that banks tend to adopt more conservative lending policies as their loan portfolio quality deteriorates. Finally, better contract enforcement reduces loan demand, possibly discouraging lower-quality firms, but significantly reduces credit rationing probability, coherently with Moro et al. (2018).

We have also re-estimated the extended model *c)* considering broader definitions of financing constraints, which account for both credit rejection and different degrees of quantity restrictions.^14^ Estimation results confirm the necessity of accounting for unobserved firm heterogeneity and endogenous selectivity and suggest that similar drivers affect a bank’s choice of denying or restricting credit. It is also worth remarking that when we consider rejected or strongly quantity constrained applications (i.e. the *Rationing 2* indicator), we find that firms applying for larger loans are less likely to be restricted than those applying for micro loans. This result may be due to the fact that applications for large financing are more likely to come from larger and more creditworthy businesses, which are potential users of non-lending products and services. Thus, in order to maintain relationships with valuable customers, banks may choose to restrict the amount of credit granted rather than to turn down their applications.

### The dynamics of access to credit

In this Section, we focus on the identification of state dependence in firms’ access to credit, which represents one of the main contributions of our study. Table 4 reports results for three alternative specifications of the dynamic model (5) (namely, Models *dyn_a)*, *dyn_b)* and *dyn_c)*), estimated on an unbalanced sample of 9597 firms observed for at least two consecutive periods (18,731 firm-wave observations).

**Table 4:** The determinants of firms’ access to credit—Dynamic models

	*Model dyn_a)*				*Model dyn_b)*				*Model dyn_c)*			
	Rationing		Loan demand		Rationing		Loan demand		Rationing		Loan demand	
${R}_{t-1}^{*}$	1.4479***	(0.1741)	− 0.0832	(0.0925)	1.4715***	(0.1667)	− 0.0905	(0.0914)	1.4212***	(0.1686)	− 0.0723	(0.0913)
${D}_{t-1}$	-0.3935***	(0.1021)	0.5329***	(0.0342)	− 0.3835***	(0.0769)	0.5333***	(0.0341)	− 0.3548***	(0.0784)	0.5232***	(0.0340)
Micro	0.6752***	(0.1623)	− 0.4151***	(0.0585)	0.5481***	(0.2017)	− 0.4148***	(0.0584)	0.5822***	(0.2032)	− 0.4508***	(0.0584)
Small	0.3652**	(0.1548)	− 0.2311***	(0.0558)	0.2565	(0.1880)	− 0.2307***	(0.0557)	0.3056	(0.1890)	− 0.2501***	(0.0557)
Medium	0.1207	(0.1615)	− 0.1086**	(0.0549)	− 0.0142	(0.1818)	− 0.1085**	(0.0549)	0.0014	(0.1833)	− 0.1114**	(0.0548)
Autonomous firm	-0.4205*	(0.2359)	0.1313	(0.1910)	− 0.5148**	(0.2439)	0.1289	(0.1899)	− 0.4872**	(0.2356)	0.1372	(0.1888)
Individual/family-owned	0.4316*	(0.2537)	0.1765	(0.1237)	0.4718*	(0.2669)	0.1763	(0.1233)	0.4742*	(0.2638)	0.1719	(0.1223)
< 5 years	-0.4091	(0.4830)	0.1213	(0.1866)	− 0.2461	(0.4943)	0.1212	(0.1865)	− 0.3352	(0.5098)	0.1052	(0.1857)
≥ 5 and < 10 years	-0.4125	(0.3164)	− 0.1072	(0.1157)	− 0.3477	(0.3273)	− 0.1075	(0.1162)	− 0.3813	(0.3261)	− 0.1044	(0.1157)
Turnover decreased	0.0690	(0.1312)	− 0.0626	(0.0552)	0.0198	(0.1338)	− 0.0625	(0.0551)	− 0.0273	(0.1372)	− 0.0587	(0.0548)
Profit decreased	0.1447	(0.1170)	0.0961**	(0.0484)	0.1782	(0.1204)	0.0962**	(0.0484)	− 0.1881	(0.1218)	0.0942*	(0.0482)
Own capital deteriorated	− 0.0494	(0.1589)	0.1002	(0.0748)	− 0.0911	(0.1589)	0.0999	(0.0747)	− 0.0915	(0.1597)	0.1032	(0.0746)
Public support improved	− 0.4377**	(0.2135)	0.0791	(0.0678)	− 0.4676**	(0.2201)	0.0795	(0.0678)	− 0.4840**	(0.2220)	0.0722	(0.0677)
Credit history deteriorated	0.2804*	(0.1467)	0.1072	(0.0735)	0.2522*	(0.1466)	0.1074	(0.0733)	0.2610*	(0.1482)	0.1094	(0.0729)
Fixed investments	− 0.4656***	(0.1179)	0.4154***	(0.0434)	− 0.4174***	(0.1129)	0.4151***	(0.0433)	− 0.4421***	(0.1156)	0.4184***	(0.0431)
New products investment	0.0511	(0.1307)	− 0.1332**	(0.0533)	0.0768	(0.1322)	-0.1329**	(0.0533)	0.0658	(0.1345)	− 0.1356**	(0.0532)
Working capital investment	0.0518	(0.1185)	− 0.2300***	(0.0455)	0.1131	(0.1194)	-0.2295***	(0.0454)	0.1026	(0.1213)	− 0.2290***	(0.0452)
Small loan					− 0.1231	(0.2562)			− 0.1308	(0.2581)		
Medium loan					− 0.0563	(0.2921)			− 0.0615	(0.2990)		
Medium-large loan					0.1155	(0.3145)			0.1254	(0.3226)		
Large loan					0.1698	(0.3708)			0.1906	(0.3835)		
Construction	0.1382	(0.1249)	− 0.0214	(0.0532)	0.1854	(0.1277)	− 0.0217	(0.0532)	0.1767	(0.1281)	− 0.0127	(0.0531)
Trade	− 0.0179	(0.0994)	− 0.0326	(0.0403)	0.0026	(0.1021)	− 0.0329	(0.0402)	− 0.0202	(0.1058)	− 0.0179	(0.0403)
Services	− 0.0068	(0.0967)	− 0.0738**	(0.0371)	0.0523	(0.0997)	− 0.0740**	(0.0370)	0.0415	(0.1004)	− 0.0609	(0.0371)
Branch density									− 0.0138**	(0.0058)	0.0153***	(0.0019)
HHI									1.4721*	(0.7602)	0.9406***	(0.2782)
Cooperative banks									− 0.0009	(0.0037)	0.0054***	(0.0013)
NPL ratio									− 0.0057	(0.0084)	0.0024	(0.0029)
Enforcing Contracts									− 0.0062	(0.0071)	− 0.0105***	(0.0025)
Increased production costs			0.0476*	(0.0282)			0.0469*	(0.0282)			0.0411*	(0.0242)
Pessimistic expectations			0.3586***	(0.0309)			0.3582***	(0.0309)			0.3635***	(0.0309)
Intercept	− 2.0101***	(0.3970)	− 1.3055***	(0.0903)	− 2.0852***	(0.3384)	− 1.3050***	(0.0903)	− 1.4256**	(0.6439)	− 1.2932***	(0.2020)
Time fixed-effects	Yes		Yes		Yes		Yes		Yes		Yes	
Country group fixed-effects	Yes		Yes		Yes		Yes		Yes		Yes	
*Initial conditions:*												
${R}_{{0}_{i}}^{*}$	0.5755***	(0.1838)			0.5976***	(0.1811)			0.6581***	(0.1821)		
${D}_{{0}_{i}}$			0.4035***	(0.0338)			0.4035***	(0.0338)			0.3904***	(0.0337)
*Random effects*												
${\sigma }_{a_{2}}$	0.5156*** (0.1210)				0.5364*** (0.0702)				0.5492*** (0.0669)			
${\sigma }_{a_{1}}$	0.4905*** (0.0206)				0.4905*** (0.0206)				0.4851*** (0.0205)			
${\rho }_{a_{1}a_{2}}$	− 0.1582 (0.2805)				− 0.1053 (0.1909)				− 0.1084 (0.1873)			
*Idiosyncratic errors*												
${\rho }_{\mu \varepsilon}$	− 0.2523*** (0.0955)				− 0.2782*** (0.0550)				− 0.2779*** (0.0554)			
*Total correlation (*${\rho }_{tot}$*)*	− 0.2333*** (0.0337)				− 0.2420*** (0.0340)				− 0.2407*** (0.0345)			
Number of observations	18,731				18,731				18,731			
Log-likelihood	− 11,156.58				− 11,101.58				− 11,035.50			

The Table reports the estimated coefficients of the dynamic baseline random-effects model (Model *dyn_a)*) and of the extended dynamic specifications including loan size in the credit rationing equation and controlling for credit market factors (Models *dyn_b)* and *dyn_c)*)*.* Standard errors are reported in parentheses. *Large*, ≥ *10 years*, *Micro loan* and *Industry* are used as base levels for the categorical variables of firm size, firm age, loan size and sector, respectively

***, **, * denote significance at 1, 5 and 10% levels, respectively

The lower part of Table 4 shows that, in all the dynamic specifications, the standard deviations of the unobserved individual effects in the two equations are significant at the 1% level, whereas their correlation is not statistically significant. However, the idiosyncratic error terms remain significantly and negatively correlated and the resulting total error correlation, despite being lower in absolute terms than that of the static models, is negative and statistically significant at the 1% level. This evidence confirms that sample selection needs to be accounted for, even in a dynamic setting. The coefficients of the initial values of loan demand and rationing indicators (${D}_{i{0}_{i}}$ and ${R}_{{i0}_{i}}^{*}$, respectively) are both highly significant, supporting the importance of handling the endogeneity of initial conditions to properly identify state dependence.

With respect to the effects of firm- and country-level regressors, results obtained from the dynamic models largely confirm the evidence obtained in the static specifications. The main differences relate to the effects of perceived changes in firm performance, risk and credit history, which are partially reduced when the intertemporal nature of firms’ access to bank financing is taken into account.^15^ In this latter respect, we find that current loan demand and rationing probabilities are significantly influenced by past credit access conditions. In all the specifications, the parameter of ${R}_{t-1}^{*}$ in the credit rationing equation is positive and statistically significant, highlighting that the current probability of being credit rationed is higher for firms that experienced a credit restriction in the previous period than for firms that were not restricted or did not apply for a loan in $t-1$. Accordingly, the sign and statistical significance of the parameter of ${D}_{t-1}$ in the selection equation provide evidence of strong state dependence in loan demand too. Past credit restrictions do not affect demand behaviour at time *t*, while the negative and statistically significant coefficient of the lagged loan demand indicator in the rationing equation suggests that repeated firm-bank interactions over time tend to alleviate financing constraints.

To assess the magnitude and significance of state dependence and of discouragement and past demand effects, in Table 5, we report the estimated average partial effects of lagged dependent variables on the probability of applying for credit and of being constrained for the three empirical specifications.

**Table 5:** State dependence in access to credit, discouragement and past demand effects

	*Model dyn_a)*	*Model dyn_b)*	*Model dyn_c)*
${\bar{D} }_{{D}_{t-1}}$* (state dependence in demand)*	0.1634***	0.1797***	0.1752***
	(.0101)	(0.0111)	(0.0111)
${\bar{R} }_{{R}_{t-1}}$* (state dependence in rationing)*	0.0900***	0.0736***	0.0697***
	(0.0275)	(0.0087)	(0.0086)
${\bar{D} }_{{R}_{t-1}}$* (discouragement effect)*	− 0.0255	− 0.0305	− 0.0241
	(0.0284)	(0.0308)	(0.0306)
${\bar{R} }_{{D}_{t-1}}$* (past demand effect)*	− 0.0177*	− 0.0141***	− 0.0125***
	(0.0105)	(0.0040)	(0.0039)

The Table reports the average partial effects of the lagged loan demand and rationing indicators on the predicted probabilities of applying for credit and of being constrained at time *t*. Standard errors are reported in parentheses

***, **, * denote significance at 1, 5 and 10% levels, respectively

On average, firms that were rationed in the previous semester are around 9% (Model *dyn_a)*) and 7% (Models *dyn_b)* and *dyn_c)*) more likely to be credit denied in *t* than those that were not restricted at time $t-1$. Two non-alternative mechanisms can explain the greater credit access difficulties of borrowers that were already restricted in the past. First, credit restrictions force firms to cut investment and prevent them exploiting new business opportunities, leading to a self-reinforcing deterioration in net worth that locks firms into a long-lasting credit trap. Second, in presence of screening technology frictions, the negative signalling of past credit restrictions increases banks’ propensity to keep their negative assessment on firm creditworthiness from one period to the other. At the same time, the use of credit scoring models allows banks to address resources towards borrowers with a better repayment history and thus firms that received a positive assessment in previous credit-worthiness tests will be advantaged with respect to restricted and new applicants.

For the whole sample of firms, differently from Pigini et al. (2016), we do not find evidence of any significant discouragement effect of past credit restrictions: the partial effect ${\stackrel{-}{D}}_{{R}_{{t}-{1}}}$ is negative, but not statistically significant in all the three specifications, suggesting that having experienced financing constraints in the past does not significantly reduce a firm’s current demand for bank loans.

We also point out the presence of significant state dependence in loan demand, in the extended model *dyn_c)*, firms that have already applied for credit in the past are 17.5% more likely to apply again in the next period. This result is not surprising, as bank credit is the main source of external financing for firms in the euro area and the recourse to market-based instruments is still limited. The evidence obtained also hints at the role of applications cost and limited access to collateral in determining state dependence in loan demand. As discussed in Kon and Storey (2003), under imperfect information and screening errors, firms (especially smaller ones) may incur high financial, in-kind and psychological application costs and high collateral requirements, which lead them not only to refrain from applying, but also to keep remaining out of the credit market over time. At the same time, firms having already applied for a loan in the past may be more likely to apply again in the future, as their reduced information opacity and improved ability to prepare applications lower application costs.

We find a positive and significative impact of past demand on the probability of being currently unconstrained: firms that applied for a bank loan in $t-1$ are between 1.77 and 1.25% (depending on the specification considered) less likely to be credit denied in $t$ than those firms that did not apply. This result is in line with the findings of Cole (1998) and Chakravarty and Yilmazer (2009), who point out that the probability of obtaining credit is higher for firms having pre-existing relationships with banks. A repeated recourse to bank financing over time is not simply indicative of a firm’s dependence on bank credit, but it may also denote the presence of strong firm-bank relationships and entail possible experience effects. Close and continuous lending interactions allow banks to accumulate knowledge on the firm and to form stronger relationships, reducing opacity issues and alleviating financial constraints. Furthermore, by repeatedly applying for credit, firms become familiar with application procedures and the experience gained over time not only reduces applications costs but also improves their efficiency in facing screening tests. As pointed out by Pigini et al. (2016), firms more dependent on bank credit and relying on relationship lending have higher incentives to disclose information and this reduces their likelihood to be locked in a credit restriction state, as banks are able to use updated soft information and may be readier to revise their assessments.

As in the static analysis, we re-estimate the dynamic specification *dyn_c)* using the two broader credit rationing indicators that also account for quantity constraints (i.e. *Rationing 2* and *Rationing 3*).^16^ Estimation results support the importance of addressing unobserved firm-level heterogeneity, sample selection bias and endogenous initial conditions and largely confirm the evidence obtained in the analysis of credit denial probability. To gauge the magnitude of state dependence and cross-effects of past loan demand and rationing, in Table 6 we report the average partial effects of the lagged dependent variables.

**Table 6:** State dependence in access to credit, discouragement and past demand effects using alternative credit rationing definitions

	*Rationing 2*	*Rationing 3*
${\bar{D} }_{{D}_{t-1}}$* (state dependence in demand)*	0.1738***	0.1717***
	(0.0112)	(0.0114)
${\bar{R} }_{{R}_{t-1}}$* (state dependence in rationing)*	0.0878***	0.1042***
	(0.0128)	(0.0163)
${\bar{D} }_{{R}_{t-1}}$* (discouragement effect)*	0.0011	0.0098
	(0.0224)	(0.0189)
${\bar{R} }_{{D}_{t-1}}$* (past demand effect)*	− 0.0151**	− 0.0117
	(0.0067)	(0.0098)

**T**he Table reports the average partial effects of the lagged loan demand and rationing indicators on the predicted probabilities of applying for credit and of being constrained at time *t*. Estimates are obtained from the dynamic random-effects model *dyn_c)* estimated using, alternatively, *Rationing 2* and *Rationing 3* as indicators of financing constraints. Standard errors are reported in parentheses

***, **, * denote significance at 1, 5 and 10% levels, respectively

Results highlight that state dependence in financial constraints is even more incisive when quantity restrictions are taken into account: the probability of experiencing a new restriction at time $t$ increases to 8.78 and 10.42% when we use the *Rationing 2* and *Rationing 3* indicators, respectively. The increased magnitude in the degree of state dependence with respect to the one reported in Table 5 suggests that constraints in firms’ access to credit mainly operate via quantity restrictions. Especially in periods of economic recovery, this form of rationing may represent a solution to avoid completely shutting down credit and interrupting lending relationships with firms facing temporary difficulties. The effect of past loan demand on the likelihood of being rationed remains statistically significant (at the 5% level) when we consider both credit denied and strongly quantity constrained firms, whereas it is not significant when weak quantity restrictions are included in the definition of financial constraints. The degree of state dependence in loan demand remains highly statistically significant, with a magnitude around 17%, while current loan demand behaviour is still unaffected by past credit restrictions.

### Firm size and the dynamics of access to credit

Table 7 shows average partial effects of past credit access conditions on loan demand and rationing probabilities in $t$, obtained from sub-samples defined by firm size group.^17^

**Table 7:** State dependence in access to credit, discouragement and past demand effects by firm size group

	*Rationing*	*Rationing 2*	*Rationing 3*
*Micro, small and medium enterprises*			
${\bar{D} }_{{D}_{t-1}}$* (state dependence in demand)*	0.1789 ***	0.1768***	0.1743***
	(0.0114)	(0.0116)	(0.0193)
${\bar{R} }_{{R}_{t-1}}$* (state dependence in rationing)*	0.0946***	0.0999***	0.1141***
	(0.0100)	(0.0148)	(0.0179)
${\bar{D} }_{{R}_{t-1}}$* (discouragement effect)*	− 0.0159	0.0093	0.0161
	(0.0302)	(0.0225)	(0.0193)
${\bar{R} }_{{D}_{t-1}}$* (past demand effect)*	− 0.0143***	− 0.0169**	− 0.0101
	(0.0049)	(0.0078)	(0.0109)
*Micro and small firms*			
${\bar{D} }_{{D}_{t-1}}$* (state dependence in demand)*	0.1710***	0.1684***	0.1660***
	(0.0141)	(0.0143)	(0.0146)
${\bar{R} }_{{R}_{t-1}}$* (state dependence in rationing)*	0.1228***	0.1233***	0.1353***
	(0.0139)	(0.0208)	(0.0237)
${\bar{D} }_{{R}_{t-1}}$* (discouragement effect)*	0.0174	0.0284	0.0279
	(0.0335)	(0.0259)	(0.0228)
${\bar{R} }_{{D}_{t-1}}$* (past demand effect)*	− 0.0186***	− 0.0261**	− 0.0228*
	(0.0072)	(0.0115)	(0.0134)
*Medium firms*			
${\bar{D} }_{{D}_{t-1}}$* (state dependence in demand)*	0.1950***	0.1942***	0.1919***
	(0.0185)	(0.0188)	(0.0193)
${\bar{R} }_{{R}_{t-1}}$* (state dependence in rationing)*	0.0720***	0.0906***	0.0942***
	(0.0132)	(0.0178)	(0.0240)
${\bar{D} }_{{R}_{t-1}}$* (discouragement effect)*	− 0.1424**	− 0.0421	− 0.0176
	(0.0623)	(0.0426)	(0.0344)
${\bar{R} }_{{D}_{t-1}}$* (past demand effect)*	− 0.0086*	− 0.0092	0.0062
	(0.0048)	(0.0098)	(0.0240)

The Table reports the average partial effects of the lagged loan demand and rationing indicators on the predicted probabilities of applying for credit and of being constrained at time *t*. Estimates are obtained from the dynamic random-effects model *dyn_c)* estimated on sub-samples defined by firm size group. Standard errors are reported in parentheses

***, **, * denote significance at 1, 5 and 10% levels, respectively

In all the sub-samples, we confirm the presence of significant state dependence in credit rationing, which increases as we progressively account for strong and weak quantity restrictions in the definition of rationing. For micro, small and medium enterprises, the effect ranges between 9.46 and 11.41%. The magnitude of the effect is even larger when we focus on the sub-sample of micro and small firms, increasing by about 3 percentage points on average. Differently from Pigini et al. (2016), we find that micro and small businesses are characterized by a higher degree of state dependence in credit restrictions than medium-sized enterprises. This result suggests that the adverse effects of past financial constraints on firms’ net-worth are exacerbated for small and opaque businesses, making them more likely to be locked in a credit restriction state and increasing their gap in accessing bank finance. In line with Ayyagari et al. (2018) and Fisera et al. (2019), the higher persistence in credit rationing of small businesses may also be related to the negative impact on access to finance of stricter bank capital requirements, which is particularly pronounced for small firms that have already experienced credit restrictions.

We also find significant state dependence in credit demand across sub-samples. The estimated partial effect of lagged demand on the current probability of applying for a bank loan is about 17.5% for micro, small and medium enterprises and is slightly more pronounced for medium-sized firms (about 19.5%). Interestingly, when we consider fully rejected applications, this group of firms is also characterized by a significant discouragement effect. In particular, medium-sized firms that were denied credit in $t-1$ are 14.24% more likely to refrain from applying for a bank loan at time *t*, as they anticipate a higher probability of credit denial. This evidence highlights that past credit rejection, being the strongest form of rationing, not only impairs firms’ investment opportunities and survival but also has a strong impact on their perceptions and self-initiative, curbing loan demand in the future. For the sub-sample of micro and small firms, we confirm the significant and negative effect of past loan demand on the current probability of facing credit restrictions. Micro and small firms are about 1.86–2.61% (depending on the credit rationing indicator considered) less likely to face restrictions in *t* when they have applied for bank financing also in the previous semester, whereas this effect tends to disappear for medium-sized enterprises. This result suggests that repeated lending interactions over time, by enhancing firms’ ability in dealing with application procedures and mitigating frictions in banks’ screening technologies, significantly improve access to credit for small and informationally opaque businesses.

## Robustness analyses

We conduct a number of additional analyses to assess the robustness of our main empirical findings.^18^

First, we re-estimate all the static random-effects specifications on the sub-sample of firms observed for at least two consecutive periods (i.e. on the estimation sample of the dynamic model). We obtain again high standard deviations of the random effects and high correlations between both the unobserved individual effects and the idiosyncratic errors of the two equations. This result suggests that the reduction in the estimated variance–covariance components of the individual effects that we observed moving from the static to the dynamic model is mainly due to the modelling of state dependence and does not depend on potential selection bias related to the restriction of the estimation sample.

Second, we delve into the role of firm riskiness in affecting access to credit. Despite the lack of income statement and balance sheet data, we follow Moro et al. (2017) and exploit the limited information available in the SAFE survey to construct a raw credit score. To this aim, we define four size classes (numbered from 0 = smallest to 3 = largest), three age classes (numbered from 0 = youngest to 2 = oldest), and three categorical variables capturing the perceived dynamics of firm turnover, profit and own capital (assuming values 0 = decreased, 1 = remain unchanged and 2 = increased). We then sum the values of these variables for each firm to obtain an index that ranges from 0 to 11. Higher values of this score indicate higher creditworthiness, based on the assumption that larger and older firms, as well as those reporting increased turnover and profits and improved own capital, are perceived as less risky by lenders. We re-estimate the static and dynamic models including this credit score as a control, simultaneously excluding its components from the set of regressors to avoid multicollinearity. Coherently with the results of previous studies investigating the effect of external risk ratings on access to credit (e.g. Cowling et al., 2020a, b), we find that firms characterized by a lower credit score have a significantly lower propensity to apply for a loan, as they may be more inclined to be credit discouraged, and are more likely face credit restrictions, being perceived as less creditworthy by banks. Furthermore, as it can be noticed from Table 8, the estimated average partial effects of lagged dependent variables on current loan demand and rationing probabilities remain completely unaffected by the inclusion of the credit risk measure.

**Table 8:** Robustness analysis: partial effects of lagged dependent variables controlling for a firm’s credit score

	*Rationing*	*Rationing 2*	*Rationing 3*
${\bar{D} }_{{D}_{t-1}}$* (state dependence in demand)*	0.1812***	0.1797***	0.1774***
	(0.0110)	(0.0112)	(0.0114)
${\bar{R} }_{{R}_{t-1}}$* (state dependence in rationing)*	0.0673***	0.0861***	0.1053***
	(0.0085)	(0.0128)	(0.0164)
${\bar{D} }_{{R}_{t-1}}$* (discouragement effect)*	− 0.0341	− 0.0054	0.0074
	(0.0306)	(0.0223)	(0.0188)
${\bar{R} }_{{D}_{t-1}}$* (past demand effect)*	− 0.0121***	− 0.0149**	− 0.0115
	(0.0038)	(0.0066)	(0.0099)

**T**he Table reports the average partial effects of the lagged loan demand and rationing indicators on the predicted probabilities of applying for credit and of being constrained at time *t*. Estimates are obtained from the dynamic random-effects model *dyn_c)* in which a raw credit score (based on the age and size of the firm and on changes in turnover and profit) is included as a control for firm creditworthiness, simultaneously excluding its components from the set of regressors. Standard errors are reported in parentheses

***, **, * denote significance at 1, 5 and 10% levels, respectively

As pointed out by Presbitero et al. (2014), a possible concern in the analysis of credit rationing probability is the inclusion in the estimation sample of those firms that do not demand additional credit as they do not need it. For this reason, as in García-Posada Gómez (2019) and Ferrando et al. (2017), we re-estimate the dynamic models excluding from the sample those firms that did not apply for a bank loan because they have sufficient internal funds. From the estimated average partial effects reported in Table 9, we notice that the degree of state dependence in loan demand remains highly statistically significant, even though its magnitude reduces to around 4.5%. This is an expected result, since firms with sufficient internal funds are more likely to keep remaining out of the credit market over time and excluding them from the estimation sample contributes to reduce the degree of state dependence in loan demand. State dependence in credit restriction preserves its magnitude and ranges between 7.85 and 11.87%. The effect of past loan demand on the current probability of financing constraints is still negative and statistically significant when we consider credit rejections and strong quantity restrictions together. Interestingly, we obtain evidence of a significant discouragement effect on the current demand behaviour of businesses that need additional credit. Firms that experienced a credit rejection in $t-1$ are 5.83% less likely to apply for a bank loan and this effect remains statistically significant at the 1% level also when strong and weak quantity restrictions are taken into account. This evidence suggests that, when we exclude those firms that are more likely to stay out of the credit market in any period, past credit constraints exert a significant discouragement effect on the current loan demand behaviour of those firms that actually need bank financing.

**Table 9:** Robustness analysis: partial effects of lagged dependent variables excluding firms that do not apply because of sufficient internal funds

	*Rationing*	*Rationing 2*	*Rationing 3*
${\bar{D} }_{{D}_{t-1}}$* (state dependence in demand)*	0.0415***	0.0475***	0.0466***
	(0.0073)	(0.0077)	(0.0079)
${\bar{R} }_{{R}_{t-1}}$* (state dependence in rationing)*	0.0785***	0.0988***	0.1187***
	(0.0095)	(0.0135)	(0.0171)
${\bar{D} }_{{R}_{t-1}}$* (discouragement effect)*	− 0.0583***	− 0.0570***	− 0.0429***
	(0.0138)	(0.0113)	(0.0105)
${\bar{R} }_{{D}_{t-1}}$* (past demand effect)*	− 0.0131***	− 0.0158**	− 0.0113
	(0.0045)	(0.0072)	(0.0105)

**T**he Table reports the average partial effects of the lagged loan demand and rationing indicators on the predicted probabilities of applying for credit and of being constrained at time *t*. Estimates are obtained from the dynamic random-effects model *dyn_c)* estimated on the subsample of firms needing external financing (i.e. excluding those firm that do not apply because of sufficient internal funds). Standard errors are reported in parentheses

***, **, * denote significance at 1, 5 and 10% levels, respectively

As in Raymond et al. (2010), we also assess the robustness of our results to the time dimension of the panel. To this aim, we estimate the dynamic model *dyn_c)* on the sub-sample of firms with at least four not necessarily consecutive observations over time (5737 firms for a for a total of 14,357 firm-wave observations) and on the subsample of firms with at least three consecutive observations over time (4767 firms, 13,466 firm-wave observations). It is firstly worth remarking that the variances of the individual effect, as well as the error correlation structures, are consistent with the ones obtained on the unbalanced sample of firms with at least two consecutive observations. Furthermore, the average partial effects reported in Table 10 show that state dependence in credit rationing is still significant across models, ranging between about 5.95 and 10.08% and 4.73 and 6.09% in the two subsamples, respectively. The increasing trend when strong and weak quantity restrictions are included in the definition of the credit rationing remains confirmed. Similarly, state dependence in loan demand is significant in all the models and around 17 and 16 percentage points in the two subsamples, respectively. Past demand behaviour exerts a negative and statistically significant effect on the current probability of being denied credit, still providing support to the beneficial effect of repeated lending interactions over time on access to credit.

**Table 10:** Robustness analysis: partial effects of lagged dependent variables for the sub-samples of firms with at least four not necessarily consecutive and three consecutive observations over time

	*Rationing*	*Rationing 2*	*Rationing 3*
*a) Subsample of firms with at least four not necessarily consecutive observations*			
${\bar{D} }_{{D}_{t-1}}$* (state dependence in demand)*	0.1706***	0.1689***	0.1670***
	(0.0123)	(0.0125)	(0.0127)
${\bar{R} }_{{R}_{t-1}}$* (state dependence in rationing)*	0.0595***	0.0804***	0.1008***
	(0.0087)	(0.0136)	(0.0177)
${\bar{D} }_{{R}_{t-1}}$* (discouragement effect)*	− 0.0007	0.0154	0.0191
	(0.0384)	(0.0278)	(0.0230)
${\bar{R} }_{{D}_{t-1}}$* (past demand effect)*	− 0.0122***	− 0.0111*	− 0.0125
	(0.0041)	(0.0063)	(0.0111)
*b) Subsample of firms with at least three consecutive observations*			
${\bar{D} }_{{D}_{t-1}}$* (state dependence in demand)*	0.1624***	0.1615***	0.1608***
	(0.0133)	(0.0135)	(0.0138)
${\bar{R} }_{{R}_{t-1}}$* (state dependence in rationing)*	0.0473***	0.0534***	0.0609***
	(0.0094)	(0.0148)	(0.0203)
${\bar{D} }_{{R}_{t-1}}$* (discouragement effect)*	− 0.0261	− 0.0065	− 0.0026
	(0.0435)	(0.0301)	(0.0250)
${\bar{R} }_{{D}_{t-1}}$* (past demand effect)*	− 0.0100***	− 0.0084	− 0.0013
	(0.0036)	(0.0074)	(0.0119)

The Table reports the average partial effects of the lagged loan demand and rationing indicators on the predicted probabilities of applying for credit and of being constrained at time *t*. Estimates are obtained from the dynamic random-effects model *dyn_c)* estimated on the subsample of firms with at least four not necessarily consecutive observations (panel *a)*) and the subsample of firms with at least three consecutive observations (panel *b)*). Standard errors are reported in parentheses

***, **, * denote significance at 1, 5 and 10% levels, respectively

Finally, we assess the appropriateness of the time frame considered to analyse the dynamics of access to bank credit and re-estimate all the dynamic models extending the length of the time frame from six months to one year. This robustness check requires focusing on those firms for which at least two paired observations over a 1-year period are available and reduces the size of the estimation sample to 8118 firms (for a total of 14,452 firm-wave observations). As it can be noticed from Table 11, the conclusions regarding the dynamics of access to credit still hold. The degree of state dependence in loan demand and credit rationing probabilities is still statistically significant at the 1% level, but it is lower than that estimated using a six-month time frame. This reduction is likely to be related to the increased role played by time-varying factors in shaping the overall persistence of loan demand behaviour and financing constraints over longer time frames. Consistently with our main empirical findings, we do not find evidence of credit discouragement, while the effect of past loan demand on the current credit rationing probability is still negative and statistically significant at the 10% when we consider credit denied and strongly quantity constrained applications.

**Table 11:** Robustness analysis: partial effects of lagged dependent variables with a 1-year time frame

	*Rationing*	*Rationing 2*	*Rationing 3*
${\bar{D} }_{{D}_{t-1}}$* (state dependence in demand)*	0.0959***	0.0939***	0.0930***
	(0.0152)	(0.0154)	(0.0156)
${\bar{R} }_{{R}_{t-1}}$* (state dependence in rationing)*	0.0359***	0.0572***	0.0951***
	(0.0109)	(0.0148)	(0.0182)
${\bar{D} }_{{R}_{t-1}}$* (discouragement effect)*	0.0393	0.0371	0.0367
	(0.0403)	(0.0342)	(0.0341)
${\bar{R} }_{{D}_{t-1}}$* (past demand effect)*	− 0.0108*	− 0.0112*	− 0.0061
	(0.0054)	(0.0061)	(0.0095)

**T**he Table reports the average partial effects of the lagged loan demand and rationing indicators on the predicted probabilities of applying for credit and of being constrained at time *t*. Estimates are obtained from the dynamic random-effects model *dyn_c)* estimated using a one-year time frame (instead of six-month time frame) for the lagged dependent variables. Standard errors are reported in parentheses

***, **, * denote significance at 1, 5 and 10% levels, respectively

## Conclusions

This study offers new insights on the determinants of firms’ access to credit in the euro area. Using detailed longitudinal survey data, we address the role of observed and unobserved heterogeneity and test for the degree of state dependence in credit demand and rationing probabilities. Both in a static and dynamic setting, firms’ access to credit is significantly affected by observed firm-level characteristics and country-level credit market factors. We also show that unobserved firm heterogeneity plays a crucial role in shaping differences in external financing conditions across firms and needs to be accounted for. Our empirical results are robust across the three indicators of financing constraints considered, suggesting that different types and intensities of financial constraints depend on similar drivers.

Most importantly, we highlight the need of accounting for the intertemporal nature of firms’ loan demand behaviour and banks’ credit granting choices. Controlling for observed and unobserved time-invariant individual heterogeneity and addressing sample selection bias, we provide strong empirical evidence significant state dependence in loan demand and rationing probabilities. In this respect, the high persistence in firms’ demand for bank financing is motivated by the limited recourse to capital markets and by the role of application costs. At the same time, constrained firms are more likely to be locked in a credit restriction state over time, due to the self-reinforcing deterioration of their net worth and to the negative signalling about their creditworthiness. Deepening the analysis by firm size, we show that state dependence in credit restriction is particularly strong for micro and small enterprises. We also document that past recourse to bank financing significantly reduces current rationing probability: repeated lending interactions over time improve firms’ ability in dealing with application procedures and allow banks to accumulate information, reducing screening-frictions and mitigating financing constraints. Furthermore, focusing on the subset of firms that actually need additional bank financing, we provide evidence of a significant discouragement effect of past credit restrictions on current loan application behaviour.

The significant degree of state dependence that characterize firms’ access to credit in the euro area provides support to the presence of substantial and persistent frictions in banking markets, which call for policy interventions and regulatory actions. In this sense, policies seeking to reduce information asymmetries between lenders and borrowers and to lower application costs, such as public initiatives aimed at supporting digitalisation and technology-enabled innovation in financial services and at providing firms with advice and information, may be particularly effective in alleviating financing constraints. Moreover, loan guarantee programmes, by reducing the default risk incurred by banks on corporate loans, represent essential instruments to ease access to credit and limit state dependence in financing constraints, especially for informationally opaque smaller and younger enterprises. At the same time, actions focused on financial sector stability through supply-side regulation, aimed at strengthening regulatory capital base and risk coverage, are necessary to further enhance the resilience of the banking system and limit systemic vulnerabilities.

The 2008–2009 global financial crisis has clearly shown that financial frictions play a key role in amplifying and increasing the persistence of adverse shocks. In this regard, the outbreak of the Covid-19 pandemic in 2020 has triggered sudden and unprecedented supply and demand shocks, which severely undermine global economic and financial stability. Despite the policy measures implemented by governments (mainly in the form of debt moratoriums, loan guarantee schemes and direct grants) have contributed to mitigate the negative short-run effects on corporate liquidity shortfall, the increased debt levels and the decreased profitability of businesses will likely result in a deterioration of their creditworthiness. The expected deterioration in the quality of credit may negatively affect financial stability and worsen financing constraints, which may in turn hinder economic recovery. Future research should investigate the long-run impact of the Covid-19 crisis on the financial and economic systems and assess the effectiveness of public policy measures to mitigate firms’ liquidity and solvency risks, reduce the persistence of credit restrictions and support economic recovery.

## Electronic supplementary material

Below is the link to the electronic supplementary material.

Supplementary file1 (PDF 882 KB)

## Appendix

**Table 12:** Variable definitions

Variable	Definition
*Dependent variables*	
Loan demand	Equal to 1 if the firm has applied for a bank loan in the past six months, 0 otherwise
Rationing	Equal to 1 if the firm applied for a bank loan, but its application was completely rejected, 0 otherwise
Rationing 2	Equal to 1 if the firm applied for a bank loan, but its application was completely rejected or it received below 75%, 0 otherwise
Rationing 3	Equal to 1 if the firm applied for a bank loan, but its application was completely rejected or it received below 75% or it receive 75% and above, 0 otherwise
*Firm-level explanatory variables*	
Micro	Equal to 1 if the firm has less than 10 employees, 0 otherwise
Small	Equal to 1 if the firm has 10 employees or more and less than 49, 0 otherwise
Medium	Equal to 1 if the firm has 49 employees or more and less than 249, 0 otherwise
Autonomous firm	Equal to 1 if the firm is an autonomous profit-oriented enterprise that makes independent financial decisions, 0 otherwise
Individual/family-owned	Equal to 1 if the firm is owned by an individual, a family or a group of entrepreneurs, 0 otherwise
< 5 years	Equal to 1 if the firm is younger than 5 years old, 0 otherwise
≥ 5 and < 10 years	Equal to 1 if the firm is between 5 and less than 10 years old, 0 otherwise
Turnover decreased	Equal to 1 if the firm's turnover has decreased over the past six months, 0 otherwise
Profit decreased	Equal to 1 if the firm's profit has decreased over the past six months, 0 otherwise
Own capital deteriorated	Equal to 1 if the firm's own capital has deteriorated over the past six months, 0 otherwise
Public support improved	Equal to 1 if the firm's access to public funds, including guarantees, improved over the past six months, 0 otherwise
Credit history deteriorated	Equal to 1 if the firm's credit history has deteriorated over the past six months, 0 otherwise
Fixed investment	Equal to 1 if the firm has used the financing for investments in property, plant or equipment during the past six months, 0 otherwise
New product investment	Equal to 1 if the firm has used the financing for developing or launching new products or services during the past six months, 0 otherwise
Working capital investment	Equal to 1 if the firm has used the financing for working capital investments, 0 otherwise
Small loan	Equal to 1 if the amount of firm's last bank loan requested or obtained was more than euro 25,000 and up to 100,000, 0 otherwise
Medium loan	Equal to 1 if the amount of firm's last bank loan requested or obtained was more than euro 100,000 and up to 250,000, 0 otherwise
Medium-large loan	Equal to 1 if the amount of firm's last bank loan requested or obtained was more than euro 250,000 and up to 1,000,000, 0 otherwise
Large loan	Equal to 1 if firm's last bank loan was more than 1,000,000 euro, 0 otherwise
*Country-level explanatory variables*	
Branch density	Commercial bank branches per 100,000 adults (Source: World Bank, World Development Indicators)
HHI	Herfindahl–Hirschman index for credit institutions (based on total assets) (Source: ECB Statistical Data Warehouse)
Cooperative banks	Co-operative banking sector share (Source: EACB, Key Statistics)
NPL ratio	Bank nonperforming loans to total gross loans (Source: ECB Statistical Data Warehouse)
Enforcing Contracts	Enforcing contracts score, on a scale from 0 (lowest) to 100 (highest) (Source: World Bank. Doing Business database)
*Identification variables*	
Increased production costs	Equal to 1 if the firm’s production costs increased over the past six months, 0 otherwise
Pessimistic expectations	Equal to 1 if the firm expects a deterioration in the availability of external financing sources, other than bank loans, over the next six months, 0 otherwise

**Table 13:** Descriptive statistics

	All firms (*N* = *58,367)*	Applying firms *(N* = *18,553)*	Unrestricted firms *(N* = *15,153)*	Credit denied firms *(N* = *1119)*	Quantity constrained firms *(N* = *2281)*
Micro	0.27	0.19	0.17	0.46	0.21
Small	0.22	0.20	0.19	0.27	0.22
Medium	0.17	0.19	0.20	0.13	0.18
Autonomous firm	0.84	0.84	0.84	0.89	0.83
Individual owner	0.75	0.73	0.72	0.85	0.74
< 5 years	0.04	0.03	0.03	0.08	0.04
≥ 5 and < 9 years	0.08	0.08	0.07	0.13	0.10
Turnover decreased	0.20	0.19	0.17	0.35	0.22
Profit decreased	0.30	0.31	0.29	0.50	0.39
Own capital deteriorated	0.10	0.10	0.08	0.30	0.15
Public support improved	0.08	0.09	0.09	0.03	0.09
Credit history deteriorated	0.09	0.10	0.08	0.33	0.16
Fixed investments	0.53	0.65	0.68	0.40	0.54
New products investment	0.21	0.20	0.19	0.24	0.22
Working capital investment	0.41	0.41	0.39	0.47	0.51
Small loan		0.18	0.17	0.29	0.21
Medium loan		0.13	0.13	0.20	0.16
Medium-large loan		0.21	0.20	0.20	0.23
Large loan		0.41	0.43	0.14	0.32
Branch density	32.04	34.83	33.84	36.74	41.53
HHI	0.07	0.07	0.07	0.08	0.08
Cooperative banks	27.76	29.62	30.32	29.88	24.22
NPL ratio	6.78	7.01	6.57	9.39	9.42
Enforcing Contracts	66.60	66.31	66.84	64.36	63.08

**Notes**: the Table reports means of the explanatory variables for the longitudinal estimation sample (which includes only those panel firms for which bank loans are a relevant source of financing) and for the sub-samples of panel firms having applied for a bank loan (i.e. *Loan demand* = 1), unrestricted firms (i.e. *Loan demand* = 1 and *Rationing 3* = 0), credit denied firms (i.e. *Loan demand* = 1 and *Rationing* = 1) and quantity constrained firms (i.e. *Loan demand* = 1, *Rationing* = 0 and *Rationing 3* = 1). Descriptive statistics are computed using sample weights

**Table 14:** Correlation matrix

		(1)	(2)	(3)	(4)	(5)	(6)	(7)	(8)	(9)	(10)	(11)	(12)
(1)	Micro	1.00											
(2)	Small	-0.35	1.00										
(3)	Medium	-0.30	-0.23	1.00									
(4)	Autonomous firm	0.29	0.13	-0.07	1.00								
(5)	Individual owner	0.31	0.14	-0.03	0.47	1.00							
(6)	< 5 years	0.10	0.00	-0.03	0.02	0.02	1.00						
(7)	≥ 5 and < 9 years	0.13	0.01	-0.04	0.03	0.04	-0.07	1.00					
(8)	Turnover decreased	0.11	-0.01	-0.04	0.02	0.02	-0.01	0.00	1.00				
(9)	Profit decreased	0.09	0.00	-0.03	0.03	0.04	-0.02	0.00	0.51	1.00			
(10)	Own capital deteriorated	0.10	-0.01	-0.04	0.03	0.03	0.02	0.03	0.27	0.31	1.00		
(11)	Public support improved	-0.03	0.00	0.01	0.00	0.00	0.00	0.00	-0.04	-0.05	-0.04	1.00	
(12)	Credit history deteriorated	0.06	0.00	-0.03	0.03	0.02	0.01	0.02	0.19	0.19	0.32	-0.02	1.00
(13)	Fixed investments	-0.23	-0.03	0.07	-0.05	-0.05	-0.02	-0.04	-0.10	-0.06	-0.05	0.03	-0.05
(14)	New products investment	-0.09	-0.02	0.01	-0.04	-0.04	0.00	0.00	-0.04	-0.03	-0.01	0.02	-0.01
(15)	Working capital investment	-0.10	-0.03	0.01	-0.03	-0.02	-0.01	-0.02	0.00	0.01	0.02	0.01	0.03
(16)	Small loan	0.32	0.20	-0.09	0.14	0.18	0.05	0.07	0.06	0.06	0.06	0.00	0.06
(17)	Medium loan	0.03	0.18	0.08	0.07	0.09	0.03	0.01	0.02	0.02	0.00	0.00	0.02
(18)	Medium-large loan	-0.14	0.03	0.21	-0.02	0.02	-0.03	-0.01	-0.02	0.00	-0.04	0.01	-0.03
(19)	Large loan	-0.37	-0.32	-0.10	-0.20	-0.29	-0.07	-0.10	-0.08	-0.09	-0.05	0.00	-0.07
(20)	Branch density	0.18	-0.03	-0.07	0.05	-0.02	0.00	0.02	0.09	0.08	0.01	0.07	0.07
(21)	HHI	0.08	-0.03	-0.02	-0.04	-0.02	0.01	0.02	0.04	0.04	0.03	0.05	0.00
(22)	Cooperative banks	-0.03	-0.01	-0.01	-0.10	-0.09	0.00	0.00	0.05	0.04	0.08	-0.04	0.06
(23)	NPL ratio	0.18	-0.02	-0.05	0.10	0.07	0.01	0.03	0.09	0.11	0.07	0.01	0.06
(24)	Enforcing Contracts	-0.18	0.01	0.05	-0.08	-0.06	0.00	-0.02	-0.07	-0.09	-0.03	-0.02	-0.05
		(13)	(14)	(15)	(16)	(17)	(18)	(19)	(20)	(21)	(22)	(23)	(24)
(13)	Fixed investments	1.00											
(14)	New products investment	0.14	1.00										
(15)	Working capital investment	-0.03	0.17	1.00									
(16)	Small loan	-0.09	-0.04	-0.02	1.00								
(17)	Medium loan	-0.04	-0.02	-0.01	-0.18	1.00							
(18)	Medium-large loan	0.01	-0.02	0.01	-0.23	-0.20	1.00						
(19)	Large loan	0.14	0.09	0.05	-0.38	-0.32	-0.42	1.00					
(20)	Branch density	-0.14	-0.10	-0.02	0.13	0.05	0.01	-0.21	1.00				
(21)	HHI	-0.12	-0.02	0.04	0.01	0.02	0.00	-0.03	0.04	1.00			
(22)	Cooperative banks	0.02	-0.06	-0.14	-0.01	-0.01	0.01	0.00	-0.04	-0.18	1.00		
(23)	NPL ratio	-0.11	-0.01	0.03	0.08	0.02	0.01	-0.11	0.37	0.28	-0.21	1.00	
(24)	Enforcing Contracts	0.09	-0.01	-0.06	-0.08	-0.02	0.01	0.09	-0.38	-0.23	0.20	-0.58	1.00

**Notes**: the Table reports pairwise Pearson correlation coefficients between the explanatory variables, computed on the longitudinal estimation sample (which includes only those panel firms for which bank loans are a relevant source of financing) using sample weights

## Footnotes

1 See Berger and Udell (2006) for a detailed classification of transactions-based and relationship-based lending technologies. Angori et al. (2019) and Ferri et al. (2019) provide evidence on the role of different lending technologies on credit availability in Europe and on the complementarities between transactional lending and soft information use.

2 For additional information on the SAFE survey and on the structure of the dataset see ECB (2020)

3 These indicators allow to directly capture financial constraints, avoiding the biases of traditional measures indirectly inferred from firms’ financial statements and of measures based on firms’ perception of access to finance as a pressing problem for their activities. For a discussion, see Farre-Mensa and Ljungqvuist (2015) and Ferrando and Mulier (2015)

4 It is worth remarking that in the SAFE questionnaire all the questions related to access to bank loans are asked only if the firm indicates bank loans as relevant for their activities (i.e. they had used in the past or considered using in the future). The estimation sample thus includes only firms for which bank loans are a relevant source of financing.

5 Cole and Sokolyk (2016) point out that discouraged firms are significantly different from credit denied firms on a number of dimensions and show that about one third of them would have received credit if they had applied for.

6 To delve into the appropriateness of the time frame considered to analyse the dynamics of firms’ access to credit, we have also computed transition probabilities considering a one-year time frame. Results, reported in Table S1 of the Supplementary Appendix, largely support the persistence over time of both loan demand and rationing probabilities, with only a slight decrease in the proportions of firms experiencing a credit restriction in both *t*–1 and *t.*

7 The SAFE provides information on perceived changes in the price and non-price terms and conditions of bank financing over the semester considered, which are not necessarily referred to the last loan the firm obtained or attempted to obtain in the last six months. This information captures instead the overall dynamics of firm financing conditions, which is likely to be simultaneously determined with the bank’s decision to grant or reject the loan application. The joint analysis of loan demand and rationing probabilities together with the dynamics of the price and non-price terms of bank financing would require a multivariate modelling approach that is beyond the scope of this study.

8 Specifically, we distinguish whether a firm applied for a m*icro loan* (up to 25,000 euro), a *small loan* (more than 25,000 and less than 100,000 euro), a *medium loan* (more than 100,000 and less than 250,000), a *Medium-large loan* (more than 250,000 and less than 1 million), a *large loan* (over 1 million).

9 Time and country-group fixed effects allow to partially control for the effects of monetary policy measures and of changes in banking regulation on firms’ access to credit. Ferrando et al. (2019) provide recent empirical evidence on the effectiveness of unconventional monetary policies in relieving firms’ financial constraints and stimulating economic activity. Ayyagari et al. (2018) and Fisera et al (2019) find that macroprudential regulation reduces access to finance especially for small and young firms, while De Jonghe et al. (2020) show that the impact of capital requirements on credit supply significantly varies with bank and firm characteristics, as well as with the monetary policy stance.

10 To gauge the potential impact of non-random attrition, in Table S2 of the Supplementary Appendix we report descriptive statistics for the entire sample including also non-panel firms (i.e. those enterprises observed only once). No significant differences in observed characteristics emerge between firms in the entire sample and those in the panel component of the SAFE, suggesting that attrition should not be a relevant issue in our analysis.

11 In the remainder of the paper, when the term *state dependence* is used without any further explanation, it is to be understood as referring to *true state dependence*.

12 Rabe-Hesketh and Skrondal (2013) show that the popular constrained version of Wooldridge’s (2005) auxiliary model, which includes within-means of time-varying regressors based on all periods including the first, is severely biased in short panels. Their simulation results indicate that this issue can be avoided by omitting the initial-period regressors from the within-means.

13 It is worth remarking that a comparison between static random-effects and pooled estimates reveals that the signs and significance levels of the regression coefficients do not significantly vary. As discussed in Bettin and Lucchetti (2016), the near-constancy in parameter estimates suggests that individual time-invariant factors ${\eta }_{i}$ and ${\alpha }_{i}$ are substantially orthogonal to observable factors and thus represent a distinct source of persistence. Complete estimation results of the pooled probit models with sample selection are reported in Table S3 of the Supplementary Appendix. In order to further assess the impact of non-random attrition, we have estimated the static pooled and random-effects models including in the estimation sample 14,220 non-panel firms (i.e. combining longitudinal and unpaired cross-sectional units). Results are reported in Table S4 of the Supplementary Appendix and largely confirm the estimates obtained in the longitudinal dataset, suggesting that our empirical findings are not driven by attrition bias.

14 Complete estimation results are reported in Table S5 of the Supplementary Appendix.

15 We have assessed the presence of possible feedback effects of past credit access conditions on variables capturing the perceived dynamics of firm performance, risk and credit history, by adapting the Wald test procedure proposed by Kalwij (2015). For the extended model *dyn_c)*, test statistics are equal to 3.75 (*p*-value = 0.5853) and 3.22 (*p*-value = 0.6655) in the loan demand and rationing equation, respectively, supporting the strict exogeneity of these regressors and the absence of feedback effects. Similar results hold for the other two specifications.

16 Complete estimation results are reported in Table S6 of the Supplementary Appendix.

17 Complete results, reported in Tables S7, S8 and S9 of the Supplementary Appendix, show that the estimated effects of the control variables, as well as the correlation structure of the error terms, are consistent across specifications.

18 Complete results of all the robustness analyses are reported in Tables from S10 to S16 of the Supplementary Appendix.
