# Supplementary material for: Heterogeneity and state dependence in firms’ access to bank credit
Source: Small Bus Econ (Dordr). 2021 Oct 21;59(1):47–78. doi: 10.1007/s11187-021-00545-x (PMC8530204; doi:10.1007/s11187-021-00545-x)
Supplement: Supplementary file 1 — Supplementary file1 (PDF 882 KB) [file 11187_2021_545_MOESM1_ESM.pdf]

Supplementary Appendix

Heterogeneity and state dependence in firms' access to bank credit

David Aristei and Gabriele Angori

Table S1 – Transition probabilities of loan demand and credit rationing considering a 1-year time frame

1) Loan demand				2) Credit rationing (if Loan demand = 1): 2a) Rationing				2b) Rationing 2				2c) Rationing 3		
	D_t				R_t				R_t				R_t	
	0	1			0	1			0	1			0	1
$D_{t\text{-}1}$ 0	74.50	25.50		$R_{t\text{-}1}$ 0	95.95	4.05		$R_{t\text{-}1}$ 0	91.95	8.05		$R_{t\text{-}1}$ 0	87.37	12.63
1	45.75	54.25		1	58.06	41.94		1	58.27	41.73		1	51.97	48.03

Notes: Transition probabilities are expressed in percentage terms. D_t=1 indicates that the firm applied for credit at time *t*, R_t=1 indicates that the firm was credit rationed (according to the specific definition considered, namely *Rationing*, *Rationing 2* or *Rationing 3*) at time *t*. Transition probabilities for credit rationing are computed for those firms that have applied for a loan.

Table S2 – Descriptive statistics for the sample including both panel and non-panel firms

	All firms (*N=72587*)	Applying firms (*N=22381*)	Unrestricted firms (*N=18181*)	Credit denied firms (*N=1428*)	Quantity constrained firms (*N=2772*)
Loan demand	0.34				
Rationing		0.05			
Rationing 2		0.10			
Rationing 3		0.16			
Micro	0.29	0.20	0.18	0.46	0.23
Small	0.21	0.20	0.19	0.27	0.21
Medium	0.17	0.19	0.19	0.13	0.17
Autonomous firm	0.83	0.84	0.83	0.9	0.84
Individual owner	0.74	0.71	0.71	0.83	0.74
<5 years	0.04	0.03	0.03	0.08	0.05
≥5 and <9 years	0.09	0.08	0.07	0.13	0.1
Turnover decreased	0.20	0.19	0.17	0.37	0.22
Profit decreased	0.30	0.31	0.29	0.51	0.4
Own capital deteriorated	0.10	0.10	0.09	0.32	0.15
Public support improved	0.07	0.08	0.08	0.03	0.09
Credit history deteriorated	0.09	0.10	0.08	0.34	0.16
Fixed investments	0.52	0.65	0.68	0.4	0.55
New products investment	0.21	0.20	0.19	0.24	0.24
Working capital investment	0.40	0.40	0.38	0.46	0.5
Small loan		0.18	0.17	0.29	0.21
Medium loan		0.13	0.13	0.19	0.15
Medium-large loan		0.20	0.2	0.19	0.23
Large loan		0.40	0.43	0.15	0.32
Branch density	31.83	34.47	33.47	37.19	40.81
HHI	0.07	0.07	0.07	0.09	0.08
Cooperative banks	27.71	29.63	30.38	28.87	24.36
NPL ratio	6.71	6.90	6.46	9.34	9.17
Enforcing Contracts	66.75	66.51	67.03	64.25	63.49

Notes: the Table reports means computed on the entire sample including both panel and non-panel firms for which bank loans are relevant and on the sub-samples of firms having applied for a bank loan (i.e., *Loan demand*=1), unrestricted firms (i.e., *Loan demand*=1 and *3*=0), credit denied firms (i.e., *Loan demand*=1 and *Rationing*=1) and quantity constrained firms (i.e., *Loan demand*=1, *Rationing*=0 and *Rationing 3*=1). Descriptive statistics are computed using sample weights.

Table S3 – Estimates of the static model of firms' access to credit using a pooled estimator

	Model a)				*Model b)*				*Model c)*			
	Rationing		Loan demand		Rationing		Loan demand		Rationing		Loan demand	
Micro	0.9684***	(0.0855)	-0.6230***	(0.0278)	0.9318***	(0.1020)	-0.6228***	(0.0278)	0.9669***	(0.0994)	-0.6385***	(0.0280)
Small	0.6772***	(0.0783)	-0.3571***	(0.0265)	0.6335***	(0.0945)	-0.3570***	(0.0265)	0.6633***	(0.0921)	-0.3661***	(0.0266)
Medium	0.3882***	(0.0802)	-0.1649***	(0.0260)	0.3454***	(0.0929)	-0.1649***	(0.0260)	0.3560***	(0.0913)	-0.1668***	(0.0260)
Autonomous firm	-0.2264***	(0.0738)	0.2544***	(0.0290)	-0.2219***	(0.0782)	0.2545***	(0.0290)	-0.2305***	(0.0775)	0.2617***	(0.0293)
Individual/family-owned	0.0527	(0.0574)	0.0466*	(0.0245)	0.0480	(0.0613)	0.0467*	(0.0245)	0.0344	(0.0603)	0.0613**	(0.0247)
<5 years	0.4105***	(0.0798)	-0.0932**	(0.0387)	0.4093***	(0.0827)	-0.0932**	(0.0387)	0.3950***	(0.0821)	-0.0882**	(0.0390)
≥5 and <10 years	0.1322*	(0.0699)	0.0564**	(0.0260)	0.0732	(0.0634)	0.0566**	(0.0260)	0.0691	(0.0629)	0.0564**	(0.0261)
Turnover decreased	0.1189**	(0.0543)	-0.0523**	(0.0242)	0.1101**	(0.0545)	-0.0524**	(0.0242)	0.1121**	(0.0542)	-0.0561**	(0.0243)
Profit decreased	0.0282	(0.0466)	0.0612***	(0.0214)	0.0381	(0.0474)	0.0614***	(0.0214)	0.0356	(0.0469)	0.0607***	(0.0216)
Own capital deteriorated	0.3166***	(0.0539)	0.0010	(0.0304)	0.3391***	(0.0556)	0.0011	(0.0304)	0.3028***	(0.0561)	0.0145	(0.0309)
Public support improved	-0.3913***	(0.1081)	0.1177***	(0.0302)	-0.4553***	(0.1075)	0.1178***	(0.0302)	-0.4444***	(0.1069)	0.1086***	(0.0305)
Credit history deteriorated	0.4139***	(0.0624)	0.1553***	(0.0296)	0.4028***	(0.0638)	0.1553***	(0.0296)	0.4215***	(0.0637)	0.1459***	(0.0298)
Fixed investments	-0.4630***	(0.0492)	0.4741***	(0.0175)	-0.4611***	(0.0501)	0.4742***	(0.0175)	-0.4769***	(0.0488)	0.4793***	(0.0177)
New products investment	0.2258***	(0.0450)	-0.1286***	(0.0223)	0.2436***	(0.0443)	-0.1285***	(0.0223)	0.2367***	(0.0442)	-0.1175***	(0.0225)
Working capital investment	0.0031	(0.0429)	-0.0116	(0.0186)	-0.0241	(0.0429)	-0.0115	(0.0186)	-0.0262	(0.0433)	-0.0040	(0.0187)
Small loan					-0.0625	(0.0578)			-0.0629	(0.0570)		
Medium loan					0.0375	(0.0644)			0.0244	(0.0636)		
Medium-large loan					0.0659	(0.0717)			0.0478	(0.0711)		
Large loan					-0.1594	(0.1008)			-0.1750*	(0.1003)		
Construction	0.0096	(0.0656)	-0.0525*	(0.0284)	0.0444	(0.0663)	-0.0524*	(0.0284)	0.0426	(0.0657)	-0.0569**	(0.0286)
Trade	-0.0791	(0.0538)	0.0252	(0.0223)	-0.0616	(0.0543)	0.0253	(0.0223)	-0.0885	(0.0542)	0.0309	(0.0224)
Services	0.0425	(0.0561)	-0.0539**	(0.0218)	0.0670	(0.0558)	-0.0539**	(0.0218)	0.0600	(0.0544)	-0.0503**	(0.0220)
Branch density									-0.0115***	(0.0031)	0.0091***	(0.0013)
HHI									1.5227***	(0.4018)	0.9473***	(0.1897)
Cooperative banks									-0.0012	(0.0020)	0.0035***	(0.0008)
NPL ratio									0.0052	(0.0041)	0.0023	(0.0017)
Bank financing relevance									-0.3118	(0.4503)	0.7406***	(0.1921)
Enforcing Contracts									-0.0109***	(0.0034)	-0.0046***	(0.0014)
Increased production costs			0.0487***	(0.0170)			0.0473***	(0.0170)			0.0401**	(0.0171)
Pessimistic expectations			0.5153***	(0.0217)			0.5154***	(0.0217)			0.5085***	(0.0218)
Intercept	-1.1996***	(0.2266)	-1.0182***	(0.0499)	-1.0083***	(0.2387)	-1.0180***	(0.0499)	-1.2948***	(0.3961)	-1.4782***	(0.1346)
Time fixed-effects	Yes		Yes		Yes		Yes		Yes		Yes	
Country group fixed-effects	Yes		Yes		Yes		Yes		Yes		Yes	
Idiosyncratic errors												
$\rho_{\varepsilon\mu}$	-0.4378***	(0.0861)			-0.4526***	(0.0859)			-0.4926***	(0.0830)		
Number of observations	58367				58367				58367			
Log-likelihood	-40240.87				-40010.72				-39851.68			

Notes: the Table reports the estimated coefficients of the baseline static pooled model (Model *a)*) and of the extended static pooled models, which enrich the baseline model to control for loan size (Model *b)*) and also for macroeconomic factors (Model *c)*). Standard errors are reported in parentheses. We use *Rationing* as indicator of financing constraints. *Large*, *≥10 years*, *Micro loan* and *Industry* are used as base levels for the categorical variables of firm size, firm age, loan size and sector, respectively.

***, **, * denote significance at 1, 5 and 10% levels, respectively.

Table S4 – Estimates of the static model of firms' access to credit on the sample including both panel and non-panel firms

	Model c) – Pooled				*Model c) – Random-effects*			
	Rationing		Loan demand		Rationing		Loan demand	
Micro	0.9650***	(0.0875)	-0.6432***	(0.0253)	1.3845***	(0.1396)	-0.7053***	(0.0387)
Small	0.6731***	(0.0811)	-0.3758***	(0.0241)	0.9459***	(0.1347)	-0.3978***	(0.0378)
Medium	0.3386***	(0.0778)	-0.1805***	(0.0236)	0.5391***	(0.1309)	-0.2066***	(0.0375)
Autonomous firm	-0.1775***	(0.0672)	0.2648***	(0.0261)	-0.2203***	(0.0852)	0.2710***	(0.0298)
Individual/family-owned	0.0055	(0.0523)	0.0487**	(0.0220)	0.0618	(0.0708)	0.0374	(0.0254)
<5 years	0.3121***	(0.0701)	-0.0647*	(0.0345)	0.3669***	(0.1018)	-0.0313	(0.0399)
≥5 and <10 years	0.0479	(0.0526)	0.0388*	(0.0233)	0.0356	(0.0792)	0.0222	(0.0293)
Turnover decreased	0.1173**	(0.0487)	-0.0590***	(0.0217)	0.1256*	(0.0672)	-0.0671***	(0.0257)
Profit decreased	0.0323	(0.0425)	0.0617***	(0.0193)	0.0675	(0.0612)	0.0853***	(0.0230)
Own capital deteriorated	0.3136***	(0.0518)	0.0100	(0.0270)	0.4207***	(0.0726)	-0.0059	(0.0309)
Public support improved	-0.4316***	(0.0973)	0.0916***	(0.0277)	-0.5924***	(0.1246)	0.0934***	(0.0342)
Credit history deteriorated	0.3910***	(0.0581)	0.1683***	(0.0262)	0.4845***	(0.0689)	0.2126***	(0.0317)
Fixed investments	-0.4851***	(0.0415)	0.4857***	(0.0158)	-0.6808***	(0.0546)	0.6254***	(0.0190)
New products investment	0.2180***	(0.0406)	-0.1148***	(0.0203)	0.3161***	(0.0595)	-0.1077***	(0.0236)
Working capital investment	-0.0188	(0.0383)	0.0067	(0.0168)	-0.0065	(0.0563)	0.0516***	(0.0197)
Small loan	-0.0542	(0.0494)			-0.0773	(0.0770)		
Medium loan	0.0433	(0.0559)			0.0412	(0.0869)		
Medium-large loan	0.0612	(0.0621)			-0.0123	(0.0913)		
Large loan	-0.1544*	(0.0869)			-0.1800	(0.1119)		
Construction	0.0738	(0.0575)	-0.0408	(0.0255)	0.1853**	(0.0890)	-0.0772**	(0.0344)
Trade	-0.0807*	(0.0484)	0.0170	(0.0204)	-0.0808	(0.0766)	-0.0216	(0.0279)
Services	0.1008**	(0.0478)	-0.0533***	(0.0199)	0.2217***	(0.0705)	-0.1162***	(0.0255)
Branch density	-0.0112***	(0.0023)	0.0112***	(0.0009)	-0.0154***	(0.0033)	0.0139***	(0.0011)
HHI	1.2781***	(0.3239)	0.3199**	(0.1472)	0.7148	(0.4520)	0.9279***	(0.1737)
Cooperative banks	-0.0027*	(0.0015)	0.0048***	(0.0006)	-0.0016	(0.0023)	0.0047***	(0.0008)
NPL ratio	0.0044	(0.0034)	0.0024	(0.0015)	0.0146***	(0.0050)	-0.0010	(0.0018)
Enforcing Contracts	-0.0105***	(0.0028)	-0.0046***	(0.0013)	-0.0136***	(0.0043)	-0.0057***	(0.0016)
Increased production costs			0.0457***	(0.0153)			0.0084	(0.0183)
Pessimistic expectations			0.5079***	(0.0199)			0.5515***	(0.0240)
Intercept	-1.2974***	(0.3064)	-1.1211***	(0.1046)	-1.9175***	(0.3929)	-1.3787***	(0.1312)
Time fixed-effects	Yes		Yes		Yes		Yes	
Country group fixed-effects	Yes		Yes		Yes		Yes	
Random effects								
σ_{α}						1.1553***	(0.0394)	
σ_{η}						0.7507***	(0.0115)	
$\rho_{\alpha\eta}$						-0.3749***	(0.0418)	
Idiosyncratic errors								
$\rho_{\varepsilon\mu}$		-0.5449***	(0.0694)			-0.7108***	(0.0300)	
Total correlation (ρ_{tot})						-0.5422***	(0.0140)	
Number of observations		72587				72587		
Log-likelihood		-49198.43				-43476.41		

Notes: the Table reports the estimated coefficients of the extended static pooled and random-effects models *c)*, including in the estimation sample also 14220 non-panel firms (i.e., combining longitudinal and unpaired cross-sectional units). Standard errors are reported in parentheses. *Large*, *≥10 years*, *Micro loan* and *Industry* are used as base levels for the categorical variables of firm size, firm age, loan size and sector, respectively.

***, **, * denote significance at 1, 5 and 10% levels, respectively.

Table S5 – Estimates of the static model of firms' access to credit using alternative credit rationing definitions

	Model c)				*Model c)*			
	Rationing 2		Loan demand		Rationing 3		Loan demand	
Micro	0.7511***	(0.1078)	-0.6906***	(0.0467)	0.5620***	(0.0899)	-0.6885***	(0.0465)
Small	0.4685***	(0.1018)	-0.3826***	(0.0457)	0.3464***	(0.0845)	-0.3811***	(0.0454)
Medium	0.2077**	(0.0960)	-0.1904***	(0.0454)	0.1555**	(0.0791)	-0.1886***	(0.0451)
Autonomous firm	-0.2536***	(0.0738)	0.2983***	(0.0371)	-0.2605***	(0.0635)	0.2977***	(0.0369)
Individual/family-owned	0.0605	(0.0611)	0.0368	(0.0314)	0.0830	(0.0533)	0.0370	(0.0313)
<5 years	0.2410***	(0.0916)	-0.0435	(0.0510)	0.2739***	(0.0841)	-0.0427	(0.0511)
≥5 and <10 years	0.0764	(0.0696)	0.0105	(0.0363)	0.1164*	(0.0614)	0.0097	(0.0361)
Turnover decreased	0.0476	(0.0568)	-0.0685**	(0.0312)	0.0331	(0.0508)	-0.0674**	(0.0312)
Profit decreased	0.0982*	(0.0509)	0.0893***	(0.0279)	0.0662	(0.0449)	0.0880***	(0.0279)
Own capital deteriorated	0.3313***	(0.0652)	-0.0052	(0.0383)	0.3569***	(0.0603)	-0.0057	(0.0383)
Public support improved	-0.3874***	(0.0887)	0.0925**	(0.0407)	-0.1790***	(0.0694)	0.0923**	(0.0404)
Credit history deteriorated	0.4111***	(0.0625)	0.1886***	(0.0397)	0.3326***	(0.0584)	0.1889***	(0.0397)
Fixed investments	-0.5758***	(0.0452)	0.6184***	(0.0230)	-0.5063***	(0.0402)	0.6188***	(0.0229)
New products investment	0.2065***	(0.0513)	-0.1115***	(0.0287)	0.2240***	(0.0462)	-0.1119***	(0.0287)
Working capital investment	0.0962**	(0.0463)	0.0302	(0.0239)	0.0876**	(0.0410)	0.0297	(0.0238)
Small loan	-0.0641	(0.0658)			0.0316	(0.0615)		
Medium loan	-0.0215	(0.0742)			0.0996	(0.0683)		
Medium-large loan	-0.1539*	(0.0786)			-0.0069	(0.0713)		
Large loan	-0.3531***	(0.0950)			-0.1174	(0.0821)		
Construction	0.0773	(0.0775)	-0.0980**	(0.0415)	0.1548**	(0.0694)	-0.0981**	(0.0415)
Trade	-0.0913	(0.0617)	-0.0158	(0.0329)	-0.0471	(0.0544)	-0.0161	(0.0329)
Services	0.1080*	(0.0562)	-0.1160***	(0.0302)	0.0984*	(0.0502)	-0.1166***	(0.0302)
Branch density	-0.0135***	(0.0032)	0.0128***	(0.0016)	-0.0130***	(0.0028)	0.0128***	(0.0016)
HHI	0.4270*	(0.2524)	0.7838***	(0.2320)	0.0765	(0.3907)	0.7749***	(0.2327)
Cooperative banks	-0.0066***	(0.0021)	0.0038***	(0.0011)	-0.0101***	(0.0018)	0.0038***	(0.0011)
NPL ratio	0.0070	(0.0044)	0.0005	(0.0023)	0.0035	(0.0039)	0.0004	(0.0022)
Enforcing Contracts	-0.0098***	(0.0037)	-0.0049**	(0.0019)	-0.0093***	(0.0032)	-0.0049***	(0.0019)
Increased production costs			0.0022	(0.0222)			0.0054	(0.0221)
Pessimistic expectations			0.5376***	(0.0286)			0.5384***	(0.0286)
Intercept	0.1935	(0.3816)	-1.7551***	(0.1843)	0.5382	(0.3338)	-1.7512***	(0.1839)
Time fixed-effects	Yes		Yes		Yes		Yes	
Country group fixed-effects	Yes		Yes		Yes		Yes	
Random effects								
σ_α	0.9196*** (0.0292)				0.8646*** (0.0247)			
σ_η	0.7519*** (0.0118)				0.7509*** (0.0119)			
$\rho_{\alpha\eta}$	-0.3450*** (0.0368)				-0.3170*** (0.0341)			
Idiosyncratic errors								
$\rho_{\varepsilon\mu}$	-0.7690*** (0.0187)				-0.7770*** (0.0156)			
Total correlation (ρ_{tot})	-0.5228*** (0.0183)				-0.5945*** (0.0151)			
Number of observations	58367				58367			
Log-likelihood	-37188.88				-39053.78			

Notes: the Table reports the estimated coefficients of the extended static model *c)*, using alternative definitions of credit rationing (*Rationing 2* and *Rationing 3*). Standard errors are reported in parentheses. *Large*, *≥10 years*, *Micro loan* and *Industry* are used as base levels for the categorical variables of firm size, firm age, loan size and sector, respectively.
***, **, * denote significance at 1, 5 and 10% levels, respectively.

Table S6 – Estimates of the dynamic model of firms' access to credit using alternative credit rationing definitions

	Model dyn c)				*Model dyn c)*			
	Rationing 2		Loan demand		Rationing 3		Loan demand	
R^*_{t-1}	0.9032***	(0.1322)	0.0003	(0.0669)	0.6882***	(0.1092)	0.0293	(0.0562)
D_{t-1}	-0.2244***	(0.0690)	0.5184***	(0.0344)	-0.1687***	(0.0651)	0.5122***	(0.0350)
Micro	0.0697	(0.1680)	-0.4527***	(0.0583)	0.1058	(0.1462)	-0.4536***	(0.0583)
Small	0.0005	(0.1499)	-0.2505***	(0.0556)	0.0409	(0.1319)	-0.2516***	(0.0556)
Medium	-0.1025	(0.1380)	-0.1110**	(0.0547)	-0.0295	(0.1205)	-0.1120**	(0.0547)
Autonomous firm	-0.2802	(0.3830)	0.1356	(0.1893)	-0.3292	(0.3339)	0.1393	(0.1884)
Individual/family-owned	0.4751*	(0.2781)	0.1711	(0.1224)	0.4880*	(0.2632)	0.1678	(0.1227)
<5 years	-0.0695	(0.4321)	0.1055	(0.1857)	0.2481	(0.3961)	0.1036	(0.1862)
≥5 and <10 years	-0.0575	(0.2870)	-0.1046	(0.1160)	0.0162	(0.2484)	-0.1054	(0.1160)
Turnover decreased	0.0675	(0.1231)	-0.0613	(0.0549)	0.0739	(0.1137)	-0.0613	(0.0549)
Profit decreased	0.0328	(0.1050)	0.0949**	(0.0482)	0.0458	(0.0981)	0.0949**	(0.0482)
Own capital deteriorated	0.2391**	(0.1219)	0.1024	(0.0747)	0.2856**	(0.1439)	0.1026	(0.0747)
Public support improved	-0.1879	(0.1712)	0.0727	(0.0678)	-0.2257	(0.1439)	0.0744	(0.0678)
Credit history deteriorated	0.3752***	(0.1365)	0.1083	(0.0730)	0.3118**	(0.1314)	0.1105	(0.0731)
Fixed investments	-0.2213**	(0.1020)	0.4184***	(0.0431)	-0.2139**	(0.0944)	0.4193***	(0.0431)
New products investment	-0.0972	(0.1196)	-0.1358**	(0.0531)	0.0070	(0.1104)	-0.1370***	(0.0531)
Working capital investment	0.0957	(0.1032)	-0.2303***	(0.0452)	0.0393	(0.0959)	-0.2302***	(0.0452)
Small loan	-0.2470	(0.2318)			-0.0189	(0.2400)		
Medium loan	-0.2007	(0.2773)			0.1007	(0.2755)		
Medium-large loan	-0.2441	(0.2990)			0.0528	(0.2936)		
Large loan	-0.2663	(0.3615)			0.0438	(0.3428)		
Construction	0.1553	(0.1156)	-0.0125	(0.0531)	0.1697	(0.1115)	-0.0127	(0.0531)
Trade	-0.0594	(0.0933)	-0.0163	(0.0403)	0.0625	(0.0852)	-0.0167	(0.0403)
Services	0.0278	(0.0844)	-0.0603	(0.0371)	0.0703	(0.0786)	-0.0609	(0.0371)
Branch density	-0.0083**	(0.0041)	0.0131***	(0.0021)	-0.0080**	(0.0040)	0.0131***	(0.0021)
HHI	0.7312*	(0.3892)	0.6225**	(0.3098)	0.0953	(0.7346)	0.6194**	(0.3094)
Cooperative banks	-0.0022	(0.0033)	0.0041***	(0.0014)	-0.0074**	(0.0031)	0.0041***	(0.0014)
NPL ratio	0.0012	(0.0069)	0.0016	(0.0029)	0.0019	(0.0063)	0.0016	(0.0029)
Enforcing Contracts	-0.0024	(0.0060)	-0.0110***	(0.0025)	-0.0060	(0.0053)	-0.0111***	(0.0025)
Increased production costs			0.0371*	(0.0222)			0.0366*	(0.0219)
Pessimistic expectations			0.3606***	(0.0309)			0.3625***	(0.0310)
Intercept	-0.9485	(0.5968)	-1.5122***	(0.2242)	-1.1381**	(0.5398)	-1.5132***	(0.2242)
Time fixed-effects	Yes		Yes		Yes		Yes	
Country group fixed-effects	Yes		Yes		Yes		Yes	
Initial conditions								
$R^*_{0_i}$	0.6435***	(0.1266)			0.7470***	(0.1025)		
D_{0_i}			0.3870***	(0.0337)			0.3877***	(0.0338)
Random effects								
σ_{a_2}		0.7094***	(0.0528)			0.7506***	(0.0474)	
σ_{a_1}		0.4853***	(0.0205)			0.4857***	(0.0205)	
$\rho_{a_1 a_2}$		-0.0983	(0.1214)			-0.0554	(0.1053)	
Idiosyncratic errors								
$\rho_{\varepsilon\mu}$		-0.1993***	(0.0466)			-0.2712***	(0.0415)	
Total correlation (ρ_{tot})		-0.1711***	(0.0275)			-0.2096***	(0.0325)	
Number of observations		18731				18731		
Log-likelihood		-11861.12				-12583.93		

Notes: the Table reports the estimated coefficients of the extended dynamic model *dyn_c)*, using alternative definitions of credit rationing (*Rationing 2* and *Rationing 3*). Standard errors are reported in parentheses. *Large*, *≥10 years*, *Micro loan* and *Industry* are used as base levels for the categorical variables of firm size, firm age, loan size and sector, respectively.

***, **, * denote significance at 1, 5 and 10% levels, respectively.

Table S7 – Estimates of the dynamic model of firms' access to credit on subsamples split by firm size (Rationing)

	SMEs				Micro-Small				Medium			
	Model dyn c)				*Model dyn c)*				*Model dyn c)*			
	Rationing		Loan demand		Rationing		Loan demand		Rationing		Loan demand	
R^*_{t-1}	1.4602***	(0.1522)	-0.0471	(0.0896)	1.3651***	(0.1563)	0.0514	(0.0989)	2.0140***	(0.3286)	-0.4323**	(0.1896)
D_{t-1}	-0.3124***	(0.0744)	0.5302***	(0.0348)	-0.2886***	(0.0827)	0.5052***	(0.0424)	-0.3344**	(0.1324)	0.5919***	(0.0588)
Micro	0.5223***	(0.1211)	-0.3331***	(0.0401)	0.2312***	(0.0872)	-0.1745***	(0.0359)				
Small	0.2830***	(0.0978)	-0.1356***	(0.0358)								
Autonomous firm	-0.4723*	(0.2656)	0.1011	(0.2065)	-0.2104	(0.3514)	0.0673	(0.3059)	-0.2110	(0.3366)	0.1383	(0.2742)
Individual/family-owned	0.5382**	(0.2690)	0.2209*	(0.1236)	1.0857***	(0.3899)	0.2141	(0.1630)	0.0441	(0.3304)	0.2088	(0.1844)
<5 years	-0.2203	(0.4670)	0.1077	(0.1807)	-0.2022	(0.5237)	-0.0193	(0.2004)	-0.4455	(0.5437)	0.4886	(0.3933)
≥5 and <10 years	-0.2855	(0.3050)	-0.1190	(0.1151)	-0.3219	(0.3369)	-0.1300	(0.1252)	-0.4017	(0.6424)	-0.1097	(0.2635)
Turnover decreased	0.0541	(0.1294)	-0.0314	(0.0556)	0.0976	(0.1409)	-0.0536	(0.0647)	0.0759	(0.2540)	0.0299	(0.1042)
Profit decreased	0.1375	(0.1156)	0.0968*	(0.0495)	0.0708	(0.1270)	0.0947	(0.0591)	0.3827*	(0.2203)	0.0792	(0.0864)
Own capital deteriorated	-0.1015	(0.1478)	0.0940	(0.0750)	-0.0347	(0.1596)	0.0415	(0.0853)	-0.4430	(0.2717)	0.2756*	(0.1508)
Public support improved	-0.5253**	(0.2144)	0.0887	(0.0689)	-0.5509**	(0.2229)	0.0983	(0.0818)	-0.5174	(0.4963)	0.0606	(0.1209)
Credit history deteriorated	0.1640	(0.1350)	0.1088	(0.0736)	0.0918	(0.1439)	0.1034	(0.0833)	0.2498	(0.2774)	0.1421	(0.1488)
Fixed investments	-0.3849***	(0.1098)	0.4440***	(0.0437)	-0.3583***	(0.1260)	0.4600***	(0.0515)	-0.5170***	(0.1950)	0.3991***	(0.0787)
New products investment	0.1459	(0.1244)	-0.1151**	(0.0549)	0.1238	(0.1417)	-0.1009	(0.0671)	0.2495	(0.2035)	-0.1282	(0.0909)
Working capital investment	0.0464	(0.1152)	-0.1854***	(0.0462)	0.0418	(0.1263)	-0.1096**	(0.0548)	-0.0371	(0.2243)	-0.3479***	(0.0818)
Small loan	-0.1106	(0.2310)			-0.0569	(0.2249)			-0.7280	(0.4744)		
Medium loan	-0.0328	(0.2713)			-0.0394	(0.2665)			-0.3513	(0.4908)		
Medium-large loan	0.0713	(0.2983)			0.0406	(0.3150)			-0.2316	(0.4489)		
Large loan	0.4154	(0.3497)			0.2513	(0.4977)			0.3516	(0.4853)		
Construction	0.1808	(0.1201)	-0.0072	(0.0523)	0.1424	(0.1375)	-0.0557	(0.0629)	0.3137	(0.2098)	0.0787	(0.0941)
Trade	0.0154	(0.0976)	-0.0159	(0.0395)	0.0687	(0.1082)	-0.0108	(0.0481)	-0.1716	(0.2463)	-0.0729	(0.0709)
Services	0.0929	(0.0931)	-0.0576	(0.0372)	0.0789	(0.1073)	-0.0776*	(0.0461)	0.2533	(0.1654)	-0.0441	(0.0626)
Branch density	-0.0092	(0.0062)	0.0130***	(0.0022)	-0.0092	(0.0068)	0.0113***	(0.0026)	-0.0045	(0.0135)	0.0139***	(0.0038)
HHI	1.7503**	(0.8796)	0.6319**	(0.3208)	1.4792*	(0.8653)	0.2331	(0.3920)	1.9347	(1.7720)	1.2876**	(0.5549)
Cooperative banks	0.0016	(0.0038)	0.0035**	(0.0014)	-0.0007	(0.0042)	0.0028*	(0.0017)	0.0135	(0.0092)	0.0040	(0.0025)
NPL ratio	-0.0057	(0.0082)	0.0010	(0.0029)	-0.0114	(0.0088)	-0.0008	(0.0035)	0.0226	(0.0179)	0.0054	(0.0054)
Enforcing Contracts	-0.0077	(0.0067)	-0.0116***	(0.0025)	-0.0085	(0.0072)	-0.0080***	(0.0030)	-0.0087	(0.0156)	-0.0200***	(0.0047)
Increased production costs			0.0203	(0.0288)			0.0046	(0.0343)			0.0528	(0.0517)
Pessimistic expectations			0.3291***	(0.0317)			0.2944***	(0.0376)			0.4081***	(0.0575)
Intercept	-0.9205	(0.6618)	-1.6447***	(0.2270)	-0.6119	(0.7409)	-1.8528***	(0.2780)	-1.6057	(1.3515)	-1.2542***	(0.3971)
Time fixed-effects	Yes		Yes		Yes		Yes		Yes		Yes	
Country group fixed-effects	Yes		Yes		Yes		Yes		Yes		Yes	
Initial conditions												
$R^*_{0_i}$	0.5942***	(0.1696)			0.5914***	(0.1724)			0.5909	(0.4420)		
D_{0_i}			0.3505***	(0.0345)			0.3252***	(0.0421)			0.3741***	(0.0583)
Random effects												
σ_{a_2}	0.3955***	(0.0797)			0.3059***	(0.1094)			0.2784***	(0.0915)		
σ_{a_1}	0.4605***	(0.0218)			0.4468***	(0.0266)			0.4598***	(0.0397)		
$\rho_{a_1 a_2}$	-0.2351	(0.2376)			-0.1602	(0.3545)			-0.2252	(0.1968)		
Idiosyncratic errors												
$\rho_{\varepsilon\mu}$	-0.2473***	(0.0540)			-0.2245***	(0.0600)			-0.2689***	(0.0651)		
Total correlation (ρ_{tot})	-0.2451***	(0.0462)			-0.2125***	(0.0563)			-0.2474***	(0.0596)		
Number of observations	16771				11536				5235			
Log-likelihood	-9890.97				-6762.251				-3059.00			

Notes: the Table reports the estimated coefficients of the extended dynamic model *dyn_c)*, estimated on subsamples split by firm size. Standard errors are reported in parentheses. *Medium, Large*, *≥10 years*, *Micro loan* and *Industry* are used as base levels for the categorical variables of firm size, firm age, loan size and sector, respectively.

***, **, * denote significance at 1, 5 and 10% levels, respectively.

Table S8 – Estimates of the dynamic model of firms' access to credit on subsamples split by firm size (Rationing 2)

	SMEs				Micro-Small				Medium			
	Model dyn c)				*Model dyn c)*				*Model dyn c)*			
	Rationing 2		Loan demand		Rationing 2		Loan demand		Rationing 2		Loan demand	
R^*_{t-1}	0.9095***	(0.1360)	0.0276	(0.0668)	0.9034***	(0.1562)	0.0839	(0.0766)	1.0993***	(0.2132)	-0.1276	(0.1292)
D_{t-1}	-0.2040***	(0.0714)	0.5237***	(0.0353)	-0.2417***	(0.0853)	0.4973***	(0.0431)	-0.1564	(0.1178)	0.5884***	(0.0597)
Micro	0.1900*	(0.1145)	-0.3350***	(0.0401)	0.0889	(0.0904)	-0.1746***	(0.0358)				
Small	0.1201	(0.0906)	-0.1366***	(0.0358)								
Autonomous firm	-0.1116	(0.4304)	0.1018	(0.2072)	-0.2448	(0.5601)	0.0687	(0.3066)	-0.0151	(0.5147)	0.1576	(0.2762)
Individual/family-owned	0.5230*	(0.3000)	0.2217*	(0.1237)	0.5264	(0.4170)	0.2120	(0.1624)	0.6116*	(0.3647)	0.2121	(0.1863)
<5 years	0.0587	(0.4491)	0.1093	(0.1809)	-0.0142	(0.5269)	-0.0179	(0.2002)	0.1775	(0.5332)	0.4939	(0.3970)
≥5 and <10 years	0.0162	(0.2953)	-0.1176	(0.1152)	-0.0185	(0.3270)	-0.1284	(0.1252)	0.1050	(0.5671)	-0.1070	(0.2667)
Turnover decreased	0.0445	(0.1269)	-0.0333	(0.0556)	0.1711	(0.1506)	-0.0552	(0.0646)	0.2685	(0.2005)	0.0247	(0.1047)
Profit decreased	-0.0343	(0.1104)	0.0969*	(0.0495)	0.0016	(0.1299)	0.0950	(0.0590)	0.0541	(0.1854)	0.0811	(0.0868)
Own capital deteriorated	0.0479	(0.1493)	0.0954	(0.0751)	0.1677	(0.1754)	0.0427	(0.0853)	-0.3228	(0.2469)	0.2800*	(0.1519)
Public support improved	-0.2453	(0.1803)	0.0877	(0.0688)	-0.3967*	(0.2260)	0.0986	(0.0816)	0.0747	(0.2620)	0.0598	(0.1214)
Credit history deteriorated	0.3169**	(0.1410)	0.1081	(0.0737)	0.2257	(0.1587)	0.1040	(0.0834)	0.5501**	(0.2550)	0.1402	(0.1487)
Fixed investments	-0.2292**	(0.1068)	0.4439***	(0.0437)	-0.3426***	(0.1306)	0.4596***	(0.0514)	0.0225	(0.1616)	0.4000***	(0.0790)
New products investment	-0.0503	(0.1242)	-0.1144**	(0.0549)	-0.1134	(0.1453)	-0.1008	(0.0669)	0.1349	(0.1919)	-0.1272	(0.0915)
Working capital investment	0.0242	(0.1064)	-0.1861***	(0.0462)	0.0731	(0.1234)	-0.1106**	(0.0546)	-0.0274	(0.1755)	-0.3475***	(0.0820)
Small loan	-0.2773	(0.2272)			-0.2181	(0.2360)			-0.5116	(0.4914)		
Medium loan	-0.2222	(0.2727)			-0.1727	(0.2952)			-0.3196	(0.4980)		
Medium-large loan	-0.2705	(0.2952)			-0.3221	(0.3317)			-0.2748	(0.5009)		
Large loan	0.0005	(0.3565)			-0.3306	(0.5276)			0.1471	(0.5306)		
Construction	0.1492	(0.1156)	-0.0088	(0.0523)	0.1157	(0.1434)	-0.0567	(0.0628)	0.1229	(0.1804)	0.0752	(0.0950)
Trade	-0.0491	(0.0934)	-0.0155	(0.0395)	-0.0779	(0.1106)	-0.0101	(0.0480)	0.0593	(0.1585)	-0.0717	(0.0711)
Services	0.0502	(0.0868)	-0.0587	(0.0371)	0.0138	(0.1067)	-0.0779*	(0.0460)	0.1368	(0.1377)	-0.0463	(0.0629)
Branch density	-0.0081	(0.0056)	0.0130***	(0.0022)	-0.0058	(0.0067)	0.0113***	(0.0026)	-0.0152*	(0.0088)	0.0139***	(0.0038)
HHI	1.6778*	(0.9351)	0.6288**	(0.3207)	1.5934*	(0.8873)	0.2360	(0.3909)	2.2381*	(1.2718)	1.2647**	(0.5579)
Cooperative banks	-0.0033	(0.0035)	0.0035**	(0.0014)	-0.0041	(0.0042)	0.0028*	(0.0017)	-0.0039	(0.0055)	0.0039	(0.0025)
NPL ratio	-0.0015	(0.0073)	0.0010	(0.0029)	-0.0140	(0.0088)	-0.0008	(0.0035)	0.0247**	(0.0112)	0.0049	(0.0054)
Enforcing Contracts	-0.0004	(0.0061)	-0.0116***	(0.0025)	-0.0057	(0.0071)	-0.0080***	(0.0030)	-0.0153	(0.0104)	-0.0200***	(0.0047)
Increased production costs			0.0190	(0.0288)			0.0033	(0.0342)			0.0495	(0.0520)
Pessimistic expectations			0.3269***	(0.0317)			0.2913***	(0.0375)			0.4079***	(0.0579)
Intercept	-1.0633*	(0.5999)	-1.6473***	(0.2270)	-0.0481	(0.7382)	-1.8576***	(0.2776)	-2.8092***	(1.0076)	-1.2479***	(0.3991)
Time fixed-effects	Yes		Yes		Yes		Yes		Yes		Yes	
Country group fixed-effects	Yes		Yes		Yes		Yes		Yes		Yes	
Initial conditions												
$R^*_{0_i}$	0.6556***	(0.1303)			0.6719***	(0.1483)			0.4625**	(0.2224)		
D_{0_i}			0.3480***	(0.0345)			0.3230***	(0.0421)			0.3710***	(0.0586)
Random effects												
σ_{a_2}	0.6977***	(0.0557)			0.6838***	(0.0698)			0.4823***	(0.1071)		
σ_{a_1}	0.4604***	(0.0218)			0.4449***	(0.0266)			0.4621***	(0.0398)		
$\rho_{a_1 a_2}$	-0.1833	(0.1297)			-0.1559	(0.1667)			-0.3129	(0.2919)		
Idiosyncratic errors												
$\rho_{\varepsilon\mu}$	-0.1420***	(0.0497)			-0.1412***	(0.0516)			-0.1325**	(0.0632)		
Total correlation (ρ_{tot})	-0.1497***	(0.0435)			-0.1423***	(0.0462)			-0.1653***	(0.0735)		
Number of observations	16771				11536				5235			
Log-likelihood	-10620.72				-7222.80				-3322.26			

Notes: the Table reports the estimated coefficients of the extended dynamic model *dyn_c)*, estimated on subsamples split by firm size. Standard errors are reported in parentheses. *Medium*, *Large*, *≥10 years*, *Micro loan* and *Industry* are used as base levels for the categorical variables of firm size, firm age, loan size and sector, respectively.

***, **, * denote significance at 1, 5 and 10% levels, respectively.

Table S9 – Estimates of the dynamic model of firms' access to credit on subsamples split by firm size (Rationing 3)

	SMEs				Micro-Small				Medium			
	Model dyn c)				*Model dyn c)*				*Model dyn c)*			
	Rationing 3		Loan demand		Rationing 3		Loan demand		Rationing 3		Loan demand	
R^{*}_{t-1}	0.6786***	(0.1086)	0.0476	(0.0572)	0.6919***	(0.1249)	0.0825	(0.0675)	0.7425***	(0.1897)	-0.0532	(0.1041)
D_{t-1}	-0.1326**	(0.0654)	0.5165***	(0.0359)	-0.1876**	(0.0773)	0.4914***	(0.0439)	-0.0034	(0.1168)	0.5816***	(0.0610)
Micro	0.1443	(0.0998)	-0.3352***	(0.0402)	0.0730	(0.0791)	-0.1743***	(0.0358)				
Small	0.0847	(0.0788)	-0.1365***	(0.0358)								
Autonomous firm	0.0212	(0.3468)	0.1034	(0.2070)	-0.0534	(0.4749)	0.0695	(0.3063)	0.1859	(0.4549)	0.1614	(0.2777)
Individual/family-owned	0.4017	(0.2649)	0.2197*	(0.1242)	0.4566	(0.3418)	0.2100	(0.1627)	0.3575	(0.3718)	0.2087	(0.1874)
<5 years	0.3327	(0.3928)	0.1073	(0.1818)	0.1962	(0.4551)	-0.0182	(0.2015)	0.9077	(0.5682)	0.4850	(0.3983)
≥5 and <10 years	0.0351	(0.2463)	-0.1189	(0.1155)	-0.0296	(0.2735)	-0.1288	(0.1255)	0.1995	(0.4979)	-0.1080	(0.2671)
Turnover decreased	0.0375	(0.1129)	-0.0337	(0.0557)	0.1053	(0.1303)	-0.0560	(0.0647)	0.1368	(0.2037)	0.0279	(0.1054)
Profit decreased	0.0523	(0.0984)	0.0965*	(0.0496)	0.0701	(0.1126)	0.0949	(0.0591)	0.0237	(0.1848)	0.0812	(0.0874)
Own capital deteriorated	0.2653*	(0.1406)	0.0952	(0.0753)	0.3692**	(0.1585)	0.0417	(0.0854)	0.0025	(0.2731)	0.2807*	(0.1530)
Public support improved	-0.2170	(0.1449)	0.0888	(0.0690)	-0.3781**	(0.1769)	0.1007	(0.0818)	0.1056	(0.2444)	0.0599	(0.1221)
Credit history deteriorated	0.2872**	(0.1308)	0.1106	(0.0739)	0.1973	(0.1443)	0.1067	(0.0836)	0.5850**	(0.2617)	0.1414	(0.1501)
Fixed investments	-0.1568*	(0.0945)	0.4444***	(0.0438)	-0.2200*	(0.1135)	0.4604***	(0.0515)	-0.0040	(0.1666)	0.4011***	(0.0795)
New products investment	0.0593	(0.1102)	-0.1160**	(0.0550)	-0.0468	(0.1318)	-0.1017	(0.0670)	0.2688	(0.1851)	-0.1285	(0.0922)
Working capital investment	0.0170	(0.0953)	-0.1866***	(0.0463)	0.0497	(0.1110)	-0.1103**	(0.0547)	0.0120	(0.1700)	-0.3488***	(0.0826)
Small loan	-0.0201	(0.2275)			0.0328	(0.2288)			-0.2560	(0.5768)		
Medium loan	0.1079	(0.2616)			0.1303	(0.2737)			0.1014	(0.5595)		
Medium-large loan	0.0477	(0.2804)			-0.0821	(0.3102)			0.1469	(0.5641)		
Large loan	0.1706	(0.3333)			0.1936	(0.4852)			0.2473	(0.5902)		
Construction	0.1594	(0.1072)	-0.0092	(0.0524)	0.1146	(0.1281)	-0.0565	(0.0629)	0.1720	(0.1951)	0.0759	(0.0955)
Trade	0.0552	(0.0820)	-0.0158	(0.0396)	0.0241	(0.0966)	-0.0106	(0.0481)	0.0757	(0.1528)	-0.0733	(0.0716)
Services	0.0770	(0.0774)	-0.0589	(0.0372)	-0.0315	(0.0940)	-0.0782*	(0.0461)	0.3457***	(0.1307)	-0.0470	(0.0632)
Branch density	-0.0108**	(0.0049)	0.0130***	(0.0022)	-0.0063	(0.0058)	0.0113***	(0.0026)	-0.0216**	(0.0085)	0.0139***	(0.0038)
HHI	0.3722	(0.7506)	0.6284*	(0.3208)	0.6722	(0.9021)	0.2381	(0.3913)	3.4759***	(1.2929)	1.2699**	(0.5603)
Cooperative banks	-0.0090***	(0.0031)	0.0035**	(0.0014)	-0.0075**	(0.0037)	0.0029*	(0.0017)	-0.0129**	(0.0055)	0.0039	(0.0026)
NPL ratio	-0.0012	(0.0064)	0.0010	(0.0029)	-0.0119	(0.0075)	-0.0008	(0.0035)	0.0210*	(0.0111)	0.0049	(0.0054)
Enforcing Contracts	-0.0062	(0.0054)	-0.0117***	(0.0025)	-0.0006	(0.0062)	-0.0081***	(0.0030)	-0.0221**	(0.0098)	-0.0201***	(0.0048)
Increased production costs			0.0197	(0.0289)			0.0036	(0.0343)			0.0510	(0.0523)
Pessimistic expectations			0.3284***	(0.0318)			0.2950***	(0.0376)			0.4051***	(0.0583)
Intercept	-1.2677**	(0.5345)	-1.6483***	(0.2273)	-0.8158	(0.6420)	-1.8610***	(0.2779)	-2.3534**	(0.9778)	-1.2488***	(0.4013)
Time fixed-effects	Yes		Yes		Yes		Yes		Yes		Yes	
Country group fixed-effects	Yes		Yes		Yes		Yes		Yes		Yes	
Initial conditions												
$R^{*}_{0_i}$	0.6992***	(0.1029)			0.6770***	(0.1177)			0.7004***	(0.1860)		
D_{0_i}			0.3505***	(0.0346)			0.3251***	(0.0422)			0.3773***	(0.0590)
Random effects												
σ_{a_2}	0.6976***	(0.0498)			0.6423***	(0.0648)			0.6846***	(0.0829)		
σ_{a_1}	0.4618***	(0.0218)			0.4459***	(0.0266)			0.4676***	(0.0397)		
$\rho_{a_1 a_2}$	-0.0905	(0.1174)			-0.0539	(0.1578)			-0.1148	(0.2114)		
Idiosyncratic errors												
$\rho_{\varepsilon\mu}$	-0.2106***	(0.0439)			-0.2046***	(0.0512)			-0.1560**	(0.0789)		
Total correlation (ρ_{tot})	-0.1785***	(0.0369)			-0.1691***	(0.0461)			-0.1441**	(0.0607)		
Number of observations	16771				11536				5235			
Log-likelihood	-11254.22				-7575.78				-3594.81			

Notes: the Table reports the estimated coefficients of the extended dynamic model *dyn_c)*, estimated on subsamples split by firm size. Standard errors are reported in parentheses. *Medium, Large*, *≥10 years*, *Micro loan* and *Industry* are used as base levels for the categorical variables of firm size, firm age, loan size and sector, respectively.
***, **, * denote significance at 1, 5 and 10% levels, respectively.

Table S10 – Robustness analysis: estimates of the static model of firms' access to credit on the sub-sample used for the dynamic model

	Model c)		*Model c)*		*Model c)*	
	Rationing	Loan demand	Rationing 2	Loan demand	Rationing 3	Loan demand
Micro	1.3877*** (0.2505)	-0.7896*** (0.0765)	0.6082*** (0.1742)	-0.7864*** (0.0756)	0.4882*** (0.1415)	-0.7835*** (0.0755)
Small	0.8463*** (0.2426)	-0.4526*** (0.0743)	0.3467** (0.1611)	-0.4492*** (0.0731)	0.2826** (0.1311)	-0.4474*** (0.0730)
Medium	0.3452 (0.2433)	-0.2106*** (0.0739)	0.0699 (0.1534)	-0.2092*** (0.0727)	0.0505 (0.1223)	-0.2081*** (0.0725)
Autonomous firm	-0.1583 (0.1653)	0.2168*** (0.0618)	-0.2670** (0.1221)	0.2181*** (0.0612)	-0.2710*** (0.1029)	0.2189*** (0.0609)
Individual/family-owned	0.1586 (0.1377)	0.0341 (0.0527)	0.1622 (0.1038)	0.0347 (0.0520)	0.0890 (0.0882)	0.0353 (0.0519)
<5 years	0.2981 (0.2196)	-0.0801 (0.0886)	0.2160 (0.1730)	-0.0824 (0.0878)	0.3474** (0.1507)	-0.0814 (0.0884)
≥5 and <10 years	-0.2017 (0.1844)	-0.0747 (0.0630)	-0.0266 (0.1327)	-0.0741 (0.0624)	0.0628 (0.1114)	-0.0734 (0.0621)
Turnover decreased	0.1603 (0.1322)	-0.1146** (0.0517)	0.1264 (0.0976)	-0.1172** (0.0512)	0.0634 (0.0851)	-0.1189** (0.0515)
Profit decreased	0.0684 (0.1239)	0.1146** (0.0460)	0.0283 (0.0877)	0.1121** (0.0454)	0.0288 (0.0746)	0.1131** (0.0456)
Own capital deteriorated	0.3052** (0.1487)	0.0743 (0.0636)	0.2634** (0.1124)	0.0741 (0.0636)	0.3755*** (0.0992)	0.0734 (0.0638)
Public support improved	-0.6268** (0.2551)	0.0731 (0.0661)	-0.3336** (0.1508)	0.0714 (0.0649)	-0.2318** (0.1174)	0.0715 (0.0649)
Credit history deteriorated	0.6125*** (0.1372)	0.1362** (0.0655)	0.6161*** (0.1049)	0.1360** (0.0653)	0.5019*** (0.0955)	0.1396** (0.0660)
Fixed investments	-0.7047*** (0.1089)	0.5740*** (0.0388)	-0.5604*** (0.0773)	0.5725*** (0.0384)	-0.4860*** (0.0668)	0.5746*** (0.0385)
New products investment	0.3112*** (0.1162)	-0.0956** (0.0462)	0.1670* (0.0874)	-0.0964** (0.0457)	0.1837** (0.0758)	-0.0995** (0.0458)
Working capital investment	0.0666 (0.1125)	-0.0644 (0.0394)	0.1493* (0.0790)	-0.0646* (0.0389)	0.1392** (0.0677)	-0.0633 (0.0389)
Small loan	-0.1849 (0.1491)		-0.1123 (0.1118)		-0.0153 (0.1054)	
Medium loan	-0.1364 (0.1635)		-0.1739 (0.1271)		0.0393 (0.1161)	
Medium-large loan	-0.0731 (0.1721)		-0.3251** (0.1326)		-0.0863 (0.1201)	
Large loan	-0.4279* (0.2208)		-0.6115*** (0.1606)		-0.2937** (0.1380)	
Construction	0.2761* (0.1597)	-0.0326 (0.0688)	0.1897 (0.1268)	-0.0325 (0.0684)	0.1712 (0.1150)	-0.0336 (0.0688)
Trade	-0.0295 (0.1400)	-0.0386 (0.0531)	-0.0425 (0.1030)	-0.0383 (0.0527)	0.0407 (0.0889)	-0.0400 (0.0529)
Services	0.1699 (0.1341)	-0.1273*** (0.0489)	0.1114 (0.0929)	-0.1276*** (0.0482)	0.0935 (0.0816)	-0.1308*** (0.0484)
Branch density	-0.0255*** (0.0080)	0.0183*** (0.0027)	-0.0163*** (0.0058)	0.0182*** (0.0027)	-0.0138*** (0.0049)	0.0182*** (0.0027)
HHI	1.3569* (0.7899)	1.1020*** (0.4009)	0.6789* (0.4024)	1.0922*** (0.3982)	0.0481 (0.7376)	1.0790*** (0.3976)
Cooperative banks	-0.0014 (0.0051)	0.0058*** (0.0018)	-0.0055 (0.0036)	0.0057*** (0.0018)	-0.0104*** (0.0032)	0.0057*** (0.0018)
NPL ratio	-0.0019 (0.0108)	-0.0001 (0.0037)	0.0011 (0.0077)	-0.0002 (0.0037)	0.0024 (0.0066)	-0.0002 (0.0037)
Enforcing Contracts	-0.0021 (0.0087)	-0.0133*** (0.0032)	-0.0060 (0.0063)	-0.0132*** (0.0032)	-0.0107** (0.0053)	-0.0132*** (0.0031)
Increased production costs		0.0457 (0.0368)		0.0422 (0.0365)		0.0363 (0.0365)
Pessimistic expectations		0.5360*** (0.0459)		0.5424*** (0.0456)		0.5402*** (0.0459)
Intercept	-0.6603 (0.8469)	-1.0846*** (0.2769)	-0.0013 (0.6017)	-1.0867*** (0.2742)	-0.1972 (0.5159)	-1.0864*** (0.2743)
Time fixed-effects	Yes	Yes	Yes	Yes	Yes	Yes
Country group fixed-effects	Yes	Yes	Yes	Yes	Yes	Yes
Random effects						
σ_α	1.2689*** (0.0934)		1.0632*** (0.0589)		0.9674*** (0.0468)	
σ_η	0.8462*** (0.0246)		0.8413*** (0.0248)		0.8402*** (0.0252)	
$\rho_{\alpha\eta}$	-0.4182*** (0.0399)		-0.3662*** (0.0581)		-0.2081*** (0.0558)	
Idiosyncratic errors						
$\rho_{\varepsilon\mu}$	-0.8948*** (0.0424)		-0.8328*** (0.0327)		-0.8553*** (0.0267)	
Total correlation (ρ_{tot})	-0.6350*** (0.0412)		-0.6083*** (0.0314)		-0.6040*** (0.0246)	
Number of observations	18731		18731		18731	
Log-likelihood	-11972.16		-12806670		-13553.63	

Notes: the Table reports the coefficients of the extended static random-effects model *c)* estimated on the same sample used for the estimation of the dynamic model (i.e., considering only those firms observed for at least two consecutive periods) and using, alternatively, *Rationing*, *Rationing 2* and *Rationing 3* as indicators of financing constraints. Standard errors are reported in parentheses. *Large*, *≥10 years*, *Micro loan* and *Industry* are used as base levels for the categorical variables of firm size, firm age, loan size and sector, respectively.
***, **, * denote significance at 1, 5 and 10% levels, respectively.

Table S11 – Robustness analysis: estimates of the static model of firms' access to credit controlling for a firm's credit score

	Model c)				*Model c)*				*Model c)*			
	Rationing		Loan demand		Rationing 2		Loan demand		Rationing 3		Loan demand	
Credit score	-0.1490***	(0.0172)	0.0532***	(0.0056)	-0.1040***	(0.0111)	0.0533***	(0.0055)	-0.0867***	(0.0096)	0.0533***	(0.0054)
Autonomous firm	-0.1669	(0.1118)	0.2140***	(0.0375)	-0.2080***	(0.0750)	0.2145***	(0.0369)	-0.2324***	(0.0637)	0.2144***	(0.0366)
Individual/family-owned	0.1888**	(0.0931)	-0.0511	(0.0315)	0.1198*	(0.0617)	-0.0507	(0.0310)	0.1292**	(0.0532)	-0.0500	(0.0308)
Public support improved	-0.5494***	(0.1600)	0.0723*	(0.0419)	-0.3698***	(0.0909)	0.0738*	(0.0413)	-0.1536**	(0.0699)	0.0735*	(0.0409)
Credit history deteriorated	0.5963***	(0.0875)	0.2390***	(0.0396)	0.4435***	(0.0609)	0.2360***	(0.0388)	0.3665***	(0.0565)	0.2359***	(0.0388)
Fixed investments	-0.6655***	(0.0734)	0.6400***	(0.0235)	-0.5835***	(0.0462)	0.6405***	(0.0231)	-0.5161***	(0.0404)	0.6407***	(0.0230)
New products investment	0.3731***	(0.0786)	-0.1146***	(0.0296)	0.2165***	(0.0525)	-0.1161***	(0.0291)	0.2295***	(0.0466)	-0.1166***	(0.0290)
Working capital investment	0.0081	(0.0745)	0.0545**	(0.0244)	0.0838*	(0.0472)	0.0544**	(0.0241)	0.0772*	(0.0412)	0.0539**	(0.0240)
Small loan	-0.1393	(0.1018)			-0.0796	(0.0664)			0.0239	(0.0612)		
Medium loan	-0.0575	(0.1113)			-0.0619	(0.0732)			0.0790	(0.0665)		
Medium-large loan	-0.2244**	(0.1145)			-0.2282***	(0.0753)			-0.0442	(0.0677)		
Large loan	-0.4787***	(0.1352)			-0.4494***	(0.0863)			-0.1588**	(0.0749)		
Construction	0.2315**	(0.1156)	-0.1536***	(0.0424)	0.1207	(0.0795)	-0.1546***	(0.0417)	0.1892***	(0.0697)	-0.1545***	(0.0416)
Trade	0.0184	(0.0966)	-0.0906***	(0.0333)	-0.0240	(0.0621)	-0.0911***	(0.0327)	0.0051	(0.0540)	-0.0912***	(0.0326)
Services	0.1979**	(0.0875)	-0.1468***	(0.0293)	0.1364**	(0.0550)	-0.1478***	(0.0288)	0.1231**	(0.0479)	-0.1485***	(0.0287)
Branch density	-0.0163***	(0.0049)	0.0113***	(0.0017)	-0.0136***	(0.0032)	0.0113***	(0.0016)	-0.0134***	(0.0028)	0.0112***	(0.0016)
HHI	1.2354*	(0.6752)	0.6611***	(0.2389)	0.4077*	(0.2457)	0.6584***	(0.2340)	0.0411	(0.3945)	0.6497***	(0.2340)
Cooperative banks	-0.0001	(0.0032)	0.0033***	(0.0011)	-0.0065***	(0.0021)	0.0033***	(0.0011)	-0.0101***	(0.0019)	0.0034***	(0.0011)
NPL ratio	0.0108	(0.0067)	-0.0001	(0.0023)	0.0065	(0.0045)	-0.0001	(0.0023)	0.0029	(0.0039)	-0.0001	(0.0023)
Enforcing Contracts	-0.0098*	(0.0059)	-0.0044**	(0.0020)	-0.0086**	(0.0038)	-0.0044**	(0.0019)	-0.0083**	(0.0032)	-0.0045**	(0.0019)
Increased production costs			0.0026	(0.0227)			0.0022	(0.0223)			0.0057	(0.0222)
Pessimistic expectations			0.5639***	(0.0295)			0.5650***	(0.0289)			0.5650***	(0.0288)
Intercept	-0.1478	(0.5726)	-2.2778***	(0.1908)	1.3425***	(0.3802)	-2.2794***	(0.1884)	1.5166***	(0.3284)	-2.2743***	(0.1875)
Time fixed-effects	Yes		Yes		Yes		Yes		Yes		Yes	
Country group fixed-effects	Yes		Yes		Yes		Yes		Yes		Yes	
Random effects												
σ_α	1.2439***	(0.0501)			0.9347***	(0.0300)			0.8732***	(0.0250)		
σ_η	0.7617***	(0.0115)			0.7615***	(0.0119)			0.7607***	(0.0120)		
$\rho_{\alpha\eta}$	-0.2678***	(0.0506)			-0.3282***	(0.0375)			-0.3165***	(0.0338)		
Idiosyncratic errors												
$\rho_{\varepsilon\mu}$	-0.5692***	(0.0370)			-0.7523***	(0.0194)			-0.7738***	(0.0158)		
Total correlation (ρ_{tot})	-0.4102***	(0.0289)			-0.5730***	(0.0173)			-0.5899***	(0.0137)		
Number of observations	58367				58367				58367			
Log-likelihood	-35177.35				-37331.14				-39193,24			

Notes: the Table reports the coefficients of the extended static random-effects model *c)* in which a raw credit score (based on the age and size of the firm and on changes in turnover and profit) is included as a control for firm creditworthiness (simultaneously excluding its components from the set of regressors) and using, alternatively, *Rationing*, *Rationing 2* and *Rationing 3* as indicators of financing constraints. Standard errors are reported in parentheses. *Micro loan* and *Industry* are used as base levels for the categorical variables of loan size and sector, respectively.

***, **, * denote significance at 1, 5 and 10% levels, respectively.

Table S12 – Robustness analysis: estimates of the dynamic model of firms' access to credit controlling for a firm's credit score

	Model dyn c)		*Model dyn c)*		*Model dyn c)*	
	Rationing	Loan demand	Rationing 2	Loan demand	Rationing 3	Loan demand
R^*_{t-1}	1.4415*** (0.1734)	-0.1014 (0.0910)	0.9125*** (0.1353)	-0.0162 (0.0663)	0.6913*** (0.1089)	0.0220 (0.0557)
D_{t-1}	-0.3678*** (0.0790)	0.5379*** (0.0337)	-0.2398*** (0.0702)	0.5332*** (0.0341)	-0.1794*** (0.0649)	0.5266*** (0.0347)
Credit score	-0.0606*** (0.0216)	0.0457*** (0.0085)	-0.0604*** (0.0184)	0.0462*** (0.0085)	-0.0565*** (0.0164)	0.0465*** (0.0085)
Autonomous firm	-0.4057* (0.2284)	0.1262 (0.1866)	-0.2449 (0.3874)	0.1290 (0.1869)	-0.3110 (0.3335)	0.1310 (0.1861)
Individual/family-owned	0.4485* (0.2611)	0.1701 (0.1210)	0.4803* (0.2793)	0.1696 (0.1212)	0.3892 (0.2619)	0.1661 (0.1216)
Public support improved	-0.4589** (0.2275)	0.0632 (0.0668)	-0.1696 (0.1752)	0.0624 (0.0668)	-0.2106 (0.1434)	0.0639 (0.0668)
Credit history deteriorated	0.1532 (0.1439)	0.1726** (0.0710)	0.3210** (0.1361)	0.1726** (0.0710)	0.3052** (0.1285)	0.1756** (0.0711)
Fixed investments	-0.3940*** (0.1185)	0.4060*** (0.0427)	-0.2129** (0.1039)	0.4058*** (0.0427)	-0.2083** (0.0941)	0.4068*** (0.0427)
New products investment	0.0783 (0.1383)	-0.1387*** (0.0526)	-0.0987 (0.1222)	-0.1382*** (0.0525)	0.0012 (0.1102)	-0.1397*** (0.0525)
Working capital investment	0.1146 (0.1230)	-0.2287*** (0.0448)	0.1007 (0.1056)	-0.2291*** (0.0448)	0.0404 (0.0959)	-0.2292*** (0.0448)
Small loan	-0.1592 (0.2620)		-0.2451 (0.2347)		-0.0018 (0.2360)	
Medium loan	-0.1293 (0.2995)		-0.2061 (0.2800)		0.1052 (0.2713)	
Medium-large loan	0.0545 (0.3236)		-0.2480 (0.3025)		0.0672 (0.2903)	
Large loan	0.0974 (0.3814)		-0.2710 (0.3664)		0.0572 (0.3392)	
Construction	0.2252* (0.1278)	-0.0417 (0.0523)	0.1727 (0.1167)	-0.0429 (0.0523)	0.1838* (0.1101)	-0.0430 (0.0523)
Trade	0.0750 (0.1043)	-0.0668* (0.0393)	-0.0298 (0.0927)	-0.0667* (0.0393)	0.0861 (0.0833)	-0.0670* (0.0393)
Services	0.0801 (0.0971)	-0.0761** (0.0342)	0.0541 (0.0821)	-0.0773** (0.0341)	0.0874 (0.0735)	-0.0777** (0.0341)
Branch density	-0.0126* (0.0065)	0.0122*** (0.0021)	-0.0088* (0.0049)	0.0122*** (0.0021)	-0.0099** (0.0048)	0.0123*** (0.0021)
HHI	1.9995** (0.9215)	0.5167* (0.3064)	0.7663* (0.4125)	0.5166* (0.3062)	0.1457 (0.7339)	0.5155* (0.3059)
Cooperative banks	0.0013 (0.0041)	0.0039*** (0.0014)	-0.0021 (0.0034)	0.0039*** (0.0014)	-0.0077** (0.0031)	0.0039*** (0.0014)
NPL ratio	-0.0065 (0.0086)	0.0013 (0.0029)	0.0010 (0.0071)	0.0012 (0.0029)	0.0013 (0.0063)	0.0012 (0.0029)
Enforcing Contracts	-0.0091 (0.0072)	-0.0102*** (0.0024)	-0.0012 (0.0061)	-0.0102*** (0.0024)	-0.0051 (0.0053)	-0.0102*** (0.0024)
Increased production costs		0.0271 (0.0279)		0.0264 (0.0278)		0.0257 (0.0278)
Pessimistic expectations		0.3774*** (0.0305)		0.3778*** (0.0305)		0.3801*** (0.0306)
Intercept	-0.0650 (0.6876)	-2.0218*** (0.2294)	-0.4114 (0.5894)	-2.0242*** (0.2294)	-0.5345 (0.5128)	-2.0314*** (0.2294)
Time fixed-effects	Yes	Yes	Yes	Yes	Yes	Yes
Country group fixed-effects	Yes	Yes	Yes	Yes	Yes	Yes
Initial conditions						
$R^*_{0_i}$	0.6797*** (0.1882)		0.6681*** (0.1295)		0.7498*** (0.1024)	
D_{0_i}		0.3992*** (0.0334)		0.3976*** (0.0334)		0.3978*** (0.0335)
Random effects						
σ_{a_2}	0.5786*** (0.0673)		0.7341*** (0.0539)		0.7509*** (0.0480)	
σ_{a_1}	0.4765*** (0.0205)		0.4764*** (0.205)		0.4775*** (0.0205)	
$\rho_{a_1 a_2}$	-0.0360 (0.1857)		-0.0733 (0.1210)		-0.0543 (0.1056)	
Idiosyncratic errors						
$\rho_{\varepsilon\mu}$	-0.2980*** (0.0539)		-0.2280*** (0.0462)		-0.2970*** (0.0405)	
Total correlation (ρ_{tot})	-0.2406*** (0.0492)		-0.1846*** (0.0411)		-0.2284*** (0.0385)	
Number of observations	18731		18731		18731	
Log-likelihood	-11076.27		-11893.92		-12620.18	

Notes: the Table reports the coefficients of the extended dynamic Model *dyn_*c) in which a raw credit score (based on the age and size of the firm and on changes in turnover and profit) is included as a control for firm creditworthiness (simultaneously excluding its components from the set of regressors) and using, alternatively, *Rationing*, *Rationing 2* and *Rationing 3* as indicators of financing constraints. Standard errors are reported in parentheses. *Micro loan* and *Industry* are used as base levels for the categorical variables of loan size and sector, respectively.
***, **, * denote significance at 1, 5 and 10% levels, respectively.

Table S13 – Robustness analysis: estimates of the dynamic model of firms' access to credit focusing on firms needing external financing

	Model dyn c)		*Model dyn c)*		*Model dyn c)*	
	Rationing	Loan demand	Rationing 2	Loan demand	Rationing 3	Loan demand
R^*_{t-1}	1.5075*** (0.1750)	-0.7471*** (0.1757)	0.9827*** (0.1311)	-0.7481*** (0.1450)	0.7545*** (0.1071)	-0.5637*** (0.1343)
D_{t-1}	-0.2807*** (0.0817)	0.5317*** (0.0944)	-0.1810*** (0.0693)	0.6238*** (0.1009)	-0.0931 (0.0644)	0.6127*** (0.1036)
Micro	0.6643*** (0.2093)	-0.7262*** (0.1701)	0.1305 (0.1688)	-0.7341*** (0.1712)	0.1430 (0.1442)	-0.7525*** (0.1734)
Small	0.3362* (0.1956)	-0.3675** (0.1664)	0.0297 (0.1516)	-0.3611** (0.1682)	0.0598 (0.1306)	-0.3758** (0.1704)
Medium	-0.0073 (0.1905)	-0.0317 (0.1698)	-0.1064 (0.1394)	-0.0126 (0.1719)	-0.0361 (0.1194)	-0.0209 (0.1740)
Autonomous firm	-0.4614* (0.2357)	0.4439 (0.5600)	-0.2568 (0.3835)	0.4175 (0.5713)	-0.3213 (0.3299)	0.4519 (0.5641)
Individual/family-owned	0.5184* (0.2690)	-0.0802 (0.3248)	0.5029* (0.2793)	-0.0569 (0.3352)	0.4455* (0.2574)	-0.1147 (0.3349)
<5 years	-0.4702 (0.5229)	0.6817 (0.4564)	-0.1775 (0.4259)	0.7227 (0.4808)	0.1484 (0.3817)	0.7041 (0.4710)
≥5 and <10 years	-0.3854 (0.3303)	-0.1513 (0.2848)	-0.0444 (0.2833)	-0.1501 (0.2892)	0.0148 (0.2424)	-0.1552 (0.2933)
Turnover decreased	0.0178 (0.1418)	-0.1234 (0.1353)	0.0659 (0.1220)	-0.1443 (0.1383)	0.0681 (0.1116)	-0.1341 (0.1400)
Profit decreased	0.1969 (0.1290)	0.0588 (0.1240)	0.0290 (0.1062)	0.0613 (0.1270)	0.0412 (0.0974)	0.0626 (0.1279)
Own capital deteriorated	-0.0127 (0.1666)	-0.3397** (0.1699)	0.0996 (0.1469)	-0.3462** (0.1728)	0.3451** (0.1415)	-0.3514** (0.1737)
Public support improved	-0.5102** (0.2370)	0.3296 (0.2182)	-0.1975 (0.1746)	0.2769 (0.2237)	-0.2382* (0.1442)	0.2955 (0.2228)
Credit history deteriorated	0.2934* (0.1536)	0.1041 (0.1657)	0.3944*** (0.1364)	0.0866 (0.1683)	0.3339*** (0.1294)	0.0883 (0.1697)
Fixed investments	-0.4093*** (0.1211)	0.3186*** (0.1175)	-0.1941* (0.1023)	0.3066** (0.1195)	-0.1659* (0.0930)	0.3058** (0.1199)
New products investment	0.0694 (0.1404)	-0.0903 (0.1386)	-0.1051 (0.1202)	-0.0733 (0.1423)	-0.0026 (0.1091)	-0.0960 (0.1431)
Working capital investment	0.1007 (0.1280)	-0.1589 (0.1205)	0.0892 (0.1036)	-0.1678 (0.1221)	0.0258 (0.0949)	-0.1653 (0.1230)
Small loan	-0.1652 (0.2759)		-0.2544 (0.2337)		-0.0221 (0.2373)	
Medium loan	-0.0744 (0.3188)		-0.1830 (0.2793)		0.1122 (0.2717)	
Medium-large loan	0.1153 (0.3432)		-0.2451 (0.3017)		0.0556 (0.2896)	
Large loan	0.1661 (0.4136)		-0.2613 (0.3679)		0.0388 (0.3415)	
Construction	0.1696 (0.1339)	-0.0339 (0.1385)	0.1581 (0.1164)	-0.0339 (0.1412)	0.1700 (0.1105)	-0.0377 (0.1399)
Trade	-0.0473 (0.1112)	0.0878 (0.1062)	-0.0806 (0.0936)	0.1025 (0.1084)	0.0419 (0.0843)	0.0891 (0.1092)
Services	0.0282 (0.1051)	-0.0378 (0.0991)	0.0236 (0.0848)	-0.0356 (0.1016)	0.0652 (0.0775)	-0.0444 (0.1022)
Branch density	-0.0143** (0.0066)	0.0202*** (0.0056)	-0.0093* (0.0046)	0.0198*** (0.0057)	-0.0089* (0.0047)	0.0202*** (0.0057)
HHI	2.1382** (0.9266)	-0.1284 (0.8193)	0.8901* (04898)	-0.1214 (0.8318)	0.0492 (0.7278)	-0.0570 (0.8331)
Cooperative banks	-0.0011 (0.0041)	0.0086** (0.0036)	-0.0036 (0.0033)	0.0082** (0.0037)	-0.0083*** (0.0030)	0.0084** (0.0037)
NPL ratio	-0.0030 (0.0088)	-0.0185** (0.0073)	0.0036 (0.0069)	-0.0200*** (0.0074)	0.0042 (0.0062)	-0.0204*** (0.0075)
Enforcing Contracts	-0.0063 (0.0074)	-0.0123** (0.0062)	-0.0022 (0.0060)	-0.0114* (0.0062)	-0.0056 (0.0053)	-0.0119* (0.0063)
Increased production costs		0.0221 (0.0741)		0.0103 (0.0758)		0.0023 (0.0762)
Pessimistic expectations		0.0423 (0.0794)		0.0570 (0.0809)		0.0650 (0.0815)
Intercept	-1.5245** (0.7329)	1.2541** (0.6274)	-1.1765** (0.5964)	1.1936* (0.6373)	-1.4599*** (0.5321)	1.2866** (0.6410)
Time fixed-effects	Yes	Yes	Yes	Yes	Yes	Yes
Country group fixed-effects	Yes	Yes	Yes	Yes	Yes	Yes
Initial conditions						
$R^*_{0_i}$	0.6413*** (0.1888)		0.6001*** (0.1260)		0.7156*** (0.1010)	
D_{0_i}		0.2449*** (0.0904)		0.2480*** (0.0926)		0.2518*** (0.0928)
Random effects						
σ_{a_2}	0.6902*** (0.0538)		0.7647*** (0.0452)		0.7802*** (0.0432)	
σ_{a_1}	1.0484*** (0.0404)		1.0720*** (0.0410)		1.0807*** (0.0410)	
$\rho_{a_1 a_2}$	-0.7384*** (0.11799)		-0.6121*** (0.1026)		-0.5816*** (0.1013)	
Idiosyncratic errors						
$\rho_{\varepsilon\mu}$	-0.1983** (0.0889)		-0.1733** (0.0958)		-0.1801** (0.0896)	
Total correlation (ρ_{tot})	-0.4162*** (0.0836)		-0.3658*** (0.0872)		-0.5170*** (0.0217)	
Number of observations	8274		8274		8274	
Log-likelihood	-3228.60		-4044.57		-4780.34	

Notes: the Table reports the coefficients of the extended dynamic model *dyn_c)* estimated on the subsample of firms needing external financing (i.e., excluding those firm that do not apply because of sufficient internal funds) and using, alternatively, *Rationing*, *Rationing 2* and *Rationing 3* as indicators of financing constraints. Standard errors are reported in parentheses. *Large*, *≥10 years*, *Micro loan* and *Industry* are used as base levels for the categorical variables of firm size, firm age, loan size and sector, respectively.
***, **, * denote significance at 1, 5 and 10% levels, respectively.

Table S14 – Robustness analysis: estimates of the dynamic model of firms' access to credit focusing on firms with at least four not necessarily consecutive observations

	Model dyn c)		*Model dyn c)*		*Model dyn c)*	
	Rationing	Loan demand	Rationing 2	Loan demand	Rationing 3	Loan demand
R^*_{t-1}	1.3717*** (0.1927)	-0.0021 (0.1148)	0.9008*** (0.1542)	0.0460 (0.0829)	0.6953*** (0.1246)	0.0570 (0.0687)
D_{t-1}	-0.3940*** (0.0929)	0.5094*** (0.0379)	-0.1805** (0.0826)	0.5041*** (0.0384)	-0.1685** (0.0772)	0.4987*** (0.0392)
Micro	0.8948*** (0.2497)	-0.4164*** (0.0707)	0.2222 (0.2061)	-0.4172*** (0.0705)	0.1449 (0.1785)	-0.4181*** (0.0706)
Small	0.4988** (0.2300)	-0.2410*** (0.0657)	0.0414 (0.1827)	-0.2415*** (0.0656)	0.0290 (0.1592)	-0.2425*** (0.0657)
Medium	0.2040 (0.2168)	-0.1162* (0.0645)	-0.0177 (0.1660)	-0.1161* (0.0643)	-0.0606 (0.1434)	-0.1172* (0.0645)
Autonomous firm	-0.5003* (0.2595)	0.1003 (0.2018)	-0.2551 (0.4265)	0.1013 (0.2027)	-0.3329 (0.3660)	0.1041 (0.2021)
Individual/family-owned	0.5027* (0.2941)	0.1681 (0.1315)	0.4780 (0.3205)	0.1685 (0.1318)	0.4587 (0.2897)	0.1658 (0.1321)
<5 years	-0.6121 (0.5621)	0.1752 (0.1994)	-0.2828 (0.4845)	0.1751 (0.1991)	0.1320 (0.4439)	0.1729 (0.1999)
≥5 and <10 years	-0.7232* (0.3739)	-0.0571 (0.1370)	-0.2656 (0.3476)	-0.0568 (0.1373)	-0.0708 (0.2853)	-0.0583 (0.1373)
Turnover decreased	0.0436 (0.1550)	-0.0613 (0.0586)	0.0681 (0.1383)	-0.0627 (0.0586)	0.0236 (0.1247)	-0.0623 (0.0587)
Profit decreased	0.1878 (0.1385)	0.0954* (0.0516)	0.0235 (0.1188)	0.0956* (0.0516)	0.0651 (0.1085)	0.0954* (0.0516)
Own capital deteriorated	-0.1814 (0.1763)	0.1348* (0.0809)	0.0106 (0.1613)	0.1352* (0.0810)	0.2852* (0.1547)	0.1360* (0.0810)
Public support improved	-0.3869 (0.2426)	0.1045 (0.0720)	-0.0795 (0.1781)	0.1043 (0.0720)	-0.2394 (0.1558)	0.1053 (0.0721)
Credit history deteriorated	0.3146* (0.1646)	0.0900 (0.0789)	0.4427*** (0.1517)	0.0890 (0.0789)	0.3551** (0.1424)	0.0912 (0.0790)
Fixed investments	-0.4908*** (0.1302)	0.4160*** (0.0460)	-0.1964* (0.1148)	0.4158*** (0.0460)	-0.2308** (0.1041)	0.4165*** (0.0460)
New products investment	0.0523 (0.1507)	-0.1420** (0.0572)	-0.1566 (0.1364)	-0.1412** (0.0571)	0.0047 (0.1217)	-0.1420** (0.0572)
Working capital investment	0.1189 (0.1345)	-0.2380*** (0.0485)	0.1110 (0.1151)	-0.2381*** (0.0485)	0.0133 (0.1055)	-0.2381*** (0.0486)
Small loan	-0.1695 (0.2898)		-0.2734 (0.2561)		0.0175 (0.2613)	
Medium loan	-0.0748 (0.3390)		-0.2489 (0.3051)		0.0907 (0.3004)	
Medium-large loan	-0.0565 (0.3636)		-0.3951 (0.3250)		0.0202 (0.3182)	
Large loan	0.0234 (0.4216)		-0.4565 (0.3870)		0.0546 (0.3689)	
Construction	0.1507 (0.1494)	-0.0163 (0.0632)	0.1565 (0.1375)	-0.0174 (0.0632)	0.1550 (0.1314)	-0.0187 (0.0633)
Trade	0.0252 (0.1246)	-0.0042 (0.0473)	0.0133 (0.1104)	-0.0038 (0.0474)	0.0995 (0.0985)	-0.0043 (0.0474)
Services	0.0244 (0.1186)	-0.0775* (0.0440)	0.0365 (0.1013)	-0.0791* (0.0440)	0.0946 (0.0927)	-0.0797* (0.0440)
Branch density	-0.0063 (0.0075)	0.0137*** (0.0026)	-0.0034 (0.0067)	0.0137*** (0.0026)	-0.0053 (0.0058)	0.0137*** (0.0026)
HHI	1.9802* (1.0827)	0.9534** (0.3810)	1.0387* (0.5801)	0.9515** (0.3809)	0.0448 (0.9088)	0.9455** (0.3809)
Cooperative banks	0.0029 (0.0047)	0.0048*** (0.0017)	-0.0008 (0.0041)	0.0048*** (0.0017)	-0.0052 (0.0037)	0.0048*** (0.0017)
NPL ratio	0.0051 (0.0098)	0.0016 (0.0035)	0.0013 (0.0087)	0.0015 (0.0035)	0.0052 (0.0076)	0.0015 (0.0035)
Enforcing Contracts	-0.0023 (0.0086)	-0.0127*** (0.0030)	-0.0031 (0.0073)	-0.0126*** (0.0030)	-0.0082 (0.0065)	-0.0126*** (0.0030)
Increased production costs		0.0597* (0.0337)		0.0591* (0.0337)		0.0586* (0.0338)
Pessimistic expectations		0.3816*** (0.0371)		0.3785*** (0.0371)		0.3789*** (0.0372)
Intercept	-2.0870** (0.8237)	-1.4778*** (0.2724)	-1.4842** (0.7296)	-1.4797*** (0.2724)	-1.7070*** (0.6552)	-1.4846*** (0.2728)
Time fixed-effects	Yes	Yes	Yes	Yes	Yes	Yes
Country group fixed-effects	Yes	Yes	Yes	Yes	Yes	Yes
Initial conditions						
$R^*_{0_i}$	0.7037*** (0.2091)		0.6654*** (0.1507)		0.7913*** (0.1161)	
D_{0_i}		0.4377*** (0.0383)		0.4375*** (0.0383)		0.4384*** (0.0384)
Random effects						
σ_{a_2}	0.6015*** (0.0691)		0.7526*** (0.0580)		0.7630*** (0.0506)	
σ_{a_1}	0.4797*** (0.0215)		0.4792*** (0.0215)		0.4795*** (0.0215)	
$\rho_{a_1 a_2}$	-0.1303 (0.1828)		-0.1213 (0.1237)		-0.0314 (0.0368)	
Idiosyncratic errors						
$\rho_{\varepsilon\mu}$	-0.3163*** (0.0593)		-0.1877*** (0.0530)		-0.2554*** (0.0450)	
Total correlation (ρ_{tot})	-0.2734*** (0.0551)		-0.1668*** (0.0487)		-0.1913*** (0.0425)	
Number of observations	14357		14357		14357	
Log-likelihood	-8455.14		-9111.86		-9700.89	

Notes: the Table reports the coefficients of the extended dynamic model *dyn_c)* estimated on the subsample of firms with at least four observations and using, alternatively, *Rationing*, *Rationing 2* and *Rationing 3* as indicators of financing constraints. Standard errors are reported in parentheses. *Large*, *≥10 years*, *Micro loan* and *Industry* are used as base levels for the categorical variables of firm size, firm age, loan size and sector, respectively. ***, **, * denote significance at 1, 5 and 10% levels, respectively.

Table S15 – Robustness analysis: estimates of the dynamic model of firms' access to credit focusing on firms with at least three consecutive observations

	Model dyn c)		*Model dyn c)*		*Model dyn c)*	
	Rationing	Loan demand	Rationing 2	Loan demand	Rationing 3	Loan demand
R^*_{t-1}	1.2387*** (0.2356)	-0.0781 (0.1301)	0.6788*** (0.1873)	-0.0194 (0.0899)	0.4615*** (0.1529)	-0.0078 (0.0746)
D_{t-1}	-0.3554*** (0.1024)	0.4858*** (0.0409)	-0.1620* (0.0939)	0.4828*** (0.0416)	-0.0976 (0.0896)	0.4807*** (0.0424)
Micro	0.8815*** (0.2661)	-0.3836*** (0.0747)	0.2622 (0.2336)	-0.3837*** (0.0747)	0.2315 (0.2037)	-0.3841*** (0.0749)
Small	0.3836 (0.2431)	-0.2095*** (0.0702)	0.0619 (0.2067)	-0.2086*** (0.0703)	0.1168 (0.1815)	-0.2092*** (0.0704)
Medium	0.0423 (0.2358)	-0.1183* (0.0691)	-0.0199 (0.1896)	-0.1173* (0.0691)	0.0106 (0.1664)	-0.1182* (0.0693)
Autonomous firm	-1.0637*** (0.3183)	0.2791 (0.2362)	-0.2723 (0.5230)	0.2837 (0.2375)	-0.2113 (0.4611)	0.2862 (0.2364)
Individual/family-owned	0.5424 (0.3311)	0.1788 (0.1460)	0.4244 (0.3635)	0.1803 (0.1462)	0.3859 (0.3361)	0.1771 (0.1466)
<5 years	-0.8540 (0.6567)	0.0100 (0.2284)	-0.2065 (0.5741)	0.0100 (0.2288)	-0.0570 (0.5091)	0.0061 (0.2283)
≥5 and <10 years	-0.3984 (0.4324)	-0.0602 (0.1542)	-0.0101 (0.3837)	-0.0604 (0.1542)	-0.0226 (0.3292)	-0.0617 (0.1542)
Turnover decreased	0.0724 (0.1772)	-0.0594 (0.0642)	0.0018 (0.1627)	-0.0614 (0.0643)	-0.0511 (0.1495)	-0.0612 (0.0644)
Profit decreased	0.3584* (0.1925)	0.1193** (0.0566)	0.0984 (0.1406)	0.1197** (0.0567)	0.0995 (0.1289)	0.1198** (0.0567)
Own capital deteriorated	-0.0483 (0.1964)	0.0858 (0.0875)	0.0935 (0.1855)	0.0853 (0.0878)	0.3078* (0.1814)	0.0845 (0.0878)
Public support improved	-0.4378 (0.3014)	0.0834 (0.0783)	-0.1092 (0.2163)	0.0819 (0.0784)	-0.2344 (0.1910)	0.0838 (0.0786)
Credit history deteriorated	0.3312* (0.1905)	0.1366 (0.0857)	0.4452** (0.1756)	0.1368 (0.0859)	0.4029** (0.1677)	0.1389 (0.0860)
Fixed investments	-0.4867*** (0.1445)	0.4122*** (0.0505)	-0.1194 (0.1333)	0.4122*** (0.0506)	-0.1888 (0.1225)	0.4132*** (0.0507)
New products investment	0.0831 (0.1639)	-0.1130* (0.0628)	-0.1429 (0.1544)	-0.1125* (0.0628)	-0.0051 (0.1421)	-0.1134* (0.0629)
Working capital investment	0.1092 (0.1521)	-0.2720*** (0.0529)	0.1489 (0.1325)	-0.2720*** (0.0529)	0.0772 (0.1239)	-0.2715*** (0.0530)
Small loan	-0.2761 (0.3385)		-0.2776 (0.3074)		0.0640 (0.3092)	
Medium loan	-0.0803 (0.3908)		-0.1890 (0.3654)		0.1736 (0.3566)	
Medium-large loan	0.0927 (0.4190)		-0.2730 (0.3930)		0.1095 (0.3802)	
Large loan	0.1061 (0.4798)		-0.2811 (0.4686)		0.1066 (0.4405)	
Construction	0.2006 (0.1718)	-0.0680 (0.0695)	0.1833 (0.1611)	-0.0694 (0.0696)	0.2223 (0.1587)	-0.0697 (0.0696)
Trade	0.0628 (0.1355)	-0.0211 (0.0511)	0.0210 (0.1251)	-0.0217 (0.0512)	0.1158 (0.1152)	-0.0227 (0.0512)
Services	-0.0387 (0.1349)	-0.0980** (0.0473)	0.0496 (0.1159)	-0.0990** (0.0473)	0.1628 (0.1077)	-0.0994** (0.0474)
Branch density	-0.0146* (0.0084)	0.0126*** (0.0029)	-0.0039 (0.0076)	0.0126*** (0.0029)	-0.0111 (0.0068)	0.0126*** (0.0029)
HHI	0.9308* (0.5462)	0.7082* (0.4112)	0.7703* (0.4571)	0.7096* (0.4114)	0.6156 (1.0386)	0.7071* (0.4112)
Cooperative banks	0.0011 (0.0054)	0.0040** (0.0018)	0.0001 (0.0047)	0.0040** (0.0018)	-0.0064 (0.0043)	0.0040** (0.0018)
NPL ratio	0.0056 (0.0114)	0.0023 (0.0038)	0.0051 (0.0101)	0.0023 (0.0038)	0.0060 (0.0091)	0.0023 (0.0038)
Enforcing Contracts	-0.0010 (0.0098)	-0.0118*** (0.0033)	-0.0043 (0.0085)	-0.0118*** (0.0033)	-0.0120 (0.0076)	-0.0117*** (0.0033)
Increased production costs		0.0651* (0.0363)		0.0636* (0.0363)		0.0641* (0.0363)
Pessimistic expectations		0.3658*** (0.0398)		0.3628*** (0.0399)		0.3651*** (0.0400)
Intercept	-2.8660*** (0.9589)	-1.5499*** (0.2932)	-1.8300** (0.8392)	-1.5515*** (0.2936)	-1.9423** (0.7655)	-1.5547*** (0.2940)
Time fixed-effects	Yes	Yes	Yes	Yes	Yes	Yes
Country group fixed-effects	Yes	Yes	Yes	Yes	Yes	Yes
Initial conditions						
$R^*_{0_i}$	0.9701*** (0.2432)		0.8165*** (0.1736)		0.8804*** (0.1394)	
D_{0_i}		0.4622*** (0.0406)		0.4630*** (0.0406)		0.4644*** (0.0407)
Random effects						
σ_{a_2}	0.6925*** (0.0708)		0.8626*** (0.0617)		0.8857*** (0.0546)	
σ_{a_1}	0.4977*** (0.0216)		0.4981*** (0.0216)		0.4988*** (0.0216)	
$\rho_{a_1 a_2}$	-0.0729 (0.1706)		-0.0373 (0.1175)		-0.0062 (0.1023)	
Idiosyncratic errors						
$\rho_{\varepsilon\mu}$	-0.3208*** (0.0634)		-0.1768*** (0.0581)		-0.2830*** (0.0493)	
Total correlation (ρ_{tot})	-0.2546*** (0.0596)		-0.1307*** (0.0471)		-0.1914*** (0.0443)	
Number of observations	13466		13466		13466	
Log-likelihood	-7897.27		-8531.07		-9078.82	

Notes: the Table reports the coefficients of the extended dynamic model *dyn_c)* estimated on the subsample of firms with at least three consecutive observations and using, alternatively, *Rationing*, *Rationing 2* and *Rationing 3* as indicators of financing constraints. Standard errors are reported in parentheses. *Large*, *≥10 years*, *Micro loan* and *Industry* are used as base levels for the categorical variables of firm size, firm age, loan size and sector, respectively.

***, **, * denote significance at 1, 5 and 10% levels, respectively.

Table S16 – Robustness analysis: estimates of the dynamic model of firms' access to credit using a one-year time frame

	Model dyn c)		*Model dyn c)*		*Model dyn c)*	
	Rationing	Loan demand	Rationing 2	Loan demand	Rationing 3	Loan demand
R^*_{t-1}	0.9790*** (0.3034)	0.0174 (0.1202)	0.6022*** (0.1625)	0.0405 (0.0985)	0.5811*** (0.1154)	0.0597 (0.0719)
D_{t-1}	-0.3046** (0.1518)	0.2864*** (0.0456)	-0.1773** (0.0808)	0.2803*** (0.0462)	-0.1688** (0.0676)	0.2778*** (0.0469)
Micro	0.9254*** (0.3024)	-0.5473*** (0.0736)	0.3499* (0.1978)	-0.5460*** (0.0735)	0.2492 (0.1567)	-0.5450*** (0.0738)
Small	0.4667* (0.2748)	-0.3099*** (0.0701)	0.2305 (0.1760)	-0.3080*** (0.0699)	0.2443* (0.1386)	-0.3072*** (0.0702)
Medium	0.0559 (0.2657)	-0.1858*** (0.0686)	-0.0314 (0.1610)	-0.1848*** (0.0684)	0.0553 (0.1272)	-0.1840*** (0.0687)
Autonomous firm	-0.7893* (0.4308)	0.0358 (0.2466)	-0.4607 (0.3765)	0.0311 (0.2448)	-0.0200 (0.4046)	0.0329 (0.2461)
Individual/family-owned	-0.3950 (0.5785)	-0.0162 (0.1578)	0.1441 (0.3439)	-0.0103 (0.1577)	-0.0115 (0.2763)	-0.0093 (0.1579)
<5 years	-0.7065 (0.7635)	-0.1028 (0.2524)	0.0151 (0.4639)	-0.0979 (0.2518)	0.2594 (0.4150)	-0.1051 (0.2522)
≥5 and <10 years	-0.2905 (0.5061)	-0.1737 (0.1509)	0.0368 (0.3183)	-0.1691 (0.1506)	-0.1350 (0.2600)	-0.1732 (0.1503)
Turnover decreased	0.2326 (0.2090)	0.0255 (0.0684)	0.1211 (0.1374)	0.0238 (0.0682)	0.1756 (0.1139)	0.0253 (0.0684)
Profit decreased	0.1421 (0.1836)	0.0688 (0.0595)	0.0155 (0.1185)	0.0669 (0.0593)	0.0477 (0.0997)	0.0654 (0.0594)
Own capital deteriorated	0.0133 (0.2395)	0.1327 (0.0904)	-0.0047 (0.1664)	0.1283 (0.0903)	0.0707 (0.1432)	0.1297 (0.0904)
Public support improved	-0.6156 (0.3828)	0.0237 (0.0840)	-0.2405 (0.1895)	0.0220 (0.0837)	-0.0542 (0.1413)	0.0225 (0.0838)
Credit history deteriorated	0.2057 (0.2228)	0.0955 (0.0893)	0.2289 (0.1569)	0.0956 (0.0892)	0.2797** (0.1335)	0.0974 (0.0893)
Fixed investments	-0.5952*** (0.1749)	0.4897*** (0.0530)	-0.3135*** (0.1148)	0.4887*** (0.0529)	-0.2524*** (0.0950)	0.4907*** (0.0530)
New products investment	0.0728 (0.1869)	-0.1298* (0.0668)	-0.1085 (0.1283)	-0.1289* (0.0665)	0.0070 (0.1055)	-0.1286* (0.0666)
Working capital investment	0.1867 (0.1827)	-0.2717*** (0.0558)	0.1364 (0.1179)	-0.2708*** (0.0557)	0.1800* (0.0979)	-0.2701*** (0.0559)
Small loan	-0.6457 (0.4048)		-0.2759 (0.2582)		-0.0645 (0.2252)	
Medium loan	-0.4918 (0.4775)		-0.2497 (0.2982)		0.0077 (0.2591)	
Medium-large loan	-0.2967 (0.5051)		-0.3242 (0.3222)		-0.0299 (0.2798)	
Large loan	-0.1576 (0.5824)		-0.2599 (0.3920)		0.1185 (0.3261)	
Construction	0.4638** (0.1968)	-0.1111* (0.0661)	0.2126 (0.1348)	-0.1103* (0.0659)	0.2251** (0.1133)	-0.1107* (0.0661)
Trade	0.0994 (0.1573)	-0.0581 (0.0505)	-0.0110 (0.1055)	-0.0581 (0.0503)	0.0361 (0.0864)	-0.0590 (0.0504)
Services	0.1048 (0.1574)	-0.1601*** (0.0468)	-0.0417 (0.0986)	-0.1594*** (0.0466)	0.0172 (0.0831)	-0.1592*** (0.0467)
Branch density	-0.0068 (0.0083)	0.0136*** (0.0024)	-0.0042 (0.0056)	0.0136*** (0.0024)	-0.0049 (0.0045)	0.0136*** (0.0024)
HHI	1.2255 (1.2553)	0.7256** (0.3679)	0.7399 (0.8203)	0.7206** (0.3660)	0.1395 (0.6805)	0.7038* (0.3669)
Cooperative banks	0.0075 (0.0053)	0.0047*** (0.0016)	-0.0015 (0.0035)	0.0048*** (0.0016)	-0.0056** (0.0029)	0.0048*** (0.0016)
NPL ratio	0.0231** (0.0112)	0.0023 (0.0035)	0.0049 (0.0077)	0.0024 (0.0035)	0.0068 (0.0064)	0.0025 (0.0035)
Enforcing Contracts	-0.0218* (0.0115)	-0.0096*** (0.0033)	-0.0157** (0.0074)	-0.0096*** (0.0033)	-0.0168*** (0.0059)	-0.0096*** (0.0033)
Increased production costs		-0.0427 (0.0354)		-0.0445 (0.0353)		-0.0439 (0.0353)
Pessimistic expectations		0.4077*** (0.0388)		0.4108*** (0.0387)		0.4134*** (0.0388)
Intercept	-4.0219*** (0.9490)	-1.3184*** (0.2441)	-1.8610*** (0.5996)	-1.3231*** (0.2435)	-1.6670*** (0.4910)	-1.3218*** (0.2439)
Time fixed-effects	Yes	Yes	Yes	Yes	Yes	Yes
Country group fixed-effects	Yes	Yes	Yes	Yes	Yes	Yes
Initial conditions						
$R^*_{0_i}$	0.8959*** (0.2853)		0.5860*** (0.1531)		0.5703*** (0.1074)	
D_{0_i}		0.4482*** (0.0451)		0.4421*** (0.0450)		0.4415*** (0.0450)
Random effects						
σ_{a_2}	1.0582*** (0.0763)		0.7858*** (0.0621)		0.6778*** (0.0535)	
σ_{a_1}	0.6456*** (0.0233)		0.6446*** (0.0234)		0.6460*** (0.0234)	
$\rho_{a_1 a_2}$	-0.1355 (0.1026)		-0.1887 (0.1176)		-0.1156 (0.1138)	
Idiosyncratic errors						
$\rho_{\varepsilon\mu}$	-0.3292*** (0.0743)		-0.3303*** (0.0607)		-0.3513*** (0.0696)	
Total correlation (ρ_{tot})	-0.2080*** (0.0683)		-0.2915*** (0.0591)		-0.2795*** (0.0634)	
Number of observations	14452		14452		14452	
Log-likelihood	-8643.73		-9265.10		-9835.03	

Notes: the Table reports the coefficients of the extended dynamic model *dyn_c)* estimated using a one-year time frame (instead of six-month time frame) for the lagged dependent variables and using, alternatively, *Rationing*, *Rationing 2* and *Rationing 3* as indicators of financing constraints. Standard errors are reported in parentheses. *Large*, *≥10 years*, *Micro loan* and *Industry* are used as base levels for the categorical variables of firm size, firm age, loan size and sector, respectively.

***, **, * denote significance at 1, 5 and 10% levels, respectively.
